# Vibrational spectra of formic acid and its dimer: I. Spectroscopic accuracy through matrix-isolation IR spectroscopy and anharmonic computations

**DOI:** 10.1039/d6cp01818a

**Published:** 2026-07-22

**Authors:** Dennis F. Dinu, Lukas Meinschad, Jonas Schlagin, Vincent Enders, Maren Podewitz, Dominik Stolzenburg, Guntram Rauhut, Thomas Loerting, Hinrich Grothe, Klaus R. Liedl

**Affiliations:** a Institute of Materials Chemistry, TU Wien Getreidemarkt 9 1060 Wien Austria; b Department of General, Inorganic and Theoretical Chemistry, University of Innsbruck Innrain 80 6020 Innsbruck Austria klaus.liedl@uibk.ac.at; c Department of Physical Chemistry, University of Innsbruck Innrain 52 6020 Innsbruck Austria; d Institute of Theoretical Chemistry, University of Stuttgart Pfaffenwaldring 55 70569 Stuttgart Germany

## Abstract

This work constitutes part I of a series on vibrational spectroscopy of formic acid. As the simplest carboxylic acid, formic acid and its cyclic dimer are well-established models for investigating vibrational signatures of hydrogen-bonded organic acids. This has led to numerous highly specialized studies on formic acid, rendering the field increasingly fragmented. Here we demonstrate how a straightforward combination of experimental and theoretical approaches can provide a complete description of the vibrational structure of formic acid. Using matrix-isolation Fourier-transform infrared (MI-FTIR) spectroscopy with argon and neon as hosts, we record mid-IR spectra of HCOOH, HCOOD, DCOOH, and DCOOD and assign all fundamental bands, as well as numerous combination bands, overtones, and resonances. Matrix-induced frequency shifts in neon average about 2 cm^−1^, with maximum shifts of 6 cm^−1^, making neon a close approximation to the gas phase IR spectrum. Anharmonic vibrational calculations based on a high-quality potential energy surface (PES) reproduce gas phase reference data with mean absolute deviations of ∼2 cm^−1^ for the monomer and ∼4 cm^−1^ for the dimer. Taken together, the calculations approach spectroscopic accuracy for the matrix-isolation data. We demonstrate this accuracy for the *trans*-formic acid monomer and its deuterated isotopocules (HCOOH, HCOOD, DCOOH, DCOOD) as well as for the cyclic non-polar dimer in *C*_2h_ symmetry ((HCOOH)_2_, (DCOOD)_2_), using both vibrational perturbation theory (VPT2) and vibrational configuration interaction theory (VCI). Our results reconnect the diverse threads of formic acid IR spectroscopy and establish a framework for interpreting matrix-, isotope-, and cluster-induced frequency shifts.

## Abstract 2

Matrix-isolation FTIR (Ar, Ne) and high-level anharmonic VPT2/VCI calculations deliver a complete mid-IR assignment of formic acid and its dimer. Isotope and- matrix shifts are quantified for H/D isotopologues with near-spectroscopic accuracy.

## Graphical abstract

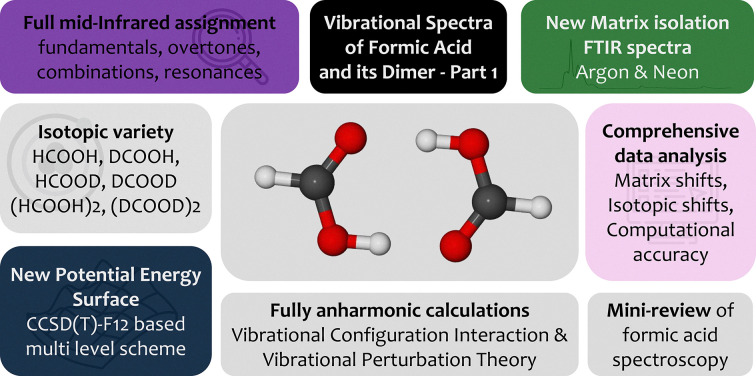

## 1 Introduction

To systematically address the challenges of vibrational spectroscopy of molecular clusters, we present a series of studies focusing on formic acid as a prototypical system. In this first part (part I), we establish a methodological benchmark, while subsequent parts will extend this framework to increasingly complex aspects, including conformational diversity (part II). We use Fourier-transform infrared spectroscopy to obtain broadband vibrational spectra of matrix-isolated formic acid and its dimer, and perform semi-automated anharmonic vibrational frequency calculations based on established perturbative and variational treatments. This strategy provides a routine laboratory framework that allows straightforward access to the vibrational structure, albeit in the presence of matrix-induced effects.^1^ By contrast, gas phase spectroscopy of comparable vibrational detail has led contemporary investigations to rely increasingly on highly specialized instrumentation and tailor-made computational methodologies, rendering such approaches far from routine.^2^ While these developments reflect the maturity of the field and set the benchmark, they have also made it increasingly difficult for non-specialists to maintain an overview. Spectroscopic nomenclature has become highly specialized, computational approaches are often difficult to compare, and experimental particularities can introduce effects that may or may not be captured by theory. Spectroscopy of formic acid has developed into a highly specialized area of research, and we outline its evolution in the following literature review by highlighting key historical and methodological developments.

### Conventional absorption spectroscopy of formic acid

The fundamental vibrational features of formic acid (HCOOH) were first determined by comparing spectra from structurally similar organic molecules. In 1926, Harris reported the C–O vibration in the absorption spectrum of formic acid vapor.^3^ In the spectra, Harris already distinguished the single molecule, *i.e.*, the HCOOH monomer, and the double molecule, *i.e.* the (HCOOH)_2_ dimer. Later, Stansfeld identified the O–H vibration in the spectra of both the vapor and liquid forms of formic acid.^4^ These identifications and assignments of overtones and combination bands, *e.g.*, in liquid formic acid by Roth in 1934,^5^ implied basic concepts of molecular vibration. However, these early spectroscopic studies lacked a sound theoretical foundation, presumably because a valid structural model was unavailable.

With the hydrogen-bonding concept proposed by Latimer and Rodebush in 1920,^6^ the carboxyl group in formic acid became a prototype for studying such intermolecular interactions. The double-hydrogen bond in the (HCOOH)_2_ dimer was experimentally verified by Pauling and Brockway in 1934^7^ using electron diffraction. This structural model of the so-called *trans*–*trans* dimer led to the first quantum-mechanical treatment of hydrogen bonding in 1936^8^ and facilitated Bonner's first distinction between monomers and dimers in the gas phase IR spectrum of formic acid in 1938.^9^ Deuterated species were also investigated at that time.^10,11^ However, these spectra were of mediocre resolution, and the assignment of the individual spectral bands remained superficial.

With an echelette grating spectrometer, Williams achieved a resolution (∼10^−1^ cm^−1^) that enabled the first investigation of the rotational fine structure of the monomer in 1947.^12,13^ The “higher” resolution motivated the development of new theoretical models, *e.g.*, by Thomas in 1950, who presented the first assignment of actual vibrational modes, based on a valence force field.^14,15^ Later, Wilmshurst reassigned the fundamental frequencies of the monomer, using proper chemist's notations and normal mode symmetry.^16^ Concurrently with these proceedings in the assignment of the monomer vibration, in the 1950s, Hadzi and coworkers advanced the understanding due to dimer formation of the carboxyl group, providing the first systematic IR shifts due to hydrogen bonding.^17–20^

### Gas phase *vs.* matrix-isolation spectroscopy

By the end of the 1950s, Millikan and Pitzer further improved the assignment of the monomer in the gas phase,^21^ the dimer in the gas phase, and the crystal.^22^ This included a study that features the first matrix-isolation infrared (MI-IR) spectroscopy of formic acid in nitrogen, together with a normal coordinate analysis for the out-of-plane modes.^23^ The Millikan-Pitzer assignment of formic acid remained the standard for 20 years (*cf.* Shimanouchi tables^24^). Based on this reference and in the spirit of the model by Thomas,^15^ various types of valence and Urey-Bradley force fields were developed by Nakamoto and Kishida,^25,26^ Brooks *et al.*,^27^ Susi *et al.*,^28^ and Fukushima,^29^ fostering the structure model of formic acid.

In 1977, Redington investigated an extensive set of isotopically substituted formic acids, utilizing MI-IR spectroscopy in neon matrices.^30^ This demonstrated MI-IR spectroscopy as a fruitful resource for studying “pure” vibrations of both monomer and dimer. In the late 1970s, a deeper understanding of the theoretical models was achieved by incorporating *ab initio* calculations to derive semi-empirical force fields. Bosi *et al.* attributed the large splitting of the C=O stretching frequencies in the formic acid dimer to a dynamical charge transfer through the hydrogen bond.^31^ Ha *et al.* quantified the “bias” of mixing *ab initio* data into the force field.^32^ Bock *et al.* developed semi-empirical force fields to distinguish better the vibrations of the *trans*-formic acid and its higher-energy counterpart, the *cis*-formic acid.^33^

The conceptual pinnacle of combining MI-IR spectroscopy, semi-empirical force-field parametrization, and *ab initio* self-consistent field calculations of geometries and harmonic frequencies was achieved in 1987 by Henderson.^34^ He obtained MI-IR spectra of various formic acid isotopocules in multiple matrices (Ar, Kr, Xe, N_2_, CO, CO_2_, O_2_) and elaborated on spectral changes due to the embedding medium, known as matrix effects. Additionally, Henderson provides previously unassigned spectral features for the cyclic dimer. Notwithstanding these earlier efforts, the first peer-reviewed journal article of formic acid in an argon matrix were only published in 1994. In that year, Reva *et al.*^35^ reported MI-IR spectra obtained with a conventional absorption spectrometer, while Lundell *et al.*^36^ presented the first matrix-isolation Fourier-transform infrared (MI-FTIR) spectrum of formic acid.

Pure vibrational transitions observed *via* MI spectroscopy were of limited use for developing a consistent theoretical model, as already noted by Redington^30^ and analyzed in detail by Henderson.^34^ While gas phase monomer assignments were gradually refined, notably through Raman spectroscopy by Bertie and Michaelian in 1982,^37,38^ the most substantial progress at that time concerned the dimer. In 1987, Maréchal revised the gas phase assignments of both monomer and dimer and provided important insights into proton transfer in the cyclic dimer.^39^ These studies were experimentally demanding, primarily because controlling dimerization in the gas phase was difficult. As a consequence, post-experimental decomposition of Fourier-transform infrared (FTIR) spectra into their individual components was introduced, enabling improved assignments of the dimer in the gas phase.^40^ A particularly productive period in high-resolution FTIR spectroscopy in the gas phase followed around the turn of the millennium, with extensive work on *trans*-HCOOH,^41–46^*trans*-DCOOH,^47–51^*trans*-HCOOD,^52–55^ and *trans*-DCOOD.^37,55–58^

### IR-pumping & pulsed supersonic jets

The challenges associated with IR spectroscopy in the gas phase also explain why the “other rotamer of formic acid”, *i.e.*, *cis*-conformer, was not observed. Instead, it was first characterized using microwave spectroscopy in 1976.^59,60^ Due to its higher energy, the *cis*-conformer is less populated^23^ and therefore harder to detect, particularly in the gas phase, where dimerization complicates the spectra. Although Miyazawa *et al.* already considered this conformer in their early MI-IR studies,^23^ its definitive infrared observation came considerably later.

It was not until 1997 that Pettersson *et al.* reported the first comprehensive IR assignment of *cis*-formic acid in argon.^61^ The authors used narrow-band IR pumping of the OH-stretching overtone, which populates the higher-energy *cis*-conformer in the matrix. Later, they estimated that the *cis*- to *trans*-formic acid conversion in argon matrices is fast even at low temperatures, taking place within minutes at 8 K, and proceeds *via* a pure tunneling mechanism.^62^ Since 2003, the Helsinki group further studied the *cis*- and *trans*-conformer and its interconversion using the IR pumping approach for all deuterated isotopocules of formic acid isolated in various matrix materials, with a focus on the monomer^63–69^ or the different *cis*- and *trans*- formic acid combinations that form dimers.^70–73^ In 2009, they provided the first Raman spectrum of formic acid in argon, assigning the monomer, cyclic, and acyclic dimer.^74^

Conventional matrix-isolation relies on the continuous deposition of the host-guest mixture. However, different techniques are useful for better understanding matrix effects. For example, in 2009, the rapid vapor codeposition of independent gas flows of formic acid and enriched *para*-H_2_ enabled Poulson and Anderson to understand the effects of *ortho*/*para*-H_2_ on the vibrational frequencies of formic acid.^75^ Another more influential technique, however, was presented in 1998 by Halupka and Sander.^76^ They demonstrated improved control over dimerization during matrix-isolation by using a pulsed matrix deposition from a supersonic jet expansion.^76^ The supersonic jet expansion adiabatically cools the gas mixture, and the pulsed matrix deposition promotes dimer formation. Ultimately, this approach enabled the identification of open-chain dimers in 1999.^77,78^ Such open-chain intermediates are considered as structural building blocks in liquid and solid formic acid.^78^ The jet-to-matrix approach also demonstrated the formation of hydrogen bonds between formic acid and water.^79^ In 2008, Ito used the pulsed jet-to-matrix deposition to investigate formic acid aggregates.^80,81^

Beyond the use of supersonic jets in matrix deposition, spectroscopy of the jet itself has become a valuable complement to conventional matrix-isolation and gas phase techniques. First spectra of jet-cooled formic acid were obtained in 1999 by Merker *et al. via* optothermal detection of laser absorption, resolving the *ν*C=O region.^82^ In the early 2000s, Ito and Nakanaga presented a cavity ring-down spectrum of jet-cooled formic acid, assigning resonances and combination bands in the *ν*C–H region^83^ and in the *ν*O–H region.^84^ In 2002, Madeja *et al.* picked up the jet with Helium nanodroplets to study the *ν*C–H and *ν*O–H regions *via* depletion spectroscopy,^85^ and subsequently observed the acyclic dimer in the Helium nanodroplet *via* diode laser spectroscopy.^86^ Concurrently, gas phase FTIR spectra with medium to high resolutions (up to 10^−3^ cm^−1^) were established by Hurtmans *et al.* (2000),^87^ Freytes *et al.* (2002),^41^ and Georges *et al.* (2004)^88^ who also obtained FTIR spectra of jet-cooled formic acid over a range of 1850 to 3750 cm^−1^. Increased experimental resolution also enabled a more detailed investigation of the proton-tunneling splitting in the formic acid dimer.^89^ Considering such studies, we may refer to the 2009 review by Birer and Havenith for further details.^90^

Over the last two decades, Suhm and coworkers have conducted numerous Raman and FTIR spectroscopy studies of jet-cooled formic acid dimers and trimers.^2,91–98^ Zielke and Suhm initiated these studies in 2007 with the first Raman spectroscopy investigation of jet-cooled formic acid, which characterized Raman-active vibrations below 700 cm^−1^, specifically three inter- and one intra-monomer modes.^99^ Besides further characterization of overtones in this spectral region of formic acid,^91^ the spectroscopy of jet-cooled carboxylic acids implied that “stiffest hydrogen bonds give rise to the most weakly bound dimers.”^92^ Far IR spectroscopy of jet-cooled formic acid allowed observation of the “last missing” inter-monomer hydrogen stretch vibration, and enabled refining the vibrational partition function of the dimer.^93^ FTIR and Raman spectra of the *ν*C=O vibration of monomers and dimers of jet-cooled formic, acetic, and pivalic acid were compared with *ab initio* frequency calculations, showing that the harmonic approximation for the molecular vibration reasonably predicts frequency shifts.^100^ Raman spectra of the jet-cooled *cis*-formic acid were obtained by thermal excitation of the *trans*-conformer *via* heating of the nozzle during jet expansion.^101^ Ultimately, Nejad *et al.* extended the vibrational database for formic acid and its deuterated isotologues using Raman spectroscopy of jet-cooled formic acid and *ab initio* calculations.^96,102,103^ For a more comprehensive summary of the achievements by the Göttingen group, we may refer to the dissertations by Xue,^104^ Kollipost,^94^ Meyer,^95^ and Nejad.^2^

### Computing the formic acid vibrational spectrum

Concurrently with advances in experimental techniques, efforts toward *ab initio* anharmonic calculations of the vibrational transitions of formic acid have intensified over the past decade. Kelemen and Luber recently reviewed the implications of different computational approaches for the formic acid monomer.^105^ Computational improvements relevant to vibrational spectroscopy can be broadly categorized into two extremes. First, enhancing the accuracy of the potential energy surface (PES), typically by progressing from density functional theory (DFT) through Møller–Plesset perturbation theory of second order (MP2) to coupled-cluster methods such as CCSD(T). Second, refining the molecular Hamiltonian to obtain anharmonic vibrational frequencies. Beyond the harmonic approximation, anharmonic methods that rely on solving the time-independent Schrödinger equation are usually classified as perturbative, such as second-order vibrational perturbation theory (VPT2), or variational, including the vibrational self-consistent field (VSCF) and vibrational configuration interaction (VCI) approaches.^106–108^

A broader discussion of these aspects, encompassing both the monomer and the dimer, is provided by Nejad,^2^ who notes that “[…] the stories of formic acid and its cyclic dimer are certainly such cases, where a comparison of harmonic, VPT2, and VCI term values could have revealed shortcomings in the latter.” In other words, a balanced assessment employing multiple computational approaches appears necessary for in-depth analysis. Thus, we briefly summarize key developments in the computational spectroscopy of formic acid, highlighting the essential methodological progress and benchmark studies that inform the present work. For clarity, we simplify this overview by using the acronyms introduced above and omitting detailed discussions of computational subtleties, *e.g.*, density functionals or basis sets.

A first calculation of formic acid within a variational anharmonic approach was done in 2000 by Wright *et al.*, who constructed a PES at the DFT level of theory and used correlation-corrected VSCF for calculating the anharmonic frequencies, with a relatively large deviation from experiment.^109^ The Helsinki group around Maçôas *et al.* (2003) used the same correlation-corrected VSCF approach, but now on a PES at the MP2 level of theory.^63^ The calculation supported their assignment of the mid- and near-IR transitions of *cis*- and *trans*-HCOOH in argon matrices, reproducing most fundamentals reasonably well, except for the torsional and deformation modes, which were significantly underestimated. Strong anharmonic effects and unresolved Fermi resonances limited the accuracy of computed overtones and combinations, particularly for *cis*-HCOOH. In 2007, for *trans*-HCOOH, Demaison *et al.* modeled the PES as a semi-quartic force field using up to CCSD(T) level of theory and computed anharmonic frequencies *via* VPT2.^110^ These calculations, based on perturbation theory, enabled the interpretation of Fermi and Darling–Dennison resonances *via* the force constants. However, while some frequencies aligned with experimental values, others matched qualitatively.

In 2013, Mizukami and Tew developed a local PES, *i.e.*, expanded around the *trans*-formic acid using CCSD(T)-F12 level of theory.^111^ They performed anharmonic vibrational calculations with a broad range of methods. Their study demonstrated a clear progression in accuracy, with mean absolute deviation from experimental data ranging from 21 cm^−1^ for VSCF to as low as 9 cm^−1^ using a second-order multi-reference perturbation approach. A key conclusion of this work was that achieving even higher accuracy depends not only on the quality of the PES or the sophistication of the anharmonic method, but also on the choice of coordinates. In particular, the torsional mode in formic acid poses challenges for rectilinear coordinate descriptions and requires alternative approaches to accurately capture both the *cis*- and *trans*-formic acid.

Consequently, in 2016, Tew and Mizukami presented the first global PES connecting both *trans*- and *cis*-formic acid, and computed anharmonic frequencies using an internal coordinate reaction path Hamiltonian.^112^ This approach significantly reduced deviations from experiment, achieving root-mean-square errors as low as 3 cm^−1^, thereby surpassing the accuracy of previous methods based purely on rectilinear coordinate systems. Another global PES, including both conformers, was presented by Richter and Carbonnière in 2018, at the CCSD(T) level of theory.^113^ Their representation of the PES in internal coordinates and the corresponding formulation of the vibrational Hamiltonian led to results in good agreement with those of Tew and Mizukami.

Because these PESs are computationally expensive, it has become common practice to reuse them for different types of vibrational calculations, thereby enabling the isolated investigation of the impact of various anharmonic approaches while maintaining consistent PES quality. For example, the Mizukami–Tew PES was later employed by Nejad and Sibert^103^ and by Martín Santa Daria *et al.*^114^ and Avila *et al.*^115^ The Richter–Carbonnière PES was subsequently used by Aerts *et al.*,^116,117^ as well as again by Nejad and Sibert.^103^ To this point, we have discussed studies that focus primarily on the monomer; however, there are also notable studies on the dimer. One example is the 2016 study by Qu and Bowman, who developed a PES at the CCSD(T) level of theory,^118^ which they later employed in a series of anharmonic calculations, most notably using VCI.^119,120^ This PES was also the basis for calculations of the fingerprint region of the dimer by Martín Santa Daria *et al.*^121^ All these efforts have progressively improved the *ab initio* calculation of vibrational frequencies for formic acid, achieving increasingly good agreement with gas phase reference data.

In recent years, machine-learning approaches have been employed to construct potential energy surfaces (PESs) for calculating both harmonic^122,123^ and anharmonic^123,124^ vibrational spectra of the *trans*-formic acid monomer, as well as harmonic^122,125,126^ and anharmonic^122,123,125^ spectra of the cyclic dimer. However, the accuracy of these calculations remains limited to VPT2 approaches and cannot yet match that of the most accurate *ab initio* calculations.

Unlike spectroscopy in the gas phase or supersonic jets, spectroscopy in matrix-isolation introduces effects that are difficult to capture in theoretical models. Since Redington's seminal work in 1977,^30^ it has been clear that force fields cannot be reliably derived from matrix data. Since then, several studies have attempted to compute matrix-induced frequency shifts for formic acid using quantum chemical methods. Trakhtenberg *et al.* (2009) modeled the monomer in an Ar_12_ cage to study tunneling behavior,^127^ but their predicted frequency shifts significantly overestimated experimental values. More recently, Stepanian and Adamowicz (2020) modeled the formic acid dimer in Ne_*n*_, Ar_*n*_ (*n* = 171), Kr_*n*_, and Xe_*n*_ (*n* = 108) clusters using fully *ab initio* methods and likewise reported good agreement between experiment and theory.^128^

Similarly, Ito (2010) examined the formic acid dimer in various Ar_*n*_ (*n* = 18–26) clusters and observed variations in solvation energies depending on the substitution site and crystallographic plane, yet the calculated shifts still exceeded the observed shifts.^129^ He attributed this discrepancy to the neglect of additional solvation shells. In 2019, Ito extended his model to include larger clusters (Ar_*n*_ with *n* = 26, 94, 361, 662, 1095), treating outer shells with a universal force field and inner shells using quantum chemistry, thereby improving agreement with experiment.^130^ Nevertheless, such explicit inclusion of the matrix environment in vibrational frequency calculations remains computationally demanding and does not yet yield uniformly reliable predictions across the full spectral range.

### The “gas phase” vibrational spectrum of formic acid

There is no single, unified “gas phase” vibrational spectrum of formic acid. Instead, the fundamental vibrational transitions of formic acid in the gas phase can be assembled from numerous individual experiments. In 2022, Nejad compiled, revised, and extended the list of vibrational frequencies for the *trans*-formic acid monomer (hereafter referred to as the gas-phase reference dataset by Nejad). Besides own contributions *via* spectroscopy of jet-cooled formic acid,^2,96,103^ he also relied on more then twenty different high-resolution investigations of the gas phase species conducted over roughly four decades by various research groups; seven publications on *trans*-HCOOH,^41–45,131,132^ seven publications on *trans*-DCOOH,^38,47–51,133^ seven publications on *trans*-HCOOD,^38,52–55,131,134^ and six publications on *trans*-DCOOD.^12,37,55–58^ In other words, establishing a list of high-resolution gas phase reference values requires consulting nearly one dedicated publication per vibrational state. For the formic acid dimer, the situation becomes even more involved. High-resolution studies typically focus on narrow spectral windows, as the assignment of rovibrational structure and the determination of vibrational band centers are experimentally and analytically demanding. In several cases, only jet-expansion techniques enabled the identification of precise band centers, and in some instances, even synchrotron radiation was required to render weak transitions observable.

Over the past decade, efforts to obtain even more detailed information have led to increasingly specialized and refined experimental approaches. Hull *et al.* (2019) reported synchrotron-based infrared measurements of the formic acid monomer, specifically addressing Fermi resonances in the low-frequency region.^135^ Using infrared depletion spectroscopy, Meyer *et al.* (2020) confirmed the stability of the acyclic formic acid dimer in helium nanodroplets.^136^ Doney *et al.* (2022) investigated the OH stretch of *cis*-formic acid at unprecedented resolution in a supersonic jet using a combination of single-mode laser sources.^137^ Nejad *et al.* (2023) probed the infrared spectrum of the jet-cooled formic acid trimer in the C–O stretch region using a quantum cascade laser.^138^ While these examples are undoubtedly important for methodological development, they also illustrate the direction in which experimental formic acid spectroscopy has evolved, namely, toward greater technical refinement and a progressively specialized niche.

### Towards routinely integrating experiment and theory

Against this backdrop of increasing specialization, we deliberately chose a more integrated and accessible perspective. Rather than targeting isolated spectral windows with highly specialized setups, we perform a systematic sweep across the mid-infrared region for four isotopocules of formic acid, including their dimers, thereby observing nearly the complete set of fundamental vibrational transitions within a coherent laboratory framework relying on matrix-isolation infrared spectroscopy. We employ transparent nomenclature, a systematic analysis of matrix-induced effects, and consistent comparison with high-level anharmonic calculations. In doing so, we demonstrate that a practical combination of routine experiments and predictive theory yields a coherent and broadly applicable description of the vibrational structure of formic acid and its isotopocules, achieving essentially the same spectroscopic information in a fraction of the time required by more specialized approaches that focus on the “gas phase” vibrational spectrum.

## 2 Methodology

### Matrix-isolation FTIR spectroscopy

The matrix-isolation (MI) setup used in this study has been described previously (see Fig. 1 in ref. 1). The rotatable Gifford–McMahon cryostat can operate at a pressure of 10^−7^ mbar and a temperature of 5.8 K. It contains a gold-coated copper mirror for matrix deposition and a PID-controlled heater for temperature regulation. A radiation shield minimizes thermal fluctuations, and an actively isolated optical table suppresses floor-transmitted vibrations in the critical low-Hz range.

**Fig. 1:**
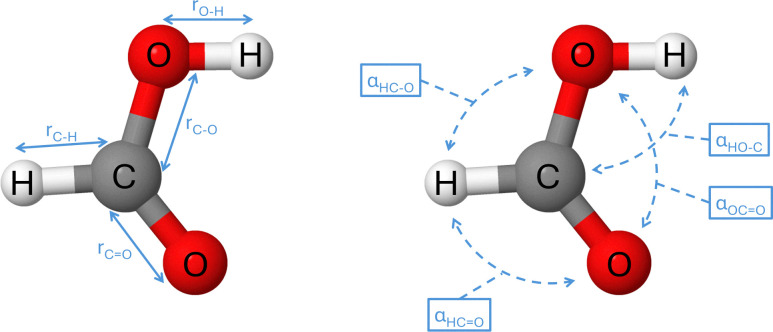
Structural parameters of *trans*-HCOOH in terms of internal coordinates, with bond length coordinates on the left and bond angle coordinates on the right.

Fourier-transform infrared (FTIR) spectra were recorded using a Bruker Vertex 80V FTIR spectrometer (Bruker, Karlsruhe) operated under vacuum (∼2 mbar) to eliminate atmospheric signals (H_2_O and CO_2_). Light from the mid-IR globar source is directed *via* beam splitters and mirrors to the cryostat, reflected off the gold-coated mirror, and returned to a liquid nitrogen-cooled Mercury–Cadmium–Telluride detector. To prevent instrument-transmitted vibrations, a gap of ∼2–3 cm was maintained between the spectrometer and cryostat, and the gap was purged with dry nitrogen to avoid atmospheric signals. The interferometer, equipped with a linear air-bearing scanner and calibrated using a HeNe laser, acquired spectra at a resolution of 0.3 cm^−1^ after averaging 512 scans. Spectra were measured in the 8000–500 cm^−1^ range; background spectra at 5.8 K were subtracted from sample spectra. All measurements were performed under identical conditions to ensure comparability.

High-purity liquid samples of HCOOH (98%, Sigma-Aldrich, 1.00264), HCOOD (98%, Eurisotop, DLM-285-5), DCOOH (98%, Eurisotop, DLM-743-5), and DCOOD (98%, Sigma-Aldrich, 426229) were handled under rare gas flow to avoid atmospheric contamination. About 2 mL of sample was transferred *via* sterile syringe into a pre-flushed glass flask mounted directly to the mixing chamber and subsequently degassed through three freeze-pump-thaw cycles. The vapor above the liquid was evacuated immediately before mixing to ensure the use of freshly evaporated material. *In situ* isotope exchange at the formic acid carboxyl group with surface protons on the stainless steel mixing line generated minor impurities, reducing the initial 98% isotopic purity.

Mixtures with host gases were prepared in a stainless-steel mixing chamber (volume ∼2 L) maintained at 298 K and evacuated to 10^−5^ mbar using a turbomolecular pump in series with a rotary vane pump. The chamber, equipped with multiple gas inlets, enabled sequential or simultaneous introduction of host gases. Barometric monitoring enabled precise control of guest-to-host dilutions at ratios of 1 : 250, 1 : 500, and 1 : 1000, corresponding to approximately 2 mbar of formic acid mixed with 990–1010 mbar of argon or neon. From a ∼200 mL volume and total pressure of 900–980 mbar, the mixtures were deposited using two distinct variants: (a) slow deposition at a controlled rate of 4 mbar per min ≈ 3 × 10^−4^ mol min^−1^ (or over approximately 50 min per layer *via* a mass flow controller to produce uniform layers and higher sample deposition). (b) Fast deposition at roughly 200 mbar per min ≈ 2 × 10^−2^ mol min^−1^ without a mass flow controller, which drastically reduces isotopic exchange between the sample and water impurity, but deposits less sample per layer.

### Theoretical vibrational spectroscopy

All *ab initio* calculations were performed using the molpro 2025 software package.^139^ We calculated the Born–Oppenheimer equilibrium geometry of the *trans*-formic acid monomer in *C*_s_ symmetry and the *trans*–*trans* dimer in *C*_2h_ symmetry at the CCSD(T)-F12b level of theory,^140^ using the cc-pCVTZ-F12 basis set,^141–143^ including all-electron (ae) correlation and the Goodson continued-fraction (GCF) extrapolation of the coupled-cluster sequence.^144,145^ Using the same level of theory, harmonic frequencies and normal modes were obtained from numerical Hessians. The potential energy surface (PES) was constructed in an N-mode expansion^108,146^ on a grid along the normal mode coordinates. For the *ab initio* single-point energies, we employed the multilevel approaches,^108^ as outlined in Table 1. For the cyclic dimer, we used the same level of theory for the 3- and 4-mode terms but a slightly lower level for the 1- and 2-mode terms. Most noteworthy is the neglect of core correlation in the 1-mode terms for the dimer. The dipole moment surface was computed concurrently; however, in this case, MP2 theory was used throughout to obtain analytical properties.

**Table 1:** N-mode potential energy surface expansion of *trans*-formic acid

N-mode coupling	Method^a^	Basis set^b^
Monomer		
1	(ae)CCSD(T)-F12b + GCF	cc-pCVTZ-F12
2	(fc)DCSD-F12b	cc-pVTZ-F12
3	(fc)DCSD-F12b	cc-pVDZ-F12
4	(fc)MP2	cc-pVTZ
Cyclic dimer		
1	(fc)CCSD(T)-F12a	cc-pVTZ-F12
2	(fc)DCSD-F12a	cc-pVDZ-F12
3	(fc)DCSD-F12a	cc-pVDZ-F12
4	(fc)MP2	cc-pVTZ

a (ae) stands for all-electron and (fc) for frozen-core correlation. GCF indicates that Goodson's continued-fraction extrapolation was applied to the energy.^144,145^ CCSD(T)-F12 stands for coupled cluster with perturbative triples and explicit correlation (F12).^140^ DCSD is the distinguishable cluster approach.^147,148^ MP2 stands for Moller-Plesset perturbation theory of second order.

b The basis sets used are of the Dunning-type,^141^ respectively their adapted version for all-electron correlation^142^ and for the F12 methods,^143^ which inherently include diffuse functions.

The grid representation of the PES was transformed into an analytical polynomial representation by means of Kronecker product fitting.^149^ This polynomial PES was used for state-specific vibrational self-consistent field (VSCF) calculations employing a basis of 18 distributed Gaussians. The resulting VSCF modals served as the basis for vibrational configuration interaction (VCI) calculations. The VCI calculations for the monomer utilize modals derived from ground-state VSCF. In contrast, for the corresponding dimer calculations, state-specific modals were used, resulting in better convergence of the VCI solution. Additionally, using the coefficients of the polynomial representation of the PES, a semi-quartic force field was derived for second-order vibrational perturbation theory (VPT2) calculations, using automated Darling–Dennison and Fermi resonance detection with variational correction for the respective states.^150^

For the monomer, the VCI expansion included excitations up to sextuple level (CITYPE = 6), with a maximum of nine quanta per mode (LEVEX = 9) and a total sum of quantum numbers of (CIMAX = 20). For the DCOOH isotopocule, the ν_4_ mode required an extended configuration space, using (LEVEX = 14), (CIMAX = 24).

Regarding the configuration space of the dimer, excitations up to the sextuple level (CITYPE = 6) were included, with a maximum of five quanta per mode (LEVEX = 5) and a total sum of quantum numbers of (CIMAX = 15). Regarding the treatment of the vibrational angular momentum for both the monomer and the dimer, the constant μ-tensor (0D) was extended by one-dimensional terms, which are added to the diagonal elements of the VCI-matrix.

We derive vibrational notations based on an optimal set of internal coordinates, which was found by normal mode decomposition using the nomodeco toolkit.^151^ To reduce numerical errors, we used analytical Hessians at the HF/6-311G(d,p) level of theory for the normal mode decomposition. For the dimer, the decomposition was performed using an internal coordinate to describe the hydrogen bond.^152^

## 3 Results and discussion

### 3.1 Structural and spectroscopic parameters

The structural parameters, *i.e.*, bond lengths and angles, of the *trans*-formic acid are shown in Fig. 1, with the corresponding values listed in Table 2. We obtain the equilibrium structural parameters (*r*_e_) from geometry optimizations within the Born–Oppenheimer Approximation again at the CCSD(T)-F12b level of theory utilizing the cc-pCVTZ-F12 basis set. The vibrationally averaged structural parameters were obtained from either the atomic positions, which are averaged over the VCI ground state wavefunction (*r*_a_), or from the internuclear distances, which can be derived from the expectation value of the bond lengths expanded in normal coordinates (*r*_g_).^153–155^ We compare our calculated structural parameters with experimentally derived values through rotational spectroscopy (*r*^MW^_0_) by Kwei and Curl,^156^ as well as the electron diffraction data (*r*^ED^_0_) by Almenningen *et al.*^157^

**Table 2:** Structural parameters of *trans*-HCOOH. Bond lengths (*r*) are given in Å and bond angles (*α*) are given in °. Calculated structural parameters (this work) from geometry optimizations (*r*_e_) and from vibrational averaging (*r*_a_, *r*_g_) are compared to literature experimental data from microwave spectroscopy (*r*^MW^_0_) and electron diffraction (*r*^ED^_0_)

Parameter	*r*_e_	*r*_a_	*r*_g_	*r* ^MW^_0_ ^156^	*r* ^ED^_0_ ^157^
*r*_CH_	1.093	1.102	1.115	1.097	1.096
*r*_C=O_	1.198	1.201	1.202	1.202	1.213
*r*_C–O_	1.341	1.349	1.350	1.343	1.357
*r*_OH_	0.967	0.966	0.986	0.972	0.966

*α*_OC=O_	124.86	124.85	—	124.88	123.55
*α*_HC=O_	125.08	125.12	—	124.12	—
*α*_HC–O_	110.06	110.03	—	110.79	—
*α*_HO–C_	106.76	107.17	—	106.83	—

The spectroscopic parameters, *i.e.*, rotational and centrifugal distortion constants, for the *trans*-HCOOH monomer for the vibrational ground-state are shown in Table 3. We compare our calculations to the experimental values obtained from rotational spectroscopy of Cazzoli *et al.*^158^ (referring to their fit including additional data by Chardon *et al.*^159^). The vibrationally averaged rotational constants (*A*, *B*, *C*) are from vibrational configuration interaction theory, and the centrifugal distortion constants (*Δ*, *Φ*, *ϕ*) were obtained from vibrational perturbation theory using Watson's A-reduction in the I^*r*^ representation.^160^

**Table 3:** Spectroscopic parameters given in Watson's A-reduction in the I^*r*^ representation for *trans*-HCOOH. Rotational constants (*A*, *B*, *C*) from atomic positions averaged over the VCI ground state wavefunction, and quartic (*Δ*, *δ*) and sextic (*Φ*, *ϕ*) centrifugal distortion constants from VPT using a semi-quartic force field transformed from the multi-level N-mode PES described in Table 1. The deviation (dev.) is given between the calculation (calc.) within this work and the experimental (exp.) reference from sub-millimeter wave spectroscopy

Parameter	Calc./Hz	Exp.^158^/Hz	Dev./%
10^6^*A*	77499.70	77512.22	0.02
10^6^*B*	12048.48	12055.10	0.05
10^6^*C*	10410.46	10416.11	0.05
10^3^*Δ*_*J*_	9.930	9.994	0.6
10^3^*Δ*_*JK*_	−87.927	−86.222	1.9
10^3^*Δ*_*K*_	1679	1702	1.4
10^3^*δ*_*J*_	1.933	1.949	0.8
10^3^*δ*_*K*_	39.449	42.781	8.4
10^−3^*Φ*_*J*_	13.0	12.7	0.0
10^−3^*Φ*_*JK*_	96.0	131.9	35.4
10^−3^*Φ*_*KJ*_	−10 387	−10 680	2.8
10^−3^*Φ*_*K*_	116 359	120 190	3.3
10^−3^*ϕ*_*J*_	6.0	5.8	0.0
10^−3^*ϕ*_*JK*_	82.0	79.7	2.4
10^−3^*ϕ*_*K*_	13 898	15 740	13.3

As shown in Tables 2 and 3, we observe good agreement with experimentally derived structural and spectroscopic parameters. This agreement is the first indicator for the appropriate quality of the optimized structure and the potential energy surface. In the following, this comparison between theory and experiment is elaborated on the vibrational structure and infrared spectroscopy of formic acid.

### 3.2 Vibrational notations

For the assignment of IR spectra, we distinguish between computational, physicist's, chemist's, and spectroscopist's notations (*cf.* ref. 161). The choice of the vibrational notation defines the narrative that spectral interpretation can convey. The spectroscopist's notation is given as short label ν_*i*_ with the irreducible representation given in parentheses (irrep). For example, the nine normal modes of HCOOH in *C*_s_ symmetry are categorized by the A′ and A″ irreps (*cf.*Table 4). The ordering of the index *i* is determined by the irrep. The ordering of the indices changes for a different system with a different symmetry. For example, the 24 normal modes of the (HCOOH)_2_ dimer in *C*_2h_ symmetry are categorized by the A_g_, B_g_, A_u_, and B_u_ irreps (*cf.*Table 4). It is immediately clear that this notation, with inconsistent indices across systems, is not useful for comparing them. Moreover, different indices in spectroscopist's notation were proposed for the formic acid dimer,^2^ further complicating the comparison between different sources. On the contrary, the chemist's notation allows a more intuitive description of vibrations and comparison of similar vibrations across different systems, *e.g.*, between monomer and dimer species. The chemist's notation uses labels such as *ν* for stretching, *δ*_ip_ for in-plane deformation, and *δ*_oop_ for out-of-plane deformation. These can be readily obtained *via* normal mode decomposition, which reveals the contributions of internal coordinates (ICs) to a normal mode.^151^ Note that the labels, be it monomer or dimer, change upon deuteration. Within the spectroscopist's notation, this would also complicate comparisons among different systems. In chemist's notations, however, the labels change with the isotope, *e.g.*, *ν*OH becomes *ν*OD. Thus, these labels remain comparable, as the principal motion patterns are similar. More details of the IC contributions for all isotopocules are provided in the SI (Section S4 and S5).

**Table 4:** Vibrational notation of the *trans*-formic acid monomer HCOOH in *C*_*s*_ symmetry, and the cyclic *trans*-formic acid dimer (HCOOH)_2_ in *C*_2h_ symmetry^a^

Notation^b^		*ω*	*ω* ^*I*^	*Δ*	IC
***trans*** **-HCOOH in** ***C***_**s**_ **symmetry**					
ν_1_(A′)	*ν*OH	4104	4104	<1	*r*(OH) 99.9%
ν_2_(A′)	*ν*CH	3255	3248	7	*r*(CH) 98.7%
ν_3_(A′)	*ν*C=O	2016	1961	55	*r*(C=O) 70.7%
ν_4_(A′)	*δ*_ip_CH	1540	1291	249	*ϕ*(O=CH) 49.6%
ν_5_(A′)	*δ*_ip_COH	1426	1370	56	*ϕ*(OCOH) 49.8%
ν_6_(A′)	*ν*C–O	1264	1286	22	*r*(C–O) 60.5%
ν_7_(A′)	*δ*_ip_OCO	698	1242	544	*ϕ*(O–CH) 49.0%
ν_8_(A″)	*δ*_oop_CH	1194	1341	147	*γ*(COOH) 98.0%
ν_9_(A″)	*δ*_oop_COH	707	815	108	*τ*(HOCO) 94.8%
***trans*** **-(HCOOH)**_**2**_ **in** ***C***_**2h**_ **symmetry**					
**Raman active**					
ν_1_(A_g_)	*ν*OH	3818	3522	296	*r*(OH) 51%
ν_2_(A_g_)	*ν*CH	3266	3259	7	*r*(CH) 48.8%
ν_3_(A_g_)	*ν*C=O	1926	1892	34	*r*(C=O) 23.1%
ν_4_(A_g_)	*δ*_ip_COH	1563	1627	64	*ϕ*(COH) 12.3%
ν_5_(A_g_)	*δ*_ip_CH	1519	1319	200	*ϕ*(OCH) 16.4%
ν_6_(A_g_)	*ν*C–O	1346	1173	173	*r*(C–O) 11.9%
ν_7_(A_g_)	*δ*_ip_OCO	737	1627	890	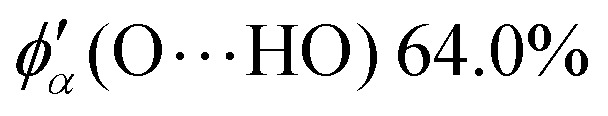
ν_8_(A_g_)	Stretching	179	161	18	
ν_9_(A_g_)	Bending	164	1627	1463	
ν_10_(B_g_)	*δ*_oop_CH	1210	1173	37	*γ*(CHOO) 40.5%
ν_11_(B_g_)	*δ*_oop_COH	910	269	641	*τ*(HOCO) 23.4%
ν_12_(B_g_)	Libration	244	777	533	
**Infrared active**					
ν_13_(A_u_)	*δ*_oop_CH	1213	1173	40	*γ*(CHOO) 44.3%
ν_14_(A_u_)	*δ*_oop_COH	958	269	689	*τ*(HOCO) 21.6%
ν_15_(A_u_)	Bending	167	269	102	
ν_16_(A_u_)	Twisting	75	174	99	
ν_17_(B_u_)	*ν*OH	3864	3522	342	*r*(OH) 51.0%
ν_18_(B_u_)	*ν*CH	3263	3529	4	*r*(CH) 45.9%
ν_19_(B_u_)	*ν*C=O	1978	1892	86	*r*(C=O) 23.2%
ν_20_(B_u_)	*δ*_ip_CH	1553	1319	234	*ϕ*(OCH) 16.4%
ν_21_(B_u_)	*δ*_ip_COH	1499	1627	128	*ϕ*(COH) 20.6%
ν_22_(B_u_)	*ν*C–O	1350	1627	277	*r*(C–O) 11.3%
ν_23_(B_u_)	*δ*_ip_OCO	750	1627	877	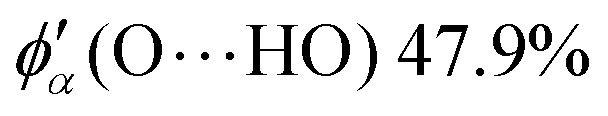
ν_24_(B_u_)	Libration	219	1627	1408	

a Harmonic frequencies (*ω*) in cm^−1^ were calculated using an analytical Hessian at the HF/6-311G(d,p) level of theory. Main internal coordinate (IC) contributions were obtained from this Hessian through normal mode decomposition using nomodeco.^151^*Δ* = |*ω* − *ω*^*I*^|. Internal coordinates are bond lengths (*r*), in-plane angles (*ϕ*), linear angles (*ϕ*′), proper dihedral angles (*τ*), and improper dihedral angles (*γ*).

b ν_*i*_ (irrep) denotes the spectroscopist's notation, with the irreducible representation given in parentheses (A′, A″, A_g_, A_u_, B_g_, B_u_). For the dimer, we use the index sorting proposed by Bertie *et al.*^37^ The chemist's notation uses *ν* for stretching, *δ*_ip_ for in-plane, and *δ*_oop_ for out-of-plane deformation modes. For large amplitude motions in the dimer, we use the nomenclature from ref. 121.

Table 4 summarizes the vibrational notations for the *trans*-formic acid monomer (HCOOH, *C*_s_ symmetry). The absolute difference *Δ* = |*ω* − *ω*^*I*^| between the harmonic (*ω*) and intrinsic (*ω*^*I*^) frequencies^162^ quantifies the degree to which a specific normal mode is described by the motion of a single internal coordinate, thus characterizing the level of delocalization of a normal mode with respect to the selected internal coordinate set. As expected, the local stretching modes *ν*OH (ν_1_) and *ν*CH (ν_2_) show negligible *Δ*, *i.e.*, are almost purely defined by single bond displacements. In contrast, the bending and torsional modes exhibit significantly larger deviations *Δ*, indicating that linear combinations of the selected internal coordinates are necessary to represent their vibrational displacements accurately. Overall, the normal mode decomposition provides a chemically intuitive picture of the vibrational structure, supporting the labels derived in the chemist's notation, such as the characteristic modes *ν*OH, *ν*C=O, *ν*C–O, and *δ*_ip_COH.

Table 4 also lists the vibrational notations for the *trans*-formic acid dimer ((HCOOH) 2, *C*_2h_ symmetry). Here, the normal mode decomposition demonstrates that the contributions to each mode are distributed nearly equally between the two monomer units. Therefore, the analysis of one monomer's internal coordinates suffices to describe the intramolecular modes listed in Table 4. The percentages reported are approximately half of those found for the isolated monomer, because only one monomer's contributions are shown (*cf.* SI for more details). The dimer has 24 normal modes, of which six are dominated by intermolecular motions: ν_8_(A_g_) and ν_9_(A_g_) (intermolecular stretching and in-plane bending), ν_12_(B_g_) (out-of-plane libration), ν_15_(A_u_) (out-of-plane bending), ν_16_(A_u_) (torsional twisting), and ν_24_(B_u_) (in-plane libration). We can group the remaining 18 modes into two sets of nine vibrations that are either Raman-active (A_g_, B_g_) or IR-active (A_u_, B_u_), and within each set the modes share the same chemist's notation as those of the monomer. Each intramolecular vibration thus appears as a pair of modes differing only in symmetry: ν_1_(A_g_) and ν_17_(B_u_) correspond to the OH stretches; ν_2_(A_g_) and ν_18_(B_u_) to the CH stretches; ν_3_(A_g_) and ν_19_(B_u_) to the C=O stretches; ν_4_(A_g_) and ν_21_(B_u_) to the in-plane COH deformations; ν_5_(A_g_) and ν_20_(B_u_) to the in-plane CH deformations; ν_6_(A_g_) and ν_22_(B_u_) to the C–O stretches; ν_7_(A_g_) and ν_23_(B_u_) to the in-plane OCO deformations; ν_10_(B_g_) and ν_13_(A_u_) to the out-of-plane CH deformations; and ν_11_(B_g_) and ν_14_(A_u_) to the out-of-plane COH deformations.

#### Notation of resonances

It is essential to note that, in addition to the fundamental vibrational transitions, for which the notations in Table 4 are given, we also observe overtones and combination bands in the IR spectra. These transitions are typically much weaker in intensity than the fundamentals and therefore play only a minor role in analytical applications. However, overtones and combinations can give rise to quasi-degeneracies among vibrational energy levels, resulting in vibrational resonances. Such interactions may obscure spectral assignments and, if not properly analyzed, compromise the interpretation of experimental data, as we recently discussed for the MI-FTIR spectrum of methanol.^163^ Thus, before delving into the spectroscopic assignment of our MI-FTIR spectra of formic acid, we may elucidate the issue of its resonances at first.

**Fig. 2:**
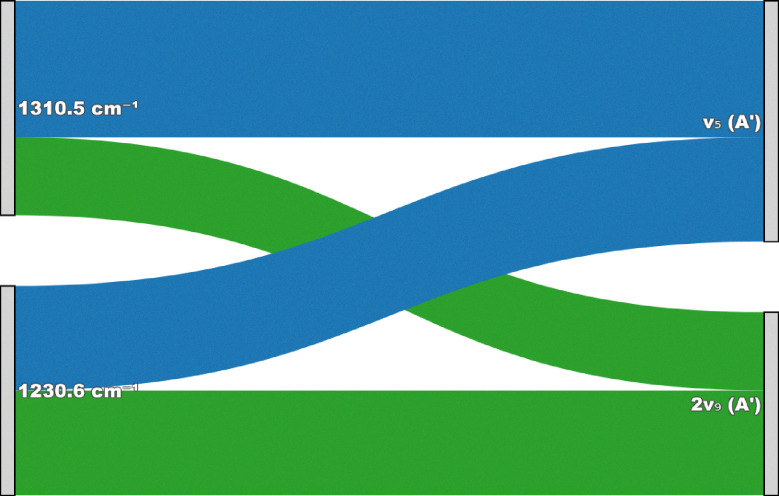
Calculated resonance pair between the fundamental ν_5_(A′) (blue) and the overtone 2ν_9_(A′) (green) for HCOOH from the VCI calculation. The line width indicates the contribution of a given state to the VCI solution.

Let us consider the HCOOH isotopocule of the *trans*-formic acid monomer. Here, a particularly notable Fermi resonance occurs between the fundamental *ν*_5_ and the overtone 2ν_9_. As shown in Fig. 2, both the ν_5_ fundamental and the 2ν_9_ overtone contribute comparably to the VCI-calculated states at 1310.5 and 1230.6 cm^−1^ (respectively 1306.2 and 1220.8 in the gas phase reference). It is therefore necessary to decide which of these frequencies to designate as the fundamental.^102,135^ This decision is particularly relevant when using the specific vibration as reference to calculate spectral shifts, such as during cluster formation. Considering ν_5_(*δ*_ip_COH), one must define which of the two interacting states (see Fig. 2) serves as the reference to evaluate the frequency shifts to the *δ*_ip_OH vibration in the dimer or higher oligomers. This distinction is especially critical here, since the difference between the two transitions is 98.7 cm^−1^.

**Fig. 3:**
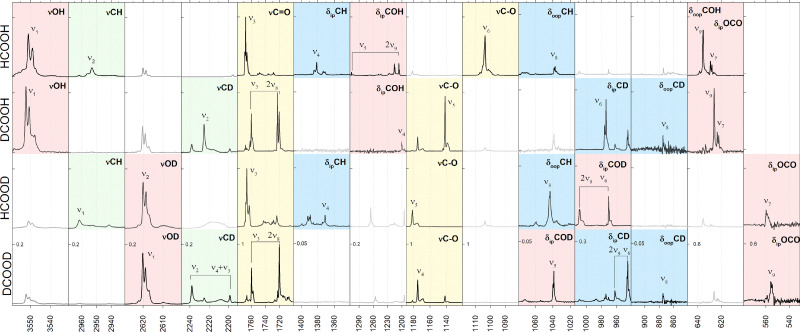
Matrix-isolation FTIR spectra of HCOOH, DCOOH, HCOOD, DCOOD in argon using mixing ratios of approximately 1 : 1000. The relevant spectral regions are cut and highlighted using colors. Only the fundamental bands together with prominent resonances are assigned here. An in-depth assignment of all observed bands is given in the SI, Section S1. Isotopic frequency shifts are directly evident in the colored panels.

Various other resonances can be observed in the MI-FTIR spectra (*cf.*Fig. 3), and their patterns vary across the different isotopocules, as we will show later. From an experimental perspective, such variations demonstrate that isotopic substitution is a valuable tool for identifying and characterizing vibrational resonances. From a theoretical perspective, accurate predictions across isotopocules also serve as a sensitive indicator of a computational model's reliability. For formic acid and its deuterated isotopocules, Nejad *et al.* extensively studied resonances of fundamentals, overtones and combination bands in the context of spectroscopy of jet-cooled formic acid.^2,96,103^ In the present work, we extend this investigation to MI-FTIR spectra.

### 3.3 Assignment of MI-FTIR spectra

Fig. 3 displays the MI-FTIR spectra of formic acid in argon, highlighting regions of the fundamental vibrations. Matrix-isolated formic acid in argon and neon appears relatively well-behaved compared to other host-guest systems. For example, band splitting due to distinct trapping sites in argon, as observed for carbon dioxide,^164^ is not unambiguously observed for formic acid. Some band splitting is present for certain vibrational modes (*e.g.*, *ν*OH, marked red in Fig. 3). Based on matrix-isolation studies on formic acid in argon Maçôas *et al.* have shown that trapping in different matrix sites can lead to site splittings.^63^ These are typically small, on the order of 1–3 cm^−1^ for most vibrational modes in argon, with several modes exhibiting splittings below 1 cm^−1^. Such small separations are often unresolved and typically contribute to band broadening. Consequently, the absence of a resolved splitting for a particular band does not by itself exclude matrix trapping site effects. Rotational-vibrational transitions, as seen for water^165^ and methane,^164^ are also not observed for formic acid, in both argon and neon matrices. This is consistent with Henderson's findings, who also did not observe evidence of rotational-vibrational structure of formic acid in a variety of host matrices.^34^ The torsional barrier from *trans*- to *cis*-formic acid is relatively high (approximately 4800 cm^−1^) in the near-IR, which is why IR pumping of overtones is required to access the *cis*-conformer.^61^ This likely explains why we do not observe torsional-vibrational splitting patterns for formic acid in matrix-isolation. This is in contrast to systems with lower torsional barriers, *e.g.*, methanol exhibiting a torsional band in the far-IR of approximately 370 cm^−1^.^163,166^ Ultimately, the observed bands of formic acid in matrix-isolation are mostly due to pure vibrational transitions.

Similar to the monomer, rotational–vibrational transitions are not observed for the dimer. The line shape does not change upon annealing of formic acid in argon (up to 24 K) and neon (7 K), which would be the case if rotational-vibrational transitions were observed (*cf.* annealing of methane^164^). These experiments merely indicate that the formation of different dimer conformers is likely. Splittings arising from different matrix cage environments can be proposed for certain bands, such as the *ν*C=O stretching mode, as previously noted in the literature.^35^ However, these splittings do not appear to follow a systematic pattern throughout the whole spectrum. Marushkevich *et al.* calculated that the torsional barrier in the dimer is comparable to that of the monomer.^70^ Consequently, spontaneous torsional–vibrational splittings are unlikely to occur in the dimer, just as they are not observed for the monomer. In other words, IR pumping is required to distinguish between the *trans*- and *cis*-variants of the dimer that may arise from induced torsional tunneling.^70^ Beyond torsional tunneling, proton tunneling within the double hydrogen bond of the cyclic dimer may also be considered (*cf.* recent works on double proton tunneling in formic acid and references therein^167–169^). In the vibrational ground state (*i.e.*, without IR excitation), such tunneling could, in principle, lead to proton-tunneling splittings. These splittings have been experimentally observed in the gas phase, for instance by Ortlieb *et al.*,^89^ and are typically on the order of 10^−2^ cm^−1^.^170,171^ However, such values are far smaller than the linewidth typically observed in MI-FTIR spectroscopy. Therefore, although proton-tunneling splittings may occur under matrix-isolation conditions, they remain unresolved in the spectra.

### 3.4 Vibrational frequency shifts

Besides the assignment of absolute band positions, as shown in Fig. 3, we also derive so-called vibrational frequency shifts. Strictly speaking, a vibrational frequency shift occurs when a change in the system influences the energy levels of the vibrational states involved in a specific transition. To evaluate a vibrational frequency shift, it is essential to define the reference frequency from which the shift is measured and the corresponding effect on this frequency. In this work, we distinguish between shifts arising from isotopic exchange, matrix-isolation, and cluster formation.

#### Isotopic frequency shifts

We use the HCOOH isotopocule as a reference spectrum and compare it with the spectra of species in which hydrogen is substituted by deuterium. This comparison defines the isotopic frequency shift. The effect is illustrated by the spectra of the four isotopocules HCOOH, HCOOD, DCOOH, and DCOOD of the formic acid monomer shown in Fig. 3, where we highlight similar vibrations using color. For instance, the shift from the *ν*OH stretching vibration in HCOOH to the corresponding *ν*OD stretching vibration in HCOOD, as observed from gas phase data, would be

1 ^iso^*Δ*^*ν*CD^_Ar_ = *

<svg xmlns="http://www.w3.org/2000/svg" version="1.0" width="13.454545pt" height="16.000000pt" viewBox="0 0 13.454545 16.000000" preserveAspectRatio="xMidYMid meet"><metadata>
Created by potrace 1.16, written by Peter Selinger 2001-2019
</metadata><g transform="translate(1.000000,15.000000) scale(0.015909,-0.015909)" fill="currentColor" stroke="none"><path d="M160 840 l0 -40 -40 0 -40 0 0 -40 0 -40 40 0 40 0 0 40 0 40 80 0 80 0 0 -40 0 -40 80 0 80 0 0 40 0 40 40 0 40 0 0 40 0 40 -40 0 -40 0 0 -40 0 -40 -80 0 -80 0 0 40 0 40 -80 0 -80 0 0 -40z M80 520 l0 -40 40 0 40 0 0 -40 0 -40 40 0 40 0 0 -200 0 -200 80 0 80 0 0 40 0 40 40 0 40 0 0 40 0 40 40 0 40 0 0 80 0 80 40 0 40 0 0 80 0 80 -40 0 -40 0 0 40 0 40 -40 0 -40 0 0 -80 0 -80 40 0 40 0 0 -40 0 -40 -40 0 -40 0 0 -40 0 -40 -40 0 -40 0 0 -80 0 -80 -40 0 -40 0 0 200 0 200 -40 0 -40 0 0 40 0 40 -80 0 -80 0 0 -40z"/></g></svg>


*^*ν*OD^_Ar_(HCOOD) − **^*ν*OH^_Ar_(HCOOH)

and is on the order of 1000 cm^−1^ (*cf.* the red highlighted panels in Fig. 3). A similar magnitude is observed for ^iso^*Δ*^*ν*CD^_Ar_, *i.e.*, the *ν*CH to *ν*CD isotopic shift (*cf.* the green highlighted panels in Fig. 3). In contrast, the ^iso^*Δ*^*ν*C=O^ shift is much smaller, only about 10 cm^−1^ (*cf.* the yellow highlighted panels in Fig. 3). On the other hand, the ^iso^*Δ*^*δ*_ip_CD^ shift and the the ^iso^*Δ*^*δ*_oop_CD^ shifts are on the order of 100 cm^−1^ (*cf.* the blue highlighted panels in Fig. 3). Overall, the isotopic shifts follow an expected systematic trend: the greater the difference in the reduced mass of the normal mode, the larger the frequency shift. We observe the same behavior for the formic acid dimer.

#### Matrix-induced frequency shifts

The influence of the matrix (*e.g.*, argon or neon) on the specific vibrational motions of the trapped molecule leads to matrix-induced frequency shifts. For example, taking the fundamental *ν*OH stretching vibration of the formic acid monomer in the gas phase as a reference, the frequency changes observed upon argon matrix-isolation as

2 ^mat^*Δ*^*ν*OH^_Ar_ = *ν̃*^*ν*OH^_Argon_(Monomer) − *ν̃*^*ν*OH^_Gas_(Monomer)

**Fig. 4:**
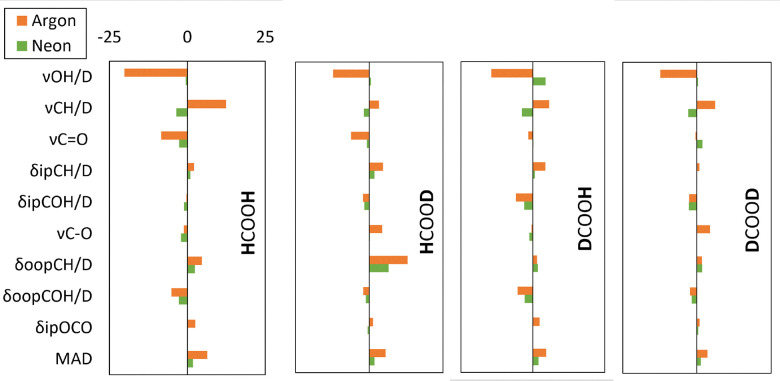
Matrix-induced frequency shifts for the *trans*-formic acid monomer of all isotopocules in argon (orange) and neon (green) matrices as observed in this work. The matrix shifts are calculated according to eqn (2) relative to the gas phase reference dataset by Nejad.^2^ For neon, some deviations are close to zero and are therefore barely visible in the plot. MAD denotes the mean absolute deviation.

In this work, we determine the matrix-induced frequency shifts for argon and neon using the gas phase dataset by Nejad as a reference.^2^ As illustrated in Fig. 4 for the formic acid monomer, we observe non-systematic red- and blueshifts among different fundamental vibrational modes, across distinct isotopocules, and as a function of whether argon or neon is used as the matrix. For instance, whereas the ^mat^*Δ*^*ν*OH/D^_Ar_ shift is consistently large and negative for all isotopocules, the ^mat^*Δ*^*ν*OH/D^_Ne_ shift is close to zero and can be either positive or negative depending on the isotopocule. Conversely, the ^mat^*Δ*^*ν*CH/D^_Ar_ shift is positive for all isotopocules (from 5 to 20 cm^−1^), while the corresponding ^mat^*Δ*^*ν*CD/D^_Ne_ shift is uniformly negative across all isotopes (up to −5 cm^−1^). There are, however, also some consistent cases, *e.g.*, both the 
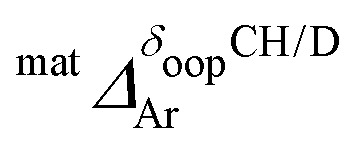
 and 
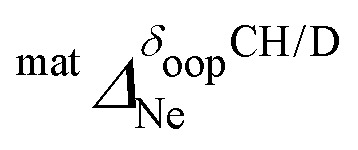
 shifts are consistent blueshifts with up to 5 cm^−1^ for all isotopocules. A closer look at the data further indicates that the shifts are also non-systematic within the same mode across different isotopocules. For example, the *ν*C–O is redshifted in HCOOH and DCOOH, yet blueshifted in HCOOD. These findings further emphasize that the host–guest interactions are more complex than often assumed.

Looking at the mean absolute deviations (MAD) over all fundamental vibrations of HCOOH, the argon shifts are generally larger than the neon shifts, with MAD_Ar_ ≈ 6 cm^−1^ and MAD_Ne_ ≈ 2 cm^−1^. Most strikingly, the argon matrix shows larger extrema (up to −20 cm^−1^) than the neon matrix (up to +4.0 cm^−1^). This is consistent with our earlier observations on other small molecules.^163–165,172,173^ Considering the matrix shifts for all isotopocules, we observe very similar values within one isotopocule. In general, argon matrix shifts are larger than those observed in neon.

**Fig. 5:**
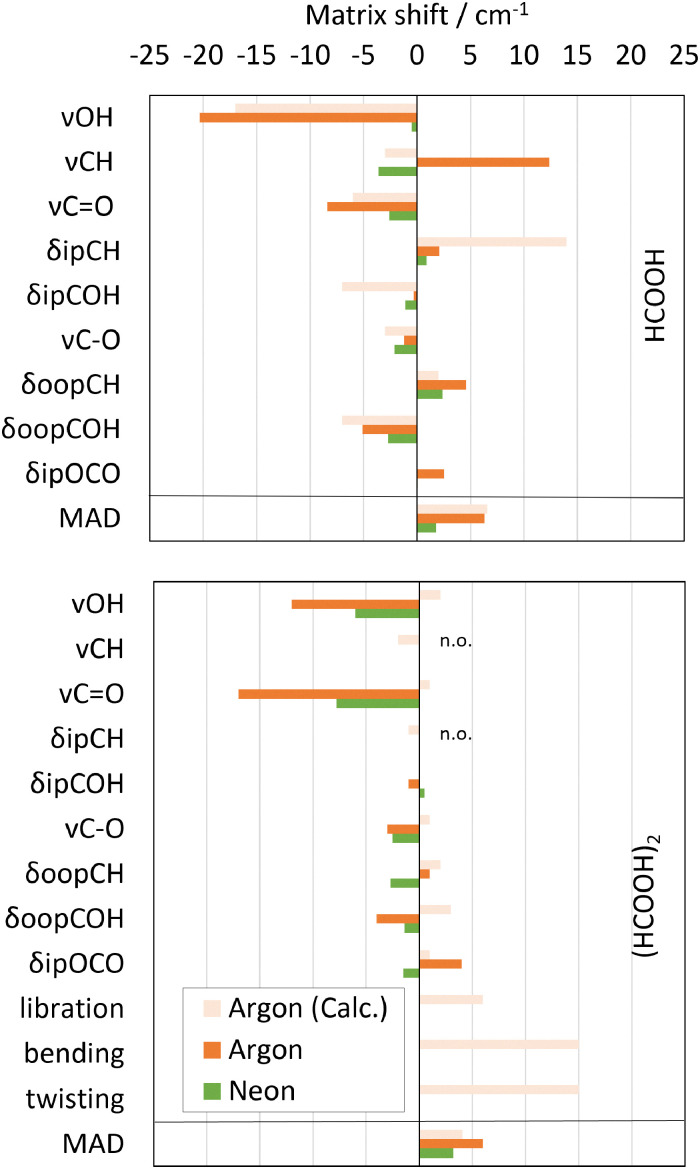
Matrix shifts for the *trans*-HCOOH monomer species (top) and *trans*-HCOOH cyclic dimer species in *C*_2h_ symmetry (bottom), as isolated in argon (orange) and neon (green) matrices in this work. The matrix shifts are calculated according to eqn (2) relative to the gas phase reference dataset by Nejad.^2^ For neon, some deviations are close to zero and are therefore barely visible in the plot. Calculated argon matrix shifts of the monomer are from Stepanian and Adamowicz^128^ and for the dimer from Ito (2019).^130^ MAD denotes the mean absolute deviation.

The matrix-induced frequency shifts of the (HCOOH)_2_ dimer vibrations show similar unsystematic behavior, as shown in Fig. 5. Moreover, we observe that the ^mat^*Δ*^*ν*OH/D^_Ar_ shift is much stronger in the monomer (−20 cm^−1^) than in the dimer (−12 cm^−1^). Then again, the ^mat^*Δ*^*ν*OH/D^_Ne_ shift is close to zero for the monomer, as stated above, but very pronounced for the dimer. Furthermore, the ^mat^*Δ*^*ν*C=O^_Ar_ and the ^mat^*Δ*^*ν*C=O^_Ne_ shifts are both stronger in the dimer compared to the monomer.

Fig. 5 includes the results of *ab initio* calculations that explicitly include the matrix environment by Ito^129,130^ for the dimer, and by Stepanian *et al.*^128^ for the monomer. As can be seen directly, these calculations are not yet predictive of matrix-induced frequency shifts. This holds true especially for the dimer. These discrepancies likely arise because the argon matrix in the experiment is less ordered than the models used in the calculations. As we noted elsewhere, a matrix experiment samples an ensemble of local crystalline environments within an overall amorphous solid rather than a single, well-defined host–guest structure.^164^

#### Dimer-formation frequency shifts

Another aspect that can be studied is the dimer-formation frequency shift. Taking the fundamental *ν*OH stretching vibration of the HCOOH monomer as a reference, the corresponding dimer-formation frequency shift is

3 ^dim^*Δ*^*ν*OH^ = *ν̃*^*ν*OH^(Dimer) − *ν̃*^*ν*OH^(Monomer).

In the gas phase, the shift amounts to ^dim^*Δ*^*ν*OH^_Gas_ = −486.5 cm^−1^. In an argon and neon matrix, the corresponding shifts are ^dim^*Δ*^*ν*OH^_Ar_ = −478.2 cm^−1^ and ^dim^*Δ*^*ν*OH^_Ne_ = −492.0 cm^−1^. These values show that matrix-induced frequency shifts of the monomer propagate directly into the observed dimer-formation frequency shifts. The calculated dimer-formation frequency shifts for the *ν*OH mode are −540.5 cm^−1^ using VCI, −473.3 cm^−1^ using VPT2, and −449.5 cm^−1^ at the harmonic level. All computational approaches, including the harmonic approximation, reproduce the qualitative redshift upon cluster formation. We present an extensive investigation of these dimer-formation frequency shifts in part II of our study, which also considers other conformers observed in the spectrum.^174^

### 3.5 Comparison of experiment with theory

In the present study, we investigate the extent to which *ab initio* calculations performed in the absence of an explicit matrix environment reproduce MI-FTIR spectra, using available gas phase data as a common reference for comparison. We list the observed fundamental bands and the most important resonances identified in our argon and neon MI-FTIR experiments of HCOOH, HCOOD, DCOOH, and DCOOD for the *trans*-monomers in Table 5. We also observe overtones and combination bands. For the assignment of resonances, as discussed earlier, it is essential to locate these bands for a full description of the vibrational states. We here list the bands we directly observe for the *trans*-formic acid monomer in Table 6. The fundamentals of the cyclic *trans*-formic acid dimer with *C*_2h_ symmetry are given in Table 7 for the (HCOOH)_2_ and (DCOOD)_2_ isotopocules. Predominantly *trans*-formic acid species are observed, as no IR pumping was applied, in contrast to the work of Pettersson *et al.*^61^ All tables report the vibrational frequencies obtained in this work using harmonic approximations, vibrational configuration interaction (VCI), and second-order vibrational perturbation theory (VPT2). Additional tables containing the complete set of observed and computed vibrational frequencies, together with their corresponding intensities and a comparison with selected literature data, are provided in Section S2 of the SI. Tables S1–S6 provide a detailed overview of the spectral assignments, enable the reader to trace the origin of the gas phase reference values used in Tables 5–7, and place the present results in the context of previous experimental and theoretical studies. In the following, we focus our discussion on deviations from the selected gas-phase reference values. The deviation of the theoretical results from the gas-phase reference values is defined as

4 ^dev^*Δ*^*ν*OH^_VCI_ = *ν̃*^*ν*OH^_VCI_(Monomer) − *ν̃*^*ν*OH^_Gas_(Monomer),

which quantifies the accuracy of the VCI calculation in reproducing the *νOH* stretching frequency of the monomer. Considering all vibrational modes collectively, we compute the mean absolute deviation (MAD) as a global measure of the accuracy of the respective computational method. In addition, the maximum deviation (MAX) identifies the single largest discrepancy and thus indicates which data point disproportionately influences the statistical measures.

Fundamental vibrations of the *trans*-formic acid monomer from experiment^a^ and calculation^b^

**Table:** 

HCOOH		Argon	*Δ*_Ar_	Neon	*Δ*_Ne_	Gas	VCI	*Δ*_VCI_	VPT2	*Δ*_VPT2_	HARM	*Δ*_HARM_
ν_1_(A′)	*ν*OH	3550.2	*−20.3*	3570.0	*−0.5*	3570.5	3571.5	*1.0*	3568.3	*−2.2*	3761.0	*190.5*
ν_2_(A′)	*ν*CH	2954.5	*12.4*	2938.5	*−3.6*	2942.1	2940.3	*−1.8*	2942.6	*0.5*	3091.3	*149.2*
ν_3_(A′)	*ν*C=O	1768.4	*−8.4*	1774.2	*−2.6*	1776.8	1778.4	*1.6*	1778.8	*2.0*	1813.9	*37.1*
ν_4_(A′)	*δ*_ip_CH	1381.2	*2.1*	1380.0	*0.9*	1379.1	1379.1	*0.0*	1378.2	*−0.9*	1409.2	*30.1*
ν_5_/2ν_9_(A′)	*δ*_ip_COH	1305.9	*−0.3*	1305.1	*−1.1*	1306.2	1310.5	*4.3*	1302.0	*−4.2*	1317.5	*11.3*
ν_6_(A′)	*ν*C–O	1103.7	*−1.2*	1102.8	*−2.1*	1104.9	1106.2	*1.3*	1105.6	*0.7*	1138.2	*33.3*
ν_8_(A″)	*δ*_oop_CH	1038.1	*4.6*	1035.9	*2.4*	1033.5	1032.7	*−0.8*	1033.0	*−0.5*	1055.4	*21.9*
ν_9_(A″)	*δ*_oop_COH	635.6	*−5.1*	638.0	*−2.7*	640.7	641.6	*0.9*	636.3	*−4.4*	672.4	*31.7*
ν_7_(A′)	*δ*_ip_OCO	628.7	*2.5*	626.3	*0.1*	626.2	626.3	*0.1*	627.3	*1.1*	631.9	*5.7*
MAD			*6.3*		*1.8*			*1.3*		*1.8*		*56.8*
MAX			*20.3*		*3.6*			*4.3*		*4.4*		*190.5*

a MI-FTIR data in argon or neon from this work (except ν_9_ of HCOOD from ref. 68, and of DCOOD from ref. 30 and 36). Split bands in argon spectra were averaged. Gas phase reference data, as compiled by Nejad,^2^ are from high-resolution studies where available (*trans*-HCOOH,^40–44,127,128^*trans*-DCOOH,^46–50,129,130^*trans*-HCOOD,^51–54,127,129,131^ and *trans*-DCOOD^12,37,54–57^) and otherwise from Raman-jet spectra.^98,100^ Resonant overtones and combination bands are denoted, where applicable, following the “/” symbol, and the corresponding resonance partners are listed in Table 6. The matrix shifts in argon (*Δ*_Ar_) and neon (*Δ*_Ne_) are calculated according to eqn (2) with reference to the gas phase.

b Harmonic frequencies (HARM), vibrational perturbation theory of 2nd order (VPT2), and vibrational configuration interaction (VCI) as calculated in this work. Details on the potential energy surface are given in Table 1. The computational deviations (*Δ*_VCI_, *Δ*_VPT2_, *Δ*_HARM_) are calculated according to eqn (1) with reference to the gas phase.

**Table:** 

DCOOH		Argon	*Δ*_Ar_	Neon	*Δ*_Ne_	Gas	VCI	*Δ*_VCI_	VPT2	*Δ*_VPT2_	HARM	*Δ*_HARM_
ν_1_(A')	*ν*OH	3551.5	*−14.5*	3570.4	*4.4*	3566	3574.0	*8.0*	3568.8	*2.8*	3760.9	*194.9*
ν_2_(A′)	*ν*CD	2225.3	*5.6*	2215.9	*−3.8*	2219.7	2219.6	*−0.1*	2227.3	*7.6*	2299.3	*79.6*
ν_3_/2ν_8_(A′)	*ν*C=O	1761.3	*−1.6*	1763.1	*0.2*	1762.9	1764.2	*1.3*	1766.9	*4.0*	1782.4	*19.5*
ν_4_/2ν_9_(A′)	*δ*_ip_COH	1200.1	*−5.9*	1203.0	*−3.0*	1206	1205.6	*−0.4*	1243.9	*37.9*	1312.4	*106.4*
ν_5_(A′)	*ν*C–O	1141.9	*−0.4*	1141.1	*−1.2*	1142.3	1143.1	*0.8*	1143.5	*1.2*	1175.2	*32.9*
ν_6_(A′)	*δ*_ip_CD	975.2	*4.3*	971.6	*0.7*	970.9	970.7	*−0.2*	974.3	*3.4*	989.8	*18.9*
ν_8_(A″)	*δ*_oop_CD	874.9	*1.5*	875.1	*1.7*	873.4	872.8	*−0.6*	876.5	*3.1*	888.1	*14.7*
ν_9_(A″)	*δ*_oop_COH	626.2	*−5.3*	628.7	*−2.8*	631.5	632.8	*1.3*	627.3	*−4.2*	662.3	*30.8*
ν_7_(A′)	*δ*_ip_OCO	622.9	*2.3*	620.6	*0.0*	620.6	620.5	*−0.1*	621.5	*0.9*	626.0	*5.4*
MAD			*4.6*		*2.0*			*1.4*		*7.2*		*55.9*
MAX			*14.5*		*4.4*			*8.0*		*37.9*		*194.9*

**Table:** 

HCOOD		Argon	*Δ*_Ar_	Neon	*Δ*_Ne_	Gas	VCI	*Δ*_VCI_	VPT2	*Δ*_VPT2_	HARM	*Δ*_HARM_
ν_1_/ν_3_ + ν_5_(A′)	*ν*CH	2941.4	*3.4*	2936.1	*−1.9*	2938	2939.4	*1.4*	2941.0	*3.0*	3091.8	*153.8*
ν_2_(A′)	*ν*OD	2619.2	*−12.4*	2632.1	*0.5*	2631.6	2632.4	*0.8*	2630.2	*−1.4*	2734.6	*103.0*
ν_3_(A′)	*ν*C=O	1765.9	*−6.2*	1771.3	*−0.8*	1772.1	1775.5	*3.4*	1775.2	*3.1*	1807.5	*35.4*
ν_4_(A′)	*δ*_ip_CH	1371.1	*4.6*	1368.2	*1.7*	1366.5	1366.1	*−0.4*	1366.8	*0.3*	1399.4	*32.9*
ν_5_(A′)	*ν*C–O	1181.4	*4.3*	1177.2	*0.1*	1177.1	1178.5	*1.4*	1178.7	*1.6*	1207.1	*30.0*
ν_8_(A″)	*δ*_oop_CH	1043.9	*12.9*	1037.5	*6.5*	1031	1030.8	*−0.2*	1031.2	*0.2*	1053.1	*22.1*
ν_6_/2ν_9_(A′)	*δ*_ip_COD	970.7	*−2.2*	971.2	*−1.7*	972.9	971.5	*−1.4*	972.6	*−0.3*	1009.8	*36.9*
ν_7_(A′)	*δ*_ip_OCO	559.5	*1.2*	557.7	*−0.6*	558.3	558.2	*−0.1*	558.6	*0.3*	564.7	*6.4*
ν_9_(A″)	*δ*_oop_COD	506.0	*−2.1*	506.9	*−1.2*	508.1	506.2	*−1.9*	506.9	*−1.2*	528.3	*20.2*
MAD			*5.5*		*1.7*			*1.2*		*1.3*		*49.0*
MAX			*12.9*		*6.5*			*3.4*		*3.1*		*153.8*

**Table:** 

DCOOD		Argon	*Δ*_Ar_	Neon	*Δ*_Ne_	Gas	VCI	*Δ*_VCI_	VPT2	*Δ*_VPT2_	HARM	*Δ*_HARM_
ν_1_(A′)	*ν*OD	2619.5	*−12.4*	2632.2	*0.3*	2631.9	2632.8	*0.9*	2631.1	*−0.8*	2735.1	*103.2*
ν_2_/ν_4_ + ν_5_(A′)	*ν*CD	2238.1	*6.1*	2229.1	*−2.9*	2232	2231.0	*−1.0*	2239.0	*7.0*	2298.1	*66.1*
ν_3_/2ν_8_(A′)	*ν*C=O	1759.5	*−0.5*	1761.9	*1.9*	1760	1762.3	*2.3*	1735.1	*−24.9*	1778.2	*18.2*
ν_4_(A′)	*ν*C–O	1175.2	*4.4*	1170.8	*0.0*	1170.8	1172.2	*1.4*	1172.0	*1.2*	1199.9	*29.1*
ν_5_(A′)	*δ*_ip_COD	1039.4	*−2.6*	1039.3	*−2.7*	1042	1039.1	*−2.9*	1039.4	*−2.6*	1058.3	*16.3*
ν_6_/2ν_9_(A′)	*δ*_ip_CD	945.8	*0.8*	945.0	*0.0*	945	946.2	*1.2*	947.1	*2.1*	966.8	*21.8*
ν_8_(A″)	*δ*_oop_CD	874.9	*1.7*	875.0	*1.8*	873.2	873.0	*−0.2*	876.7	*3.5*	888.0	*14.8*
ν_7_(A′)	*δ*_ip_OCO	555.3	*0.9*	554.8	*0.4*	554.4	554.5	*0.1*	554.7	*0.3*	560.7	*6.3*
ν_9_(A″)	*δ*_oop_COD	489.8	*−2.4*	490.4	*−1.8*	492.2	490.4	*−1.8*	491.5	*−0.7*	511.0	*18.8*
MAD			*3.5*		*1.3*			*1.3*		*4.8*		*32.7*
MAX			*12.4*		*2.9*			*2.9*		*24.9*		*103.2*

Selected overtones and combination bands of the *trans*-formic acid monomer from experiment^a^ and calculation^b^

**Table:** 

HCOOH	Argon	*Δ*_Ar_	Neon	*Δ*_Ne_	Gas	VCI	*Δ*_VCI_	VPT2	*Δ*_VPT2_	HARM	*Δ*_HARM_
2ν_3_(A′)	3516.5	*−17.5*	3529.6	*−4.4*	3534	3537.9	*3.9*	3538.9	*4.9*	3627.8	*93.8*
ν_3_ + ν_6_(A′)	2866.2	*−10.4*	2872.0	*−4.6*	2876.6	2879.6	*3.0*	2879.2	*2.6*	2952.1	*75.5*
ν_6_ + 2ν_9_/ν_4_ + ν_5_(A′)	2398.1	*−2.1*	2397.0	*−3.2*	2400.2	2406.5	*6.3*	2396.1	*−4.1*	2483.0	*82.8*
ν_4_ + ν_5_/ν_6_ + 2ν_9_(A′)	2328.9	*−7.1*	2329.5	*−6.5*	2336.0	2328.6	*−7.4*	2309.5	*−26.5*	2455.7	*119.7*
2ν_6_(A′)	2195.4	*−0.9*	2193.3	*−3.0*	2196.3	2201.3	*5.0*	2199.6	*3.3*	2276.4	*80.1*
ν_5_/2ν_9_(A′)	1215.9	*−4.9*	1217.7	*−3.1*	1220.8	1230.6	*9.8*	1213.6	*−7.2*	1344.8	*124.0*

a Matrix isolation IR data in argon or neon from this work. Gas phase reference data, as compiled by Nejad,^2^ are from high-resolution studies where available (*trans*-HCOOH,^40–44,127,128^*trans*-DCOOH,^46–50,129,130^*trans*-HCOOD,^51–54,127,129,131^ and *trans*-DCOOD^12,37,54–57^) and otherwise from Raman-jet spectra.^98,100^ The matrix shifts in argon (*Δ*_Ar_) and neon (*Δ*_Ne_) are calculated according to eqn (2) with reference to the gas phase.

b Harmonic frequencies (HARM), vibrational perturbation theory of 2nd order (VPT2), and vibrational configuration interaction (VCI) as calculated in this work. Details on the potential energy surface are given in Table 1. The computational deviations (*Δ*_VCI_, *Δ*_VPT2_, *Δ*_HARM_) are calculated according to eqn (1) with reference to the gas phase.

**Table:** 

DCOOH	Argon	*Δ*_Ar_	Neon	*Δ*_Ne_	Gas	VCI	*Δ*_VCI_	VPT2	*Δ*_VPT2_	HARM	*Δ*_HARM_
2ν_3_(A′)	3461.8	*n.o.*	3470.1	*n.o.*	n.o.	3475.0	*n.o.*	3482.3	*n.o.*	3564.8	*n.o.*
ν_3_ + ν_5_/ν_5_ + 2ν_8_(A′)	2896.1	*−1.9*	2897.6	*−0.4*	2898	2900.3	*−2.9*	2903.2	*5.2*	2957.6	*59.6*
ν_5_ + 2ν_8_/ν_3_ + ν_5_(A′)	2857.7	*−2.3*	2859.6	*−0.4*	2860	2861.0	*−3.6*	2864.6	*4.6*	2951.4	*91.4*
ν_5_ + ν_6_(A′)	2106.2	*3.2*	2102.0	*−1.0*	2103	2103.0	*−4.3*	2107.3	*4.3*	2165.0	*61.9*
2ν_6_(A′)	1943.7	*6.7*	1938.5	*1.5*	1937	1936.7	*−6.1*	1942.8	*5.8*	1979.6	*42.6*
ν_4_ + ν_7_(A′)	1918.2	*−0.8*	1917.2	*−1.8*	1919	1925.0	*−0.7*	1925.7	*6.7*	1938.4	*19.4*
ν_3_/2ν_8_(A′)	1723.2	*−2.7*	1726.2	*0.3*	1725.9	1725.8	*−3.0*	1728.8	*2.9*	1776.2	*50.3*
ν_4_/2ν_9_(A′)	n.o.	*n.o.*	n.o.	*n.o.*	1299	1298.4	*n.o.*	1294.3	*n.o.*	1251.9	*n.o.*

**Table:** 

HCOOD	Argon	*Δ*_Ar_	Neon	*Δ*_Ne_	Gas	VCI	*Δ*_VCI_	VPT2	*Δ*_VPT2_	HARM	*Δ*_HARM_
2ν_3_(A′)	3512.9	*−16.1*	3524.7	*−4.3*	3529.0	3532.9	*3.9*	3532.6	*3.6*	3615.0	*86.0*
ν_1_/ν_3_ + ν_5_(A′)	2961.6	*7.6*	2952.4	*−1.6*	2954.0	2956.8	*2.8*	2957.0	*3.0*	3014.6	*60.6*
ν_5_ + ν_6_ + ν_7_(A′)	2677.8	*−14.2*	2696.5	*4.5*	2692.0	2693.8	*1.8*	2696.1	*4.1*	2781.6	*89.6*
ν_5_ + 2ν_9_/ν_5_ + ν_6_(A′)	2181.1	*2.3*	2177.4	*−1.4*	2178.8	2179.2	*0.4*	2181.6	*2.8*	2263.7	*84.9*
ν_5_ + ν_6_/ν_5_ + 2ν_9_(A′)	2144.3	*1.9*	2140.2	*−2.2*	2142.4	2142.2	*−0.2*	2144.2	*1.8*	2216.9	*74.5*
ν_6_/2ν_9_(A′)	1009.3	*−2.4*	1009.7	*−2.0*	1011.7	1009.5	*−2.2*	1009.5	*−2.2*	1056.6	*44.9*

**Table:** 

DCOOD	Argon	*Δ*_Ar_	Neon	*Δ*_Ne_	Gas	VCI	*Δ*_VCI_	VPT2	*Δ*_VPT2_	HARM	*Δ*_HARM_
2ν_3_(A′)	3459.5	*n.o.*	3466.9	*n.o.*	n.o.	3472.0	*n.o.*	3456.0	*n.o.*	3556.4	*n.o.*
ν_3_ + ν_4_/ν_4_ + 2ν_8_(A′)	2929.6	*3.6*	2927.4	*1.4*	2926.0	2929.0	*3.0*	2924.1	*−1.9*	2978.1	*52.1*
ν_4_ + 2ν_8_/ν_3_ + ν_4_(A′)	2892.5	*4.5*	2889.5	*1.5*	2888.0	2890.3	*2.3*	2899.2	*11.2*	2975.9	*87.9*
ν_3_ + ν_6_/ν_6_ + 2ν_8_(A′)	n.o.	*n.o.*	2704.5	*0.5*	2704.0	2706.4	*2.4*	2702.9	*−1.1*	2745.0	*41.0*
ν_6_ + 2ν_8_/ν_3_ + ν_6_(A′)	2661.1	*−6.9*	2667.9	*−0.1*	2668.0	2669.8	*1.8*	2677.2	*9.2*	2742.8	*74.8*
ν_4_ + ν_5_/ν_2_(A′)	2198.7	*4.7*	2193.0	*−1.0*	2194.0	2195.2	*1.2*	2198.4	*4.4*	2258.2	*64.2*
ν_4_ + ν_6_(A′)	2112.4	*4.4*	2107.3	*−0.7*	2108.0	2109.6	*1.6*	2116.7	*8.7*	2166.7	*58.7*
2ν_5_(A′)	2072.5	*−0.5*	2072.8	*−0.2*	2073.0	2072.6	*−0.4*	2071.8	*−1.2*	2116.6	*43.6*
ν_3_/2ν_8_(A′)	1720.8	*−4.3*	1725.5	*0.4*	1725.1	1725.5	*0.4*	1711.9	*−13.2*	1776.0	*50.9*
ν_6_/2ν_9_(A′)	962.4	*−2.6*	962.9	*−2.1*	965.0	962.9	*−2.1*	963.3	*−1.7*	1022.0	*57.0*

Fundamental vibrations of the *trans*-formic acid cyclic dimer from experiment^a^ and calculation^b^

**Table:** 

(HCOOH)_2_		Argon	*Δ*_Ar_	Neon	*Δ*_Ne_	Gas	VCI	*Δ*_VCI_	VPT2	*Δ*_VPT2_	HARM	*Δ*_HARM_
ν_1_(Ag)	*ν*OH						2943		2949		3210	
ν_2_(Ag)	*ν*CH					*2949*	2960		2914		3103	
ν_3_(Ag)	*ν*C=O					*1670*	1671	1	1671	1	1718	48
ν_4_(Ag)	*δ*_ip_COH					1430	1432	2	1429	−1	1482	52
ν_5_(Ag)	*δ*_ip_CH					1375	1375	0	1363	−12	1410	35
ν_6_(Ag)	*ν*C−O					1224	1221	−3	1226	2	1254	30
ν_7_(Ag)	*δ*_ip_OCO					681	680	−1	679	−2	686	5
ν_8_(Ag)	Stretching					194	196	2	193	−1	209	15
ν_9_(Ag)	Bending, ip					161	159	−2	153	−8	168	7
ν_10_(Bg)	*δ*_oop_CH					1058	1055	−3	1052	−6	1084	26
ν_11_(Bg)	*δ*_oop_COH					911	905	−6	904	−7	957	46
ν_12_(Bg)	Libration, ip					242	240	−2	229	−13	259	17
ν_13_(Au)	*δ*_oop_CH	1070	1	1066	−3	1069	1056	−13	1058	−11	1098	29
ν_14_(Au)	*δ*_oop_COH	940	−4	943	−1	944	936	−8	944	0	983	39
ν_15_(Au)	Bending, oop					168	166	−2	159	−9	180	12
ν_16_(Au)	Twisting					69	67	−2	68	−1	69	0
ν_17_(Bu)	*ν*OH	3072		3078		*3084*	3031		3095		3310	
ν_18_(Bu)	*ν*CH	*		*		*2939*	2955		2907		3099	
ν_19_(Bu)	*ν*C=O	1729	−17	1738	−8	*1746*	1742	−4	1741	−5	1782	36
ν_20_(Bu)	*δ*_ip_CH	*		*		1407	1404	−3	1404	−3	1455	48
ν_21_(Bu)	*δ*_ip_COH	1371	−1	1373	1	1372	1370	−2	1362	−10	1406	34
ν_22_(Bu)	*ν*C−O	1227	−3	1228	−3	1230	1230	0	1229	−1	1258	28
ν_23_(Bu)	*δ*_ip_ OCO	712	4	707	−2	708	708	0	705	−3	714	6
ν_24_(Bu)	Libration, oop					264	266	2	262	−2	274	10
MAD			6		3			4		5		26
MAX			17		8			13		11		48

a Matrix isolation FTIR spectroscopy in the range of 4000 to 500 cm^−1^ was performed in this work. The values are obtained from a 1 : 250 mixture of the respective formic acid isotopocule in argon or neon. Observed but unassigned bands, due to the high signal-to-noise ratio, are indicated with an asterisk (*). IR and Raman reference data of jet-cooled formic acid up to 1500 cm^−1^ were taken from Nejad.^2^ Gas phase references above 1500 cm^−1^ taken from literature are highlighted in *italics*: (HCOOH)_2_ ν_17_, ν_18_, ν_19_ by Georges *et al.*;^88^ (HCOOH)_2_/(DCOOD)_2_ ν_2_ and ν_3_ by Bertie *et al.*;^37^ (DCOOD)_2_ ν_16_ by Marechal *et al.*^39^ and ν_19_ by Gutberlet *et al.*.^175^ The matrix shifts in argon (*Δ*_Ar_) and neon (*Δ*_Ne_) are calculated according to eqn (2) with reference to the gas phase. The experimental gas phase band centers of OH and OD stretch are tentative assignments.

b Harmonic frequencies (HARM), vibrational perturbation theory of 2nd order (VPT2), and vibrational configuration interaction (VCI) as calculated in this work. Details on the potential energy surface are given in Table 1. The computational deviations (*Δ*_VCI_, *Δ*_VPT2_, *Δ*_HARM_) are calculated according to eqn (1) with reference to the gas phase. The MAD and MAX values reported here include all IR active fundamentals (Au, Bu) with frequencies in the range of 2000–500 cm^−1^.

**Table:** 

(DCOOD)_2_		Argon	*Δ*_Ar_	Neon	*Δ*_Ne_	Gas	VCI	*Δ*_VCI_	VPT2	*Δ*_VPT2_	HARM	*Δ*_HARM_
ν_1_(Ag)	*ν*OD						2241		2236		2348	
ν_2_(Ag)	*ν*CD					*2211*	2211		2208		2302	
ν_3_(Ag)	*ν*C=O					*1648*	1637	−11	1641	−7	1678	30
ν_4_(Ag)	*ν*C−O					1258	1257	−1	1254	−4	1282	24
ν_5_(Ag)	*δ*_ip_COD					1092	1093	1	1089	−3	1123	31
ν_6_(Ag)	*δ*_ip_CD					992	992	0	989	−3	1011	19
ν_7_(Ag)	*δ*_ip_OCO					623	622	−1	620	−3	629	6
ν_8_(Ag)	Stretching					192	193	1	192	0	205	13
ν_9_(Ag)	Bending, ip					157	156	−1	156	−1	164	7
ν_10_(Bg)	*δ*_oop_CD					893	892	−1	893	0	907	14
ν_11_(Bg)	*δ*_oop_COD					669	670	1	656	−13	702	33
ν_12_(Bg)	Libration, ip					210	207	−3	208	−2	223	13
ν_13_(Au)	*δ*_oop_CD	*		*			891		893		907	
ν_14_(Au)	*δ*_oop_COD	710	−2	710	−2	712	707	−5	711	−1	740	28
ν_15_(Au)	Bending, oop						138		132		150	
ν_16_(Au)	Twisting						69		65		70	
ν_17_(Bu)	*ν*OD	2260		2260		*2270*	2269		2273		2412	
ν_18_(Bu)	*ν*CD	2208		2219			2214		2204		2299	
ν_19_(Bu)	*ν*C=O	1710	−7	1715	−2	*1717*	1720	3	1716	−1	1756	39
ν_20_(Bu)	*ν*C−O	1256	−1	1256	−1	1257	1264	7	1255	−2	1288	31
ν_21_(Bu)	*δ*_ip_COD	1073	1	1074	2	1072	1072	0	1069	−3	1101	29
ν_22_(Bu)	*δ*_ip_CD	987	0	988	1	987	985	−2	982	−5	1006	19
ν_23_(Bu)	*δ*_ip_OCO	660	4	655	−2	656	656	0	653	−3	665	9
ν_24_(Bu)	Libration, oop					250	250		248		262	
MAD			3		2			3		3		26
MAX			7		2			7		5		39

#### Spectroscopic accuracy

Spectroscopic accuracy is reached when the discrepancy between computed and experimentally observed transitions is smaller than the spacing between the observed transitions themselves. In gas phase infrared spectra, the large number of rovibrational transitions makes this criterion extremely demanding. In MI-FTIR spectra, however, rotational structure is quenched, which simplifies this comparison considerably. In this case, we consider reaching spectroscopic accuracy effectively when the error of the anharmonic calculation relative to the gas phase reference is on the same order of magnitude as the matrix-induced frequency shifts. In a series of studies,^163–165,172,176,177^ we have shown that VCI calculations can achieve spectroscopic accuracy relative to MI-FTIR spectra.

As reflected by the mean absolute deviations (MAD) listed in Table 5 for the four monomer isotopocules, the calculated frequencies deviate from gas phase reference values by less than the typical matrix-induced frequency shifts for argon and neon matrices. Compared to argon matrices, the *ab initio* results approach the gas phase reference better, and compared to neon matrices, both the calculated and the matrix-isolation measurements agree very well with the gas phase. In the case of the monomer, we can confidently conclude that the calculations presented here achieve spectroscopic accuracy in MI-FTIR spectroscopy.

Regarding the overtones and combination (*cf.*Table 6), looking at their associated harmonic values, it becomes immediately evident that the calculation of accurate anharmonic vibrational transitions is crucial. While the harmonic approximation highly overestimates the band position with up to 124 cm^−1^. The respective values obtained within our VCI/VPT2 anharmonic framework typically yield deviations of a few cm^−1^ relative to the gas phase, again achieving spectroscopic accuracy with deviations similar to those of argon and neon matrix-isolation spectra. In almost all cases, VPT2 frequencies show higher deviations than VCI frequencies. This can be explained by the fact that VCI is not restricted to quartic or semi-quartic force fields, thereby leading to a better description of higher-energy states, *e.g.*, overtones and combinations.^178^ The VCI space, as described in the theory section (*cf.* Section 1), enables an accurate representation of resonant states, further improving the accuracy of band positions with respect to gas-phase values. In combination with our extensive experimental dataset, comprising measurements of multiple isotopocules across a range of concentrations, this allows us to directly compare overtone and combination band shifts. This approach ultimately yields agreement between theory and experiment for overtones and combination bands that is comparable in accuracy to that for the fundamentals. Further discussion for the assignment is provided in the SI, Section S1.

Considering the dimer, the situation is more involved. First, we have to distinguish between Raman- and IR-active bands. As we employ matrix-isolation IR spectroscopy here, we calculate the MAD for the dimer (*cf.*Table 7) focusing only on IR-active bands. Next, we need to decide on a reliable reference dataset for the dimer. The Raman and IR dataset by Nejad^2^ focuses on the region below 1500 cm^−1^. This is because investigations of the stretching vibrations of the dimer in gas phase are tedious, due to overlapping rovibrational features with the monomer bands, as well as various resonances. For the vibrational band centers above 1500 cm^−1^, we have to rely on different sources,^39,88,175^ which introduces an unknown error margin for this reference. Furthermore, some bands in this region still remain unassigned in the gas phase to the best of our knowledge. To avoid these unkown error margins and unassigned bands, we include only the IR-active bands between 2000 and 500 cm^−1^ to evaluate the MAD and MAX. Ultimately, we expected that the actual matrix-induced frequency shifts would be higher and that the computational accuracy would be better than Table 7 suggests. In other words, for the dimer as well, we are confident that the presented computational approach achieves spectroscopic accuracy relative to MI-FTIR spectroscopy.

#### Accuracy of different computational approaches

**Fig. 6:**
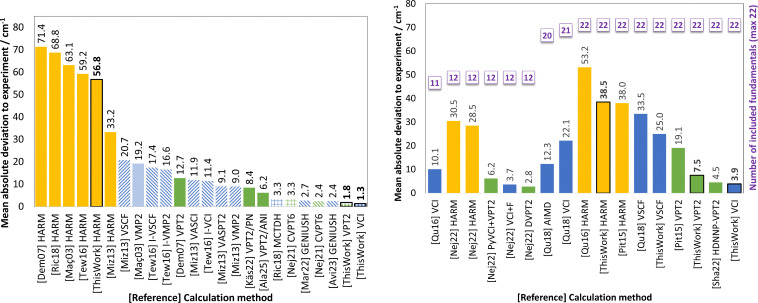
Accuracy of computational spectroscopy approaches illustrated for the *trans*-HCOOH monomer (left) and the (*trans*-HCOOH)_2_ cyclic dimer (right). Bars represent the mean absolute deviation (MAD) of the computed vibrational fundamentals compared to the gas phase experimental reference dataset by Nejad.^2^ Te MAD is based on the the deviations as calculated according to eqn (1). Colors indicate the general category of vibrational frequency calculation: yellow for harmonic (HARM) calculations, which differ only in the level of electronic structure theory, blue for anharmonic calculations (*e.g.*, VSCF, VMP2, VCI, VASPT, VASCI, AIMD, MCTDH, GENUISH); and green for anharmonic approaches from vibrational perturbation theory (VPT). In the case of the monomer, bar pattern fills denote the potential energy surface (PES) used: right-to-left diagonal lines for the Mizukami2013 PES and a chessboard pattern for the PES developed in this work, both being local PESs for *trans*-formic acid in rectilinear coordinates only; left-to-right diagonal lines for the Tew2016 PES and a gridded pattern for the Richter2018 PES, both being semi-global PESs including the *cis*-formic acid and employing curvilinear coordinates. Except for the two machine-learned PESs (PN and ANI), all PESs considered are based purely on *ab initio* methods. For the dimer, the MAD was calculated using varying numbers of vibrational transitions (indicated in purple), since not all references reported all 24 fundamental transitions. The absolute frequencies included in the analysis are listed in the SI, Section S3. References are indicated in brackets using a three-letter abbreviation of the first author's surname followed by the last two digits of the publication year (format: [AutYY]). Our results are highlighted with bold bars and black outlines.

We now place theoretical predictions of the formic acid spectrum in the context of MI-FTIR spectroscopy and examine the level of theory required to achieve the spectroscopic accuracy defined above. Following the recent review by Kelemen and Lubner,^105^ we compare vibrational frequency calculations for the *trans*-formic acid conformer of the HCOOH isotopocule and its cyclic dimer. Our analysis includes our current results and literature data published after 2000. The comparison is summarized in Fig. 6, where the mean absolute deviation (MAD) relative to the experiment is used to quantify the performance of the different computational approaches. For the monomer, we include all vibrational fundamentals in the analysis. For the dimer, not all vibrations were considered in the literature, so we also indicate the number of fundamentals included in the analysis. Note that the here presented evaluation is focused on the fundamentals and it can be expected that the deviations behave differently when including higher overtones, especially of large amplitude motions such as the internal torsion (*c.f*. Table 6).

#### Formic acid monomer

Harmonic frequency calculations (yellow bars) show the largest deviations from experiment, with MAD values reaching up to 70 cm^−1^. These errors are far larger than the typical matrix-induced frequency shifts observed in argon (MAD = 4–6 cm^−1^) and neon (MAD = 1–2 cm^−1^). The comparatively favorable result reported in ref. 111 most likely arises from fortuitous error compensation, as the calculation combines CCSD(T)-F12 with a double-zeta basis set. In contrast, anharmonic calculations (blue and green bars) consistently outperform the harmonic approximation, with MAD values typically below 20 cm^−1^. Since *trans*-formic acid is a semi-rigid molecule with only one internal torsion and a relatively high barrier, many methods already yield reasonably accurate results without extensive methodological refinement. The remaining discrepancies mainly reflect the quality of the underlying potential energy surface (PES) and the coordinate representation used in the vibrational treatment.

The approaches by Mizukami and Tew,^111^ based on a local PES for *trans*-formic acid, illustrate a clear methodological progression from the VSCF approach (MAD = 20.7 cm^−1^) to correlation-corrected methods such as second-order vibrational Møller–Plesset perturbation theory (VMP2), which reduces the MAD to 9.0 cm^−1^ (*cf.*Fig. 6, blue bars with left-to-right diagonal lines). Although this already represents a substantial improvement over the harmonic approximation, the remaining deviation is still larger than typical matrix-induced frequency shifts and may therefore complicate assignments in certain regions of the matrix-isolation IR spectrum.

Semi-global PESs that include both the *trans*- and *cis*-conformers have also been developed. The PES by Tew from 2016,^112^ formulated in curvilinear coordinates connecting the two conformers, shows a progression from I-VSCF (MAD = 19.2 cm^−1^) to I-VCI (MAD = 11.4 cm^−1^), where “I” denotes the use of internal (curvilinear) coordinates (*cf.*Fig. 6, blue bars with right-to-left diagonal lines). Even higher accuracy is obtained when the same PES is treated with the GENIUSH algorithm, yielding a MAD of 2.4 cm^−1^.^114,115^ Similarly, the semi-global PES developed by Richter in 2018^113^ yields a MAD of 3.3 cm^−1^ when combined with the multiconfigurational time-dependent Hartree (MCTDH) method, which in its exact limit is formally comparable to the VCI approach.

In contrast to these studies, our present calculations are based on a newly generated local PES for *trans*-formic acid expressed in rectilinear coordinates (*cf.*Table 1). Despite this local expansion of the PES, our VCI treatment achieves the best accuracy among the approaches considered here, with a MAD of 1.3 cm^−1^. At this level, the deviations from gas phase reference data become comparable to, or smaller than, the matrix-induced frequency shifts observed in argon (MAD = 4–6 cm^−1^) and neon (MAD = 1–2 cm^−1^). Consequently, the calculations reach spectroscopic accuracy for matrix-isolation FTIR spectroscopy. Specifically, (a) the remaining errors of the calculations are smaller than the frequency perturbations introduced by the matrix environment and (b) they are smaller than the spacing between individual spectral lines. This level of accuracy is therefore sufficient for reliable spectral assignments without explicitly including matrix effects.

We now turn to approaches based on vibrational perturbation theory. Notably, the early VPT2 calculations by Demaison,^110^ based on an MP2 quartic force field, already yield reasonably good agreement with experiment, with a MAD of 12.7 cm^−1^. More recent work has explored machine-learning approaches to generate the quadratic, cubic, and quartic force constants required for VPT2 through numerical differentiation. Comparable accuracy was reported by Käser *et al.*^123^ using PhysNet (MAD = 8.4 cm^−1^) and by Alavi *et al.*^124^ using ANI (MAD = 6.2 cm^−1^). These machine-learned force fields were optimized (or “transfer-learned”) toward CCSD(T) *ab initio* energies. The improved performance of the VPT2 calculations by Käser *et al.*^123^ and Alavi *et al.*,^124^ relative to the earlier study by Demaison,^110^ is therefore most plausibly attributable to the higher accuracy of CCSD(T) compared with MP2.

In the present work, applying VPT2 to a semi-quartic force field derived from our n-mode PES yields a MAD of 1.8 cm^−1^. This reflects the accuracy of the underlying PES (*cf.*Table 1), as the quartic force constants are obtained directly from its polynomial representation. The comparison, therefore, illustrates how systematic improvements in the force field directly translate into better VPT2 predictions. Within this context, the sixth-order canonical Van Vleck perturbation theory (CVPT6) used by Nejad and Siebert^103^ is also noteworthy. Using the Richter2018 PES, it yields a MAD of 3.3 cm^−1^, which decreases to 2.4 cm^−1^ with the Tew2016 PES, matching the best results obtained with GENIUSH. That said, from vibrational perturbation theory, it is possible to achieve spectroscopic accuracy in MI-FTIR spectroscopy.

The close agreement between these perturbative and variational approaches, both in the literature and in our own comparison of VPT2 and VCI on the present PES, likely reflects the semi-rigid nature of the *trans*-formic acid molecule. Ultimately, the superior performance of the present calculations can be traced to the construction of the underlying PES (see Table 1). In contrast to the Tew2016 and Richter2018 PESs, our surface includes electron correlation consistently at the CCSD(T) level, with correlation contributions extrapolated using Goodson's continued-fraction scheme.^144^ This approach is known to improve accuracy, particularly for high-frequency stretching vibrations.^151,179,180^

#### Formic acid dimer

Due to its larger size, the formic acid dimer poses a greater computational challenge than the monomer. The level of theory used to construct the PES must be limited, and certain approximations in the vibrational treatment are unavoidable. Despite these limitations, progress has been made over the past decade in modern computational studies, as shown in Fig. 6. Although the comparison is somewhat involved, as not all vibrational transitions have been reported across the studies, the overall trend indicates a steady improvement in computational accuracy for the dimer. The corresponding deviations, as depicted in Fig. 6, confirm a known trend of improvement from harmonic to anharmonic approaches. Harmonic calculations by Pitsevich *et al.*^181^ and Qu and Bowman^118^ yielded MADs between 38 and 53 cm^−1^. We observe a MAD of 38.5 cm^−1^ for our harmonic calculations.

The first high-level PES of the formic acid dimer, intended for anharmonic frequency calculations, was developed by Qu and Bowman (2016)^118^ at the CCSD(T) level of theory. This so-called Bowman2016 PES served as the foundation for anharmonic studies employing VSCF, VCI, and AIMD methods.^119,120^ A progressive improvement for the calculated vibrational frequencies was observed, reducing MAD from about 53 cm^−1^ for harmonic estimates to around 10 cm^−1^ for anharmonic treatments (considering only a fraction of the fundamental vibrations). In 2022, Nejad^2^ revisited the Bowman2016 PES with a series of different vibrational approaches, including DVPT2, VCI + F, and PyVCI + VPT2, achieving MAD as low as 2.8–6.2 cm^−1^ for the twelve vibrational modes considered.

Some studies enable a comparison of almost all fundamentals, excluding always the two OH stretching modes that are difficult to assign in the experiment because of the various resonances. The computation of anharmonic effects through VPT2 by Pitsevich *et al.*^181^ reduced the deviations to around 19.1 cm^−1^ (inlcuding 22 fundamentals). The VSCF results by Qu and Bowman show deviations of 33.5 cm^−1^,^119^ and for VCI to a MAD of 22.1 cm^−1^ ^120^ (including 21 fundamentals), while AIMD simulations reached about 12.3 cm^−1^ (including 20 fundamentals). Shanavas Rasheeda *et al.* have demonstrated VPT2 calculations on a high-dimensional neural network potential (HDNNP), which was based on a refined version of the Bowman2016 PES. With a MAD of 4.5 cm^−1^ (including 22 fundamentals) these calculations represent some of the most accurate theoretical descriptions of the dimerâ€™s vibrational structure to date.^122^

In the present work, a new PES was combined with a consistent hierarchy of vibrational models, ranging from harmonic and VPT2 to VSCF and VCI, to reassess the full vibrational spectrum of the formic acid dimer in a systematic comparison to the monomer. Our VCI and VPT2 computations yield MADs of 3.9 cm^−1^ and 7.5 cm^−1^ (including 22 fundamentals), respectively, in line with or slightly improving upon previous high-level results. Taken together, these developments highlight the continuous refinement of both PES construction and vibrational methodology.

The results approach the definition of spectroscopic accuracy we mentioned before for MI-FTIR spectroscopy. As shown in Table 7, we can confidently say that for the low-frequency part of the spectrum (<1500 cm^−1^), spectroscopic accuracy is reached, and the VCI and VPT2 calculations provide reliable foundations for interpreting MI-FTIR spectra of the formic acid cyclic dimer. However, in the high-frequency part of the spectrum, gas phase assignments are less certain, and the analysis of deviations becomes less reliable. In contrast, in the MI-FTIR spectra, it is straightforward to assign all fundamentals with detectable intensities.

## 4 Conclusion

In this work, we establish a fully consistent experimental and theoretical description of the vibrational spectroscopy of the formic acid monomer and its cyclic dimer across multiple isotopocules over the entire mid-IR region (4000–500 cm^−1^). To this end, we performed a series of MI-FTIR measurements on several isotopocules of formic acid isolated in neon and argon matrices at 5.8 K and different dilution ratios. In parallel, we computed *ab initio* N-mode expansions of the potential energy surfaces for the *trans*-formic acid monomer in *C*_s_ symmetry and its nonpolar cyclic dimer in *C*_2h_ symmetry. Based on these PESs, VCI and VPT2 calculations were carried out for HCOOH, HCOOD, DCOOH, DCOOD, (HCOOH)_2_, and (DCOOD)_2_. Together, these results provide the first internally consistent mid-IR reference dataset that combines matrix-isolation spectroscopy with high-level anharmonic calculations for both the monomeric and dimeric forms of formic acid.

The direct combination of experiment and anharmonic calculations enables a one-to-one assignment of essentially all experimentally observed spectral features, including overlapping bands, resonances, overtone and combination bands, as well as monomer and dimer signatures that cannot be unambiguously separated by experiment alone. At the same time, the excellent agreement with gas phase literature data, primarily obtained from supersonic jet expansions, demonstrates the accuracy of the newly developed PESs and establishes a new benchmark for computational spectroscopy of formic acid. For the fundamentals of the HCOOH isotopocule, the mean absolute deviations amount to approximately 2 cm^−1^ for VCI and 3 cm^−1^ for VPT2 in the monomer, and about 4 cm^−1^ for VCI and 8 cm^−1^ for VPT2 in the dimer. Even smaller deviations are obtained for the deuterated isotopocules.

Beyond the assignment of individual bands, the present study demonstrates that anharmonic calculations can become indispensable for disentangling anharmonicity from matrix-induced perturbations in MI-FTIR spectra. In particular, they allow reliable separation of monomer and dimer contributions even when both species coexist in a single experimental spectrum, as is typically the case for formic acid. Building on these assignments and a careful comparison with earlier studies, we further identify the dominant matrix effect as a matrix-induced frequency shift, with average values of approximately 3–7 cm^−1^ in argon and 1–3 cm^−1^ in neon for the monomer isotopocules. Although these average shifts are relatively small, their strongly mode-dependent character underscores the need for future computational approaches that can explicitly describe local matrix environments, for example, through embedding techniques.

More generally, the methodology presented here provides a transferable framework for combining MI-FTIR spectroscopy with anharmonic calculations in hydrogen-bonded molecular systems. At the same time, the present work defines the upper limit of spectral detail and assignment reliability currently achievable for routine MI-FTIR investigations. While fully resolved treatments of larger conformational landscapes and molecular aggregates rapidly become computationally demanding, the formic acid dimer provides an ideal benchmark system for developing simplified assignment strategies based on characteristic dimerization-induced frequency shifts. In part II of this series, we will build upon the reference data established here to address the challenges associated with extending such routine spectroscopic analyses to more complex conformational and aggregation phenomena of formic acid.^174^

## Author contributions

Dennis F. Dinu: conceptualization, data curation, formal analysis, investigation, methodology, validation, visualization, writing – original draft, writing – review & editing. Lukas Meinschad: data curation, formal analysis, investigation, methodology, validation, visualization, writing – original draft, writing – review & editing. Jonas Schlagin: formal analysis, investigation, visualization, writing – original draft, writing – review & editing. Vincent Enders: investigation, validation, writing – review & editing. Maren Podewitz: supervision, writing – review and editing. Dominik Stolzenburg: funding acquisition, project administration, supervision, writing – review & editing. Guntram Rauhut: formal analysis, investigation, validation, software, methodology, supervision, writing – original draft, writing – review & editing. Thomas Loerting: conceptualization, funding acquisition, methodology, project administration, resources, supervision, writing – review & editing. Hinrich Grothe: funding acquisition, methodology, project administration, resources, supervision, writing – review & editing. Klaus R. Liedl: conceptualization, funding acquisition, methodology, project administration, resources, supervision, writing – review & editing.

## Conflicts of interest

There are no conflicts to declare.

## Supplementary Material

CP-028-D6CP01818A-s001

## Data Availability

The data supporting this article have been included as part of the supplementary information (SI). Supplementary information is available. See DOI: https://doi.org/10.1039/d6cp01818a.
