# Supplementary material for: Vibrational spectra of formic acid and its dimer: I. Spectroscopic accuracy through matrix-isolation IR spectroscopy and anharmonic computations
Source: Phys Chem Chem Phys. 2026 Jul 22. Online ahead of print. doi: 10.1039/d6cp01818a (PMC13426427; doi:10.1039/d6cp01818a)
Supplement: CP-OLF-D6CP01818A-s001 [file CP-OLF-D6CP01818A-s001.pdf]

Supplementary Information (SI) for Physical Chemistry Chemical Physics.
This journal is © the Owner Societies 2026

Vibrational Spectra of Formic Acid and Its Dimer: I. Spectroscopic Accuracy through Matrix-Isolation IR Spectroscopy and Anharmonic Computations - Supplementary Information

Dennis F. Dinu, Lukas Meinschad, Jonas Schlagin, Vincent Enders, Maren Podewitz, Dominik Stolzenburg, Guntram Rauhut, Thomas Loerting, Hinrich Grothe, Klaus R. Liedl

July 10, 2026

Contents

1 Assignment of the Matrix Isolation FTIR Spectra from this Work 3
1.1 The νOH region [3600–3000 cm^{-1}] 4
1.1.1 νOH of **HH** & **DH** [3600–3550 cm^{-1}] 4
1.1.2 νOH of **(HH)$_2$** [3550–3500 cm^{-1}] 5
1.1.3 $2\nu_3$ of all isotopocules [3530–3450 cm^{-1}] 6
1.1.4 νOH of **(HH)$_2$** [3450–3300 cm^{-1}] 7
1.1.5 νOH of **(HH)$_2$** [3300–3000 cm^{-1}] 8
1.2 The νCH region [3000–2780 cm^{-1}] 9
1.2.1 νCH of **HH** & **HD** [3000–2900 cm^{-1}] 9
1.2.2 ν_3+ν_6 of **HH**, ν_3+ν_5 / ν_5+2ν_8 resonance of **DH**, and ν_3+ν_4 / ν_4+2ν_8 resonance of **DD**) [2940–2780 cm^{-1}] 10
1.3 The νOD region [2710–2250 cm^{-1}] 11
1.3.1 ν_5+ν_6+ν_7 of **HD**, and ν_3+ν_6 / ν_5+2ν_8 resonance of **DD** [2710–2650 cm^{-1}] 11
1.3.2 νOD of **HD** & **DD** [2700–2600 cm^{-1}] 12
1.3.3 ν_6+2ν_9/ν_4+ν_5 resonance of **HH** [2410–2300 cm^{-1}] 13
1.3.4 νOD of **(DD)$_2$** [2320–2250 cm^{-1}] 15
1.4 The νCD region [2250–1800 cm^{-1}] 16
1.4.1 $2\nu_6$ of **HH**, νCD of **DH**, νCD / $\nu_4+\nu_5(A')$ resonance of **DD**, νCD of **(DD)$_2$** [2250–2190 cm^{-1}] 16
1.4.2 $\nu_5+2\nu_9$ / $\nu_5+\nu_6$ resonance of **HD**, $\nu_5+\nu_6$ of **DH**, $\nu_4+\nu_6$ of **DD**, and $\nu_4+\nu_5$ / ν_2 resonance of **DD** [2300-2100 cm^{-1}] 17
1.4.3 $2\nu_5$ of **DD** [2100–2000 cm^{-1}] 18
1.4.4 $2\nu_6$ and $\nu_4+\nu_7$ of **DH** [1950–1780 cm^{-1}] 19
1.5 The νC=O region [1800–1600cm^{-1}] 20
1.5.1 νC=O of all isotopocules [1780–1700 cm^{-1}] 20
1.5.2 νC=O of the dimers [1760–1640 cm^{-1}] 21

1.6 The δ_{ip}CH, δ_{ip}COH, and νC−Oregion [1410–1080cm^{-1}] 22
1.6.1 δ_{ip}CH of **HH**, **HD** & δ_{ip}COH of **(HH)$_2$** [1410–1340 cm^{-1}] 22
1.6.2 δ_{ip}CH of **(HH)$_2$** [1420–1390 cm^{-1}] 23
1.6.3 δ_{ip}COH of **HH** (& resonance), νC−O of **(HH)$_2$**, **(DD)$_2$** [1310–1190 cm^{-1}] 24
1.6.4 νC−O region [1190–1080 cm^{-1}] 25
1.7 The δ_{oop}CH region [1080–840 cm^{-1}] 26
1.7.1 δ_{oop}CH of **(HH)$_2$** & δ_{ip}COD of **(DD)$_2$** [1080–1050 cm^{-1}] 26
1.7.2 δ_{oop}CH of **HH**, **HD** & δ_{ip}COD of **DD** [1080–1010 cm^{-1}] 27
1.7.3 δ_{ip}COD of **HD** & δ_{ip}CD of **DH**, **DD**, and **(DD)$_2$** [1010–940 cm^{-1}] 28
1.7.4 δ_{oop}COH of **(HH)$_2$**, δ_{ip}CD of **(DD)$_2$** [1000–920 cm^{-1}] 29
1.7.5 δ_{oop}CD of **DH**, **DD** [920–840 cm^{-1}] 30
1.7.6 δ_{oop}CD of **(DD)$_2$** [920–880 cm^{-1}] 31
1.8 Dimer region [840–650 cm^{-1}] 32
1.8.1 The "empty" spectrum [840–740 cm^{-1}] 32
1.8.2 δ_{ip}OCO of **(HH)$_2$** & δ_{oop}COD of **(DD)$_2$** [740–680 cm^{-1}] 33
1.8.3 δ_{ip}OCO of **(DD)$_2$** & **CO$_2$** ν_2 [740–650 cm^{-1}] 34
1.9 The δ_{oop}COH & δ_{ip}OCO region [650–500 cm^{-1}] 35
1.9.1 δ_{oop}COH & δ_{ip}OCO of **HH** & **DH** [650–600 cm^{-1}] 35
1.9.2 δ_{oop}COH & δ_{ip}OCO of **HD** & **DD** [580–530 cm^{-1}] 36
1.10 Water and carbon dioxide contamination 37
1.10.1 **D$_2$O** and **HDO** [2840–2700 cm^{-1}] 37
1.10.2 **CO$_2$** [2360–2220 cm^{-1}] 38
1.10.3 **HDO** [1600–1400 cm^{-1}] 39

2 Compilation of Vibrational Assignments and Reference Spectroscopic Data 40

3 Computational Accuracy for Different Computational Methods 47

4 Normal Mode Decomposition of the Formic Acid Monomer 50
4.1 Contribution heatmap and vibrational notation for trans-HH 53
4.2 Contribution heatmap and vibrational notation for trans-HD 54
4.3 Contribution heatmap and vibrational notation for trans-DH 55
4.4 Contribution heatmap and vibrational notation for trans-DD 56

5 Normal Mode Decomposition of the Formic Acid Cyclic Dimer 57
5.1 Contribution heatmap and vibrational notation for (*trans*−HH)$_2$ 59
5.2 Contribution heatmap and vibrational notation for (DD)$_2$ 61

6 Resonance Analysis of the Monomer 63

7 Supplementary References 65

1 Assignment of the Matrix Isolation FTIR Spectra from this Work

This Supplementary Information presents the matrix isolation Fourier transform infrared (MI-FTIR) spectra as obtained and analyzed in the present work. In the assignment presented here, our main focus lies on the *trans*-formic acid monomer and its cyclic dimer with C_{2h} symmetry. We observe several additional bands that correspond to other conformers (cf. Lopes *et al.*[1]). The assignment of these bands is subject to another study of this series.

All MI-FTIR spectra presented here follow a consistent format to facilitate comparison across isotopocules and experimental conditions. Each spectrum shows formic acid isolated in argon (red) and neon (blue) MI-FTIR spectra, with matrix-to-analyte ratios typically of 1:1000, 1:500, and 1:250, plotted with decreasing line color intensity. The isotopic composition of the sample is indicated in the background using the shorthand notation **HH** = HCOOH, **HD** = HCOOD, **DH** = DCOOH, and **DD** = DCOOD. Reference gas phase (GP) spectra from the literature[2] are shown in yellow for comparison, while anharmonic vibrational configuration interaction (VCI) spectra computed in this work are displayed in purple at the top of each spectrum.

Isotopic exchange during sample preparation makes it difficult to completely avoid minor impurities of **HH** in the **HD** and **DD** samples, and vice versa. This effect is particularly pronounced when the matrix is deposited at low gas flow rates into the cryostat, as was initially the case in our experiments. To mitigate this problem, additional matrices for the **HD** and **DD** samples were prepared using rapid deposition without a mass flow controller. For the partially and fully deuterated species, this fast-deposition approach reduces isotopic exchange at the **OH** site.

In some figures, spectra recorded under slow-deposition conditions, comparable to those used for the **HH** and **DH** samples, are included as dashed lines to illustrate the impact of isotopic exchange during slow deposition. Despite these precautions, a certain degree of isotopic impurity could not be entirely eliminated. As shown, for example, in the **HD** and **DD** panels of Figure 1.1.1 (dashed lines = slow deposition; solid lines = fast deposition), the extent of isotopic exchange decreases significantly under fast-deposition conditions, although a small fraction of exchanged species remains.

1.1 The νOH region [3600–3000 cm^{-1}]

1.1.1 νOH of HH & DH [3600–3550 cm^{-1}]

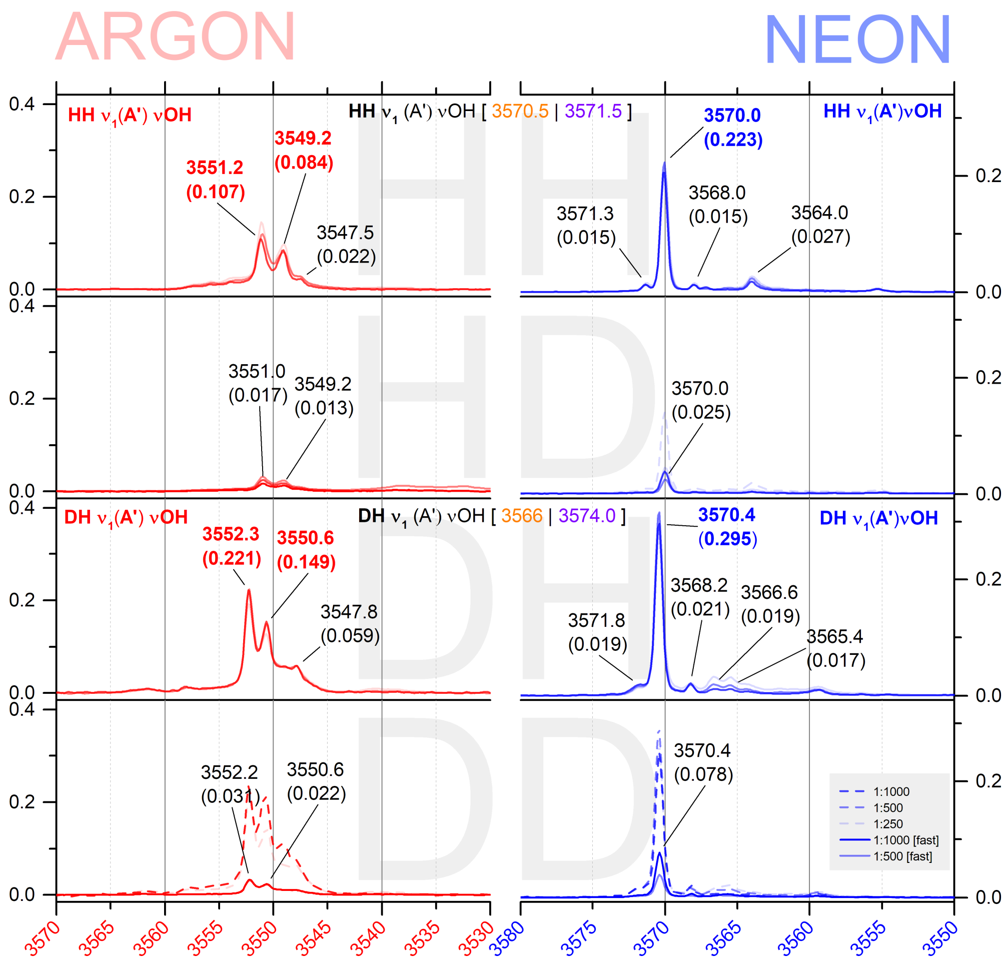

- In this spectral region, we observe the νOH vibration. While this is expected for the **HH** and **DH** samples, the νOH vibration is also visible in the **HD** and **DD** samples as a result of isotopic exchange. The corresponding νOD vibration of **HD** and **DD** is shown in Figure 1.3.2.
- The νOH vibration of **HH** in gas phase (3570.5 cm^{-1}) Infrared spectra differs slightly to Raman jet experiments, where a resonance pair between 3570 and 3567 cm^{-1} was assigned.[2] In contrast, no such resonance has been reported in the literature for **DH**. In **DH**, the gas phase (3566 cm^{-1}) value is slightly lower compared to its counterpart in **HH**, while the Raman jet experiment it lies in between (3569 cm^{-1}).
- According to VCI calculations, however, the situation appears more complex. The calculated νOH stretching frequency of **HH** in VCI (3571.5 cm^{-1}) is only slightly lower than the corresponding frequency of **DH** in VCI (3574.0 cm^{-1}). In both cases, VCI predicts at least one resonance partner occurring near 3563 cm^{-1}, significantly below the values reported for the Raman jet experiments.
- For **HH** in neon (3570.0 cm^{-1}) we observe a possible resonance partner at 3564.0 cm^{-1}. For **DH** in neon (3570.4 cm^{-1}) we observe a similar feature with a possible resonance partner at 3565.4 cm^{-1}. However, the lower frequency component is of very weak intensity and may also originate from a dimer species. **HH** exhibits a distinct band splitting in argon (3551.2 and 3549.2 cm^{-1}), while **DH** shows a comparable splitting in argon (3552.3 and 3550.6 cm^{-1}). This is more likely a matrix effect than a resonance, and the observations are in good agreement with previous results reported by Macoas *et al.*[3].

1.1.2 νOH of $(HH)_2$ [3550–3500 cm^{-1}]

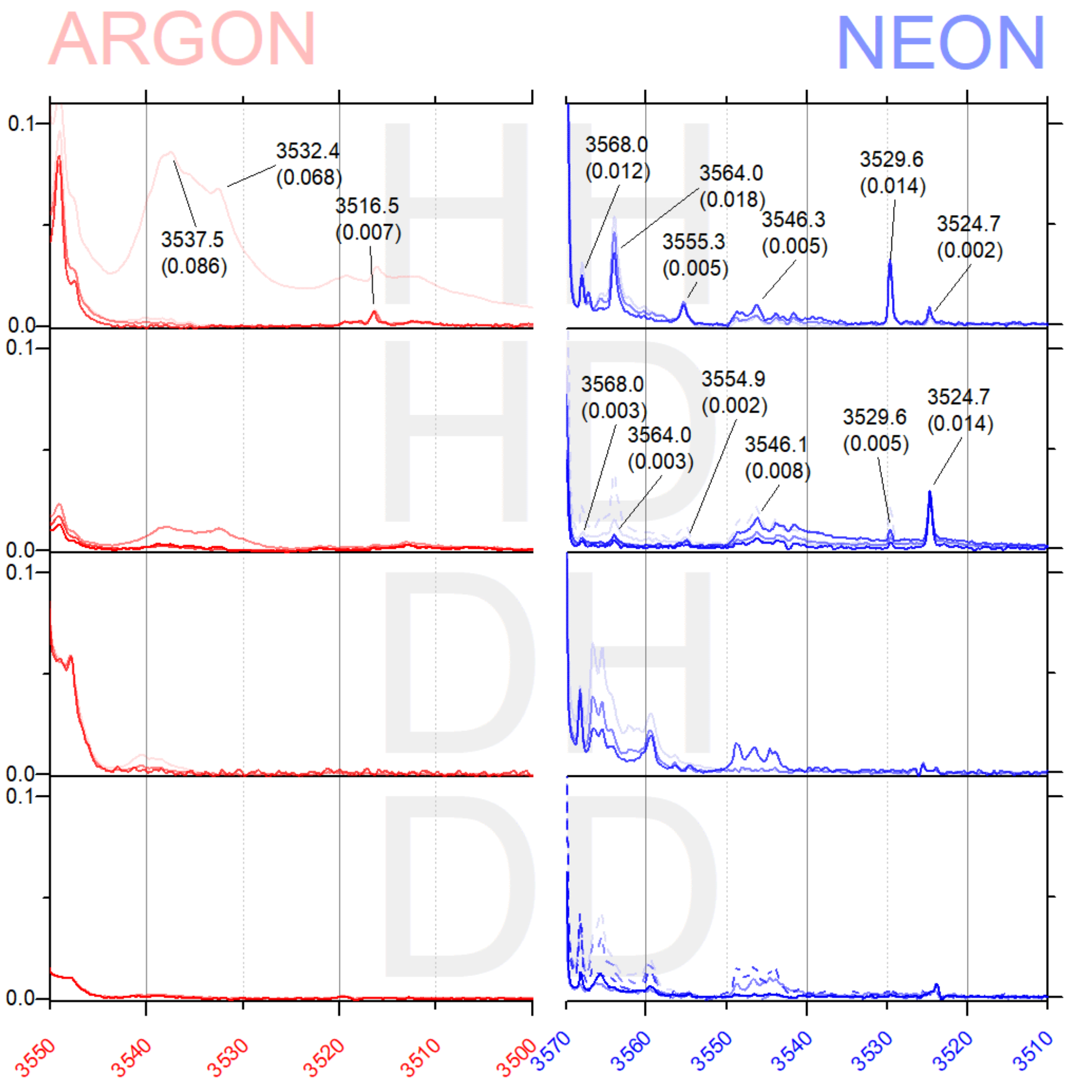

- This spectral regions includes bands that are likely associated with the νOH vibration of dimers, containing *"free"* OH groups that do not participate in hydrogen bonding. For example, we can argue that there must be a dimer conformer where only one hydrogen-bond is formed. Here, the bands that are slightly redshifted relative to the monomer in argon (3537.5 cm^{-1}) and in neon (3564.0 cm^{-1}) MI-FTIR spectra can be attributed to the vibration of the "free" OH group. In addition, the bands in the 3500–3300 cm^{-1} region (as shown in Figure 1.1.4) may be due to the vibration of the "bond" OH group.

1.1.3 $2\nu_3$ of all isotopocules [3530–3450 cm^{-1}]

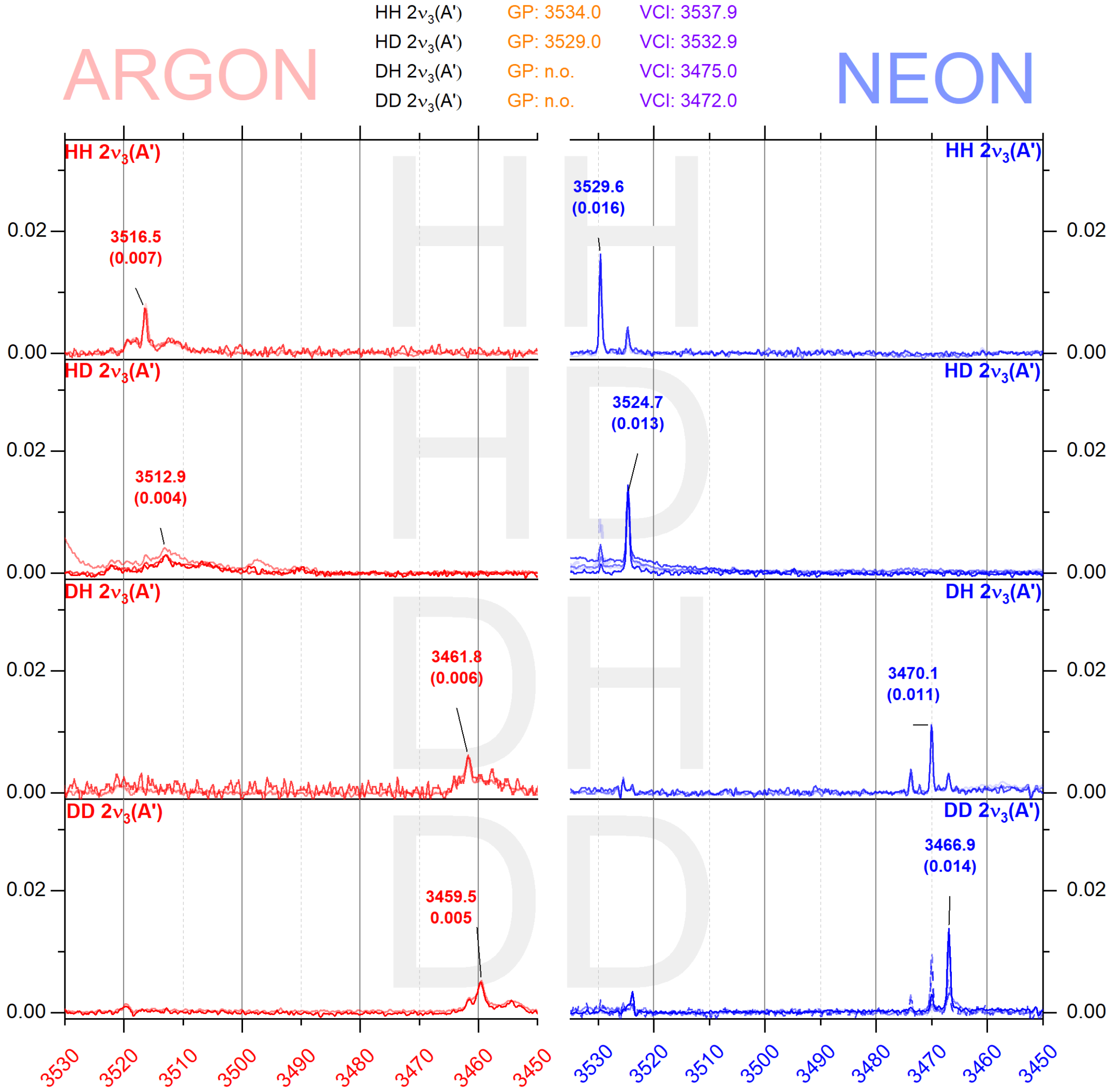

- In this spectral region, we observe the overtone $2\nu_3$ for all isotopocules. Compared to the gas phase data of Nejad[2], matrix shifts of -4.4 cm^{-1} for **HH** and -4.3 cm^{-1} for **HD** are observed in neon MI-FTIR spectra.
- Isotopic exchange of H/D is observed at the OH group, but not at the aliphatic position. The isotopic shifts are in overall agreement between the gas phase spectrum, the neon MI-FTIR spectrum, and the VCI calculations, with deviations of about 0.1 cm^{-1}.
- The assignment of $2\nu_3$ of **HD** in the argon MI-FTIR spectrum is consistent with Marushkevich *et al.*[4], showing a deviation of 0.8 cm^{-1}.
- For **HH**, the band position of $2\nu_3$ shows a larger deviation of 2.5 cm^{-1} compared to the argon MI-FTIR spectrum reported by Macoas *et al.*[3], likely due to broader spectral features.
- The situation differs for **DH** and **DD**, as no gas phase reference data are available. The assignment cannot be supported by consistent matrix shifts. Nevertheless, for **DH** the assignment of $2\nu_3$ is consistent with argon MI-FTIR spectra reported by Macoas *et al.*[3], with a deviation of 0.5 cm^{-1}. No matrix isolation spectroscopy literature data is available for $2\nu_3$ of **DD**.
- In **DD**, however, isotopic exchange at the OH position is again observed. The resulting isotopic shift of 3.2 cm^{-1} is in excellent agreement with the value of 3.0 cm^{-1} predicted by VCI calculations, allowing the assignment of $2\nu_3$ of **DD**.

1.1.4 νOH of (HH)$_2$ [3450–3300 cm^{-1}]

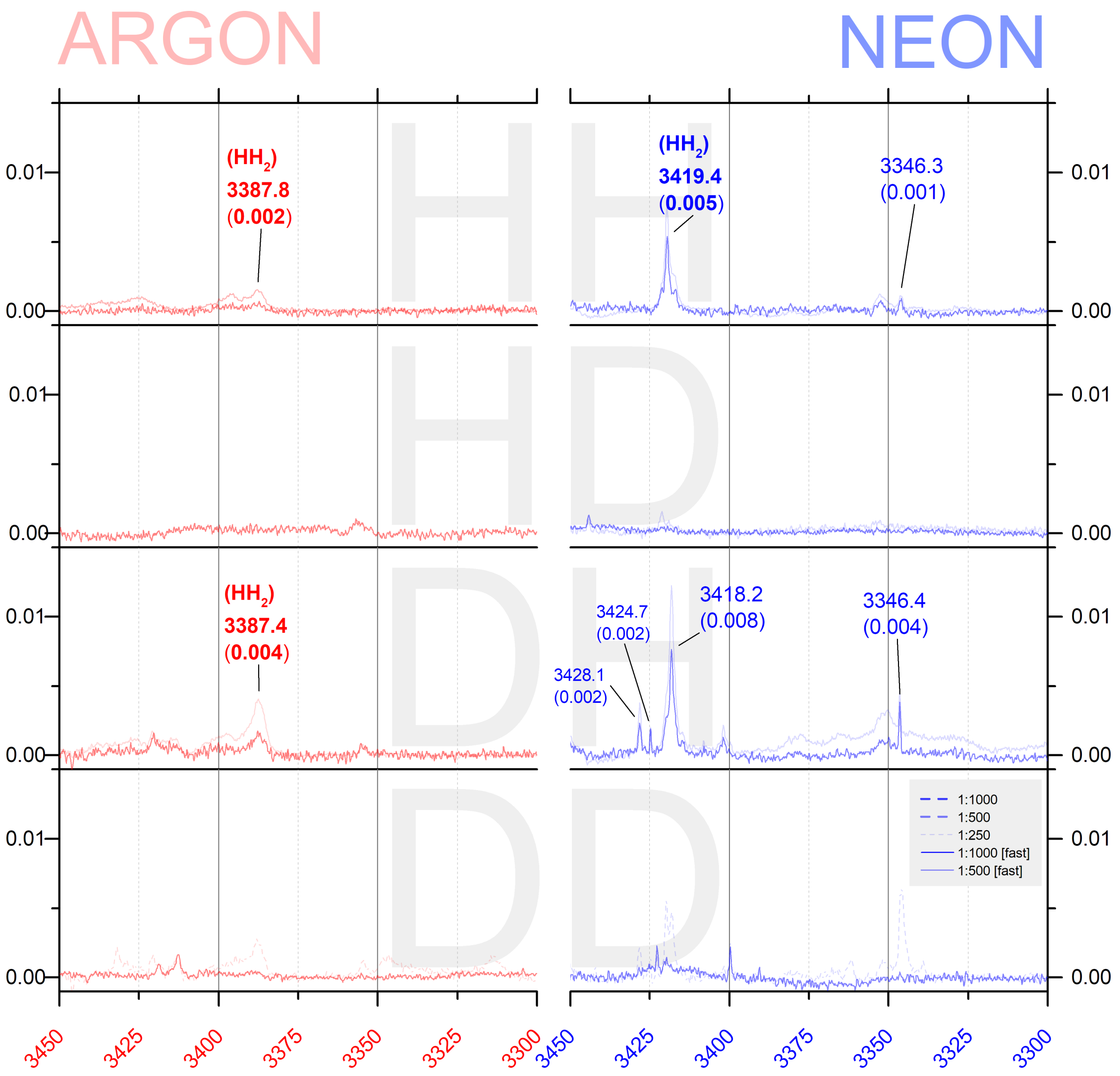

- In this spectral region, the spectrum of **HD** is basically empty, while several bands appear in the **DH** spectrum, some of which are also observed in the **HH** and **DD** spectra.
- For the **HH** sample we observe in the neon (3419.6 cm^{-1}) MI-FTIR spectrum a band that is also present in the **DH**. We may assign this to the νOH of **HH** dimer, in analogy to a corresponding band in argon (3387.8 cm^{-1}) observed in the **HH** and **DH** samples, which was previously assigned by Lopes *et al.* to the "free" νOH vibration of and higher energy conformer with a *"chain"* of OH groups.[1]
- Another band in neon (3346.3 cm^{-1}) MI-FTIR spectra is also present in the **HH** and **DH** samples. Its assignment remains uncertain based on the spectra discussed here.

1.1.5 νOH of $(HH)_2$ [3300–3000 cm^{-1}]

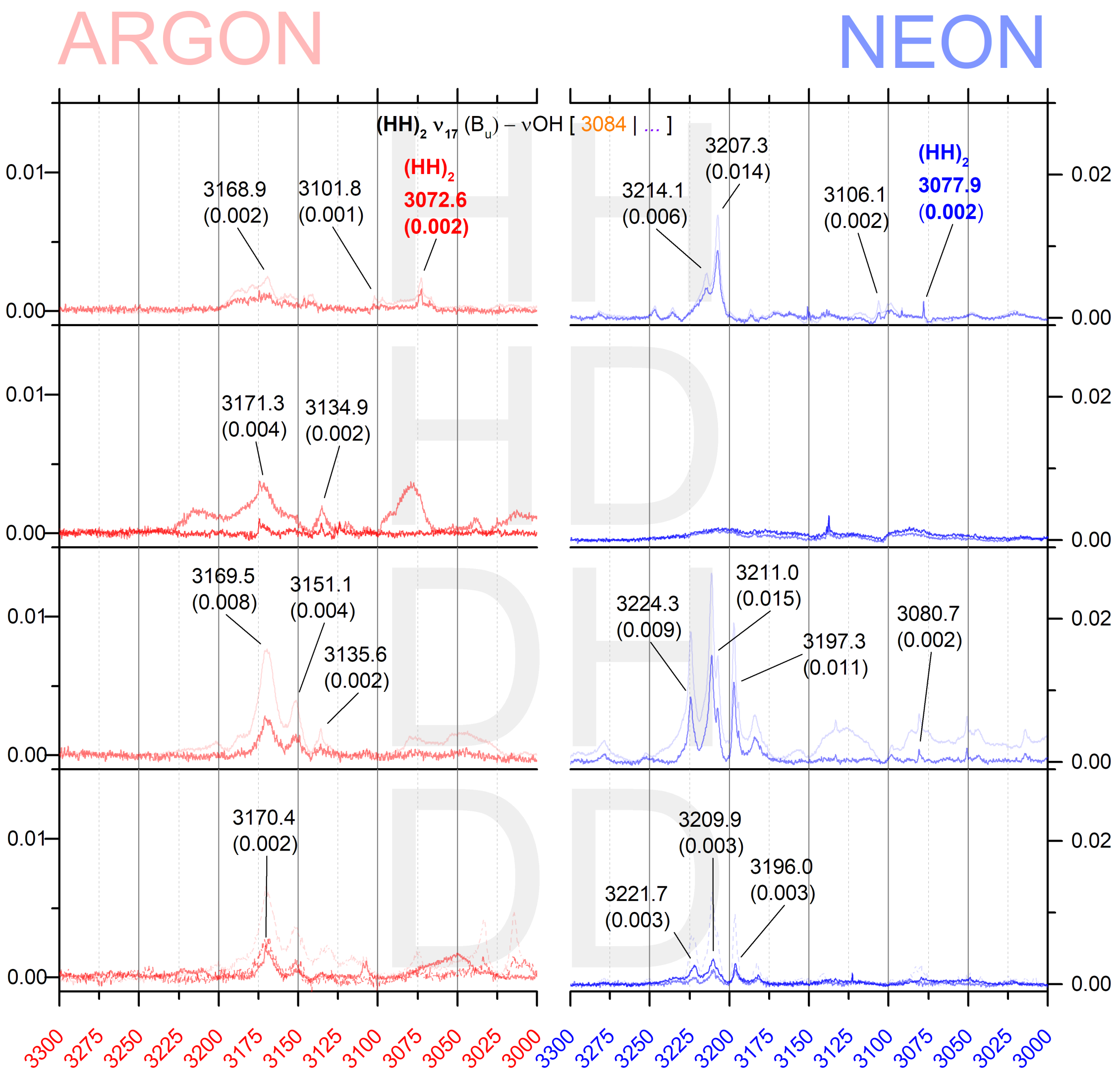

- We observe the νOH vibration of the lowest-energy dimer conformer (cyclic double-hydrogen bonded) with low intensity in argon (3072.6 cm^{-1}) and in neon (3078 cm^{-1}) MI-FTIR spectra.
- The studies by Lopes *et al.* further suggest that the pattern of bands between 3250 and 3140 cm^{-1} can be assigned to higher energy conformers of the dimer.[1] In these conformers, the OH group of one subunit remains *"free"*, while the other forms a hydrogen bond with the carbonyl group, similar to the cyclic dimer. In the present MI-FTIR spectra, several weak features in this range support this assignment.

1.2 The νCH region [3000–2780 cm^{-1}]

1.2.1 νCH of HH & HD [3000–2900 cm^{-1}]

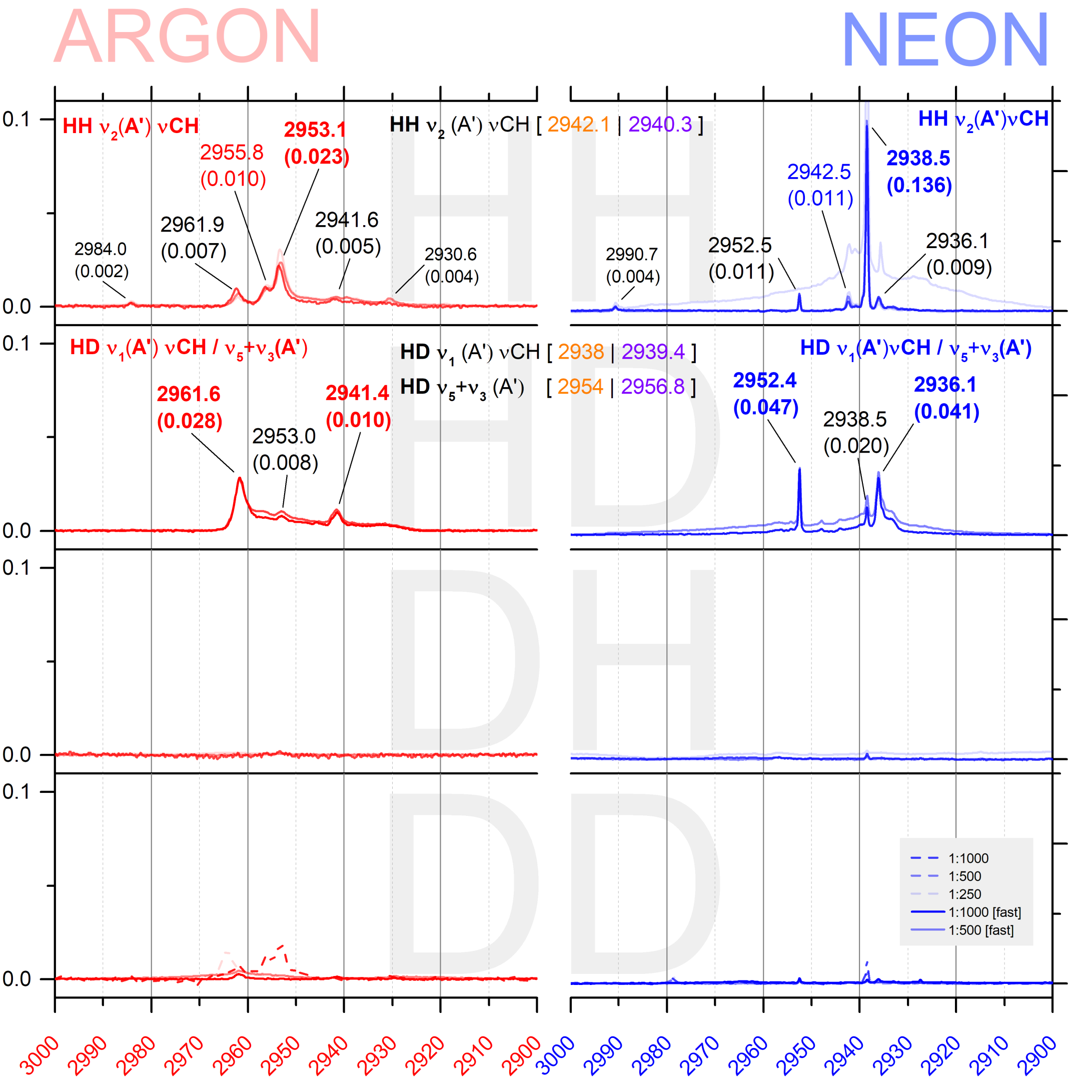

- In this spectral region the νCH vibration is expected with relatively low intensity. Although it is not easily detectable, several features can be unambiguously assigned. Note that bands from the **HH** spectrum appear as contamination in the **HD** spectrum, and vice versa.

- In the **HH** spectrum, the νCH vibration is observed both in argon (2953.1 cm^{-1}) and in neon (2938.5 cm^{-1}) MI-FTIR spectra. These values are in good agreement with the gas phase (2942.1 cm^{-1}) reference and the VCI (2940.3 cm^{-1}) calculation. In the **HD** spectrum, the νCH vibration, or $\nu_2(A')$, appears as a resonance with $\nu_5 + \nu_3(A')$, in argon (2961.6, 2941.4 cm^{-1}) and in neon (2952.4, 2936.1 cm^{-1}) MI-FTIR spectra. These values are in close agreement with the gas phase (2954, 2938 cm^{-1}) reference and the VCI (2956.8, 2939.4 cm^{-1}) calculations. For the **DH** and **DD** samples, the corresponding νCD vibration is observed at lower wavenumbers (cf. Figure 1.4.1).

- In this region, the νCH stretching vibration of the **(HH)$_2$** dimer is expected but not observed in our MI-FTIR spectra, likely due to low intensity. Gas-phase studies report strong overlap between monomer and dimer bands, yet still assign monomer frequencies (e.g., 2942.1 cm^{-1}, Freytes et al.[5]). Georges et al. also note this overlap of monomer and dimer, but report inconsistent values for the dimer, including 2938.5 cm^{-1} in their tables, which is widely used as a reference, and 2957 cm^{-1} in their text. Our variational calculations (VCI (2955 cm^{-1})) nicely agree with the latter and may suggests that the commonly accepted gas-phase frequency for the νCH stretching vibration of the **(HH)$_2$** dimer may be wrong.

1.2.2 $\nu_3+\nu_6$ of HH, $\nu_3+\nu_5$ / $\nu_5+2\nu_8$ resonance of DH, and $\nu_3+\nu_4$ / $\nu_4+2\nu_8$ resonance of DD) [2940–2780 cm^{-1}]

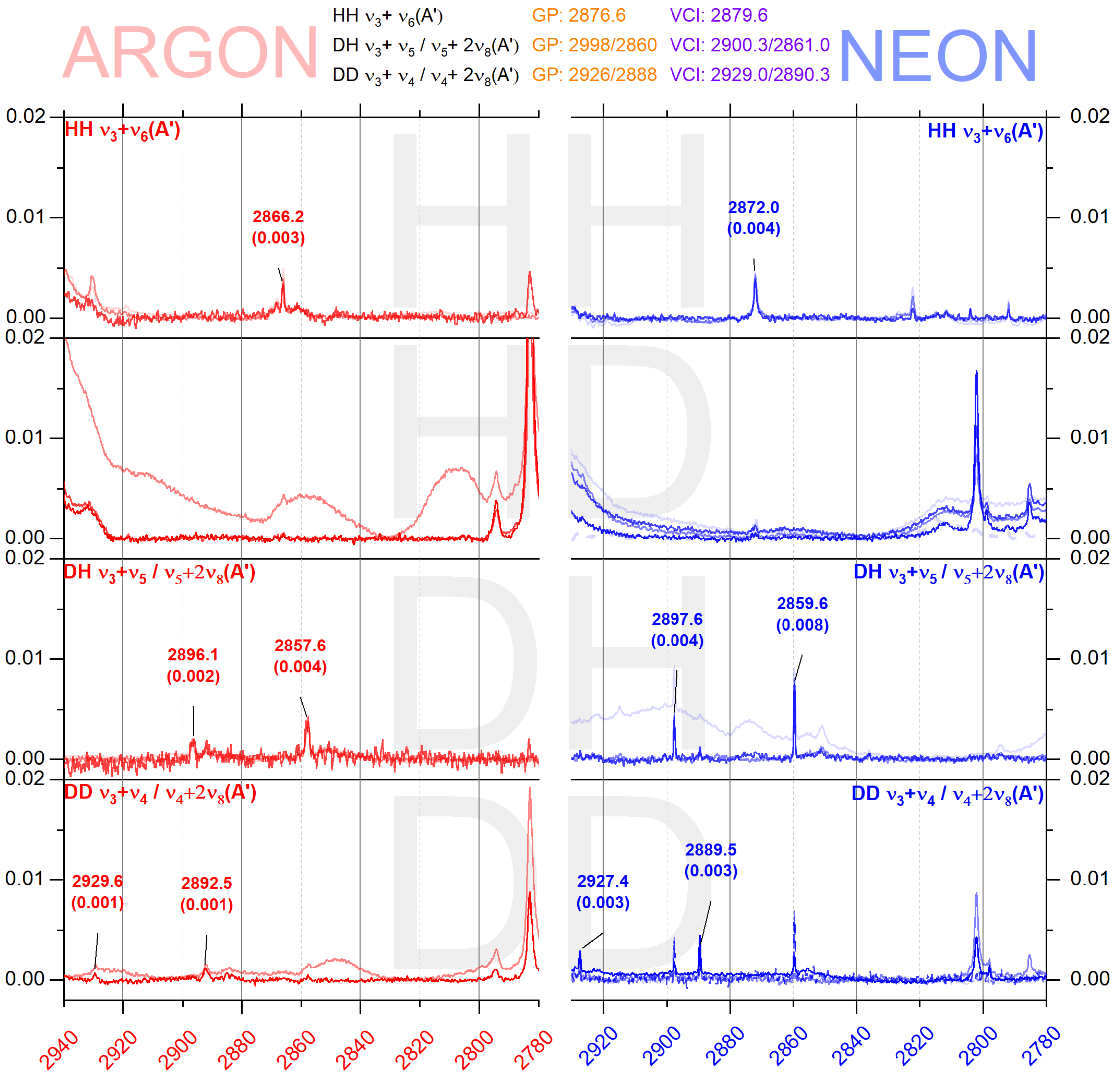

- We observe and assign multiple sharp bands in this spectral region: **HH** in neon (2872.0 cm^{-1}) and in argon (2866.9 cm^{-1}), **DH** in neon (2897.6, 2859.6 cm^{-1}) and in argon (2896.1, 2857.6 cm^{-1}), **DD** in neon (2927.4, 2889.5 cm^{-1}) and in argon (2929.6, 2892.5 cm^{-1}).
- These bands exhibit constant relative intensities across different dilution rates, supporting their assignment to vibrational transitions of the monomer rather than to dimer species or matrix trapping site effects. As these bands correspond to different vibrational transitions, consistent shifts such as those observed in Figure 1.1.3 are not expected.
- For the **HH** combination band, the absence of other features with comparable intensity facilitates the assignment. To the best of our knowledge, no reference data in argon or neon MI-FTIR spectra are available in the literature. Therefore, only the band positions and the corresponding matrix shifts of -4.6 and $-10.4\ \mathrm{cm}^{-1}$ can be reported.
- For the resonance states of the **DH** and **DD** isotopocules, highly consistent shifts are observed across the individual resonances, as expected for such coupled states. In the neon MI-FTIR spectra, the shifts amount to $-0.4/-0.4\ \mathrm{cm}^{-1}$ for the bands related to **DH** and $1.4/1.5\ \mathrm{cm}^{-1}$ for bands related to DD. The VCI calculations accurately reproduce the band-to-band separations, with deviations of 1.3 cm^{-1} for the resonances related to **DH** and 1.0 cm^{-1} for the resonances related to **DD**, strongly supporting the assignment of these states.

1.3 The νOD region [2710–2250 cm^{-1}]

1.3.1 $\nu_5+\nu_6+\nu_7$ of HD, and $\nu_3+\nu_6$ / $\nu_5+2\nu_8$ resonance of DD [2710–2650 cm^{-1}]

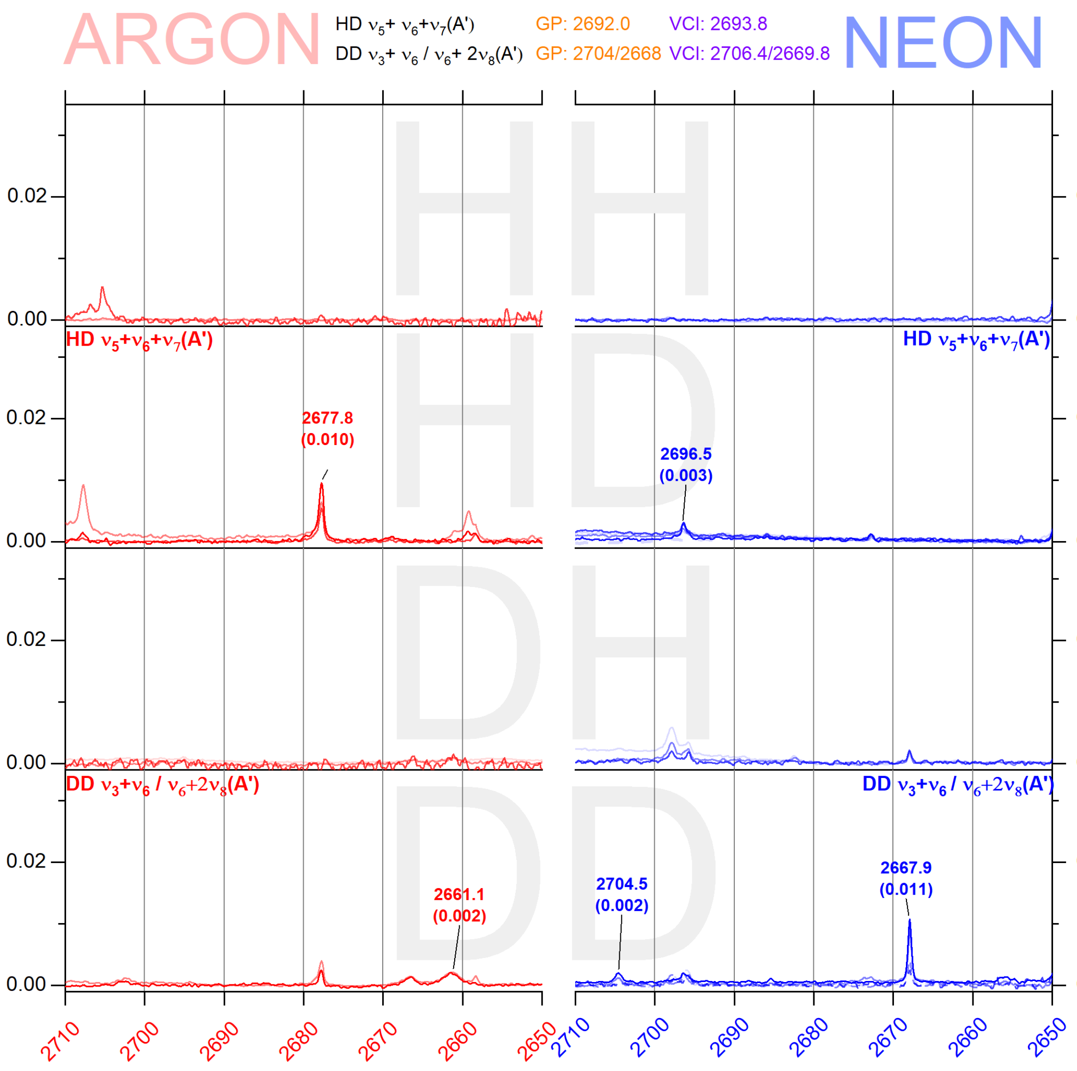

- In the neon MI-FTIR spectra, three bands are observed whose normalized intensities remain constant with changing dilution rates. For the **HD** sample, we observe each on band in the neon (2696.5 cm^{-1}) and in the argon (2677.8 cm^{-1}) MI-FTIR spectrum. For the **DD** sample, we observe two bands in neon (2704.5 and 2667.9 cm^{-1}), while only one band in argon (2661.1 cm^{-1}). Another a weak feature in argon (ca. 2702 cm^{-1}) could be considered, but will be neglected due to the lack of clear baseline separation.
- For the **HD** combination band, no reference data in argon or neon MI-FTIR spectra are available. However, the constant peak shape, the matrix shift of 4.5 cm^{-1} in the neon MI-FTIR spectrum, and the agreement with VCI calculations (with a deviation of 1.8 cm^{-1} between gas phase data and VCI values) strongly support the assignment of this band.
- For the **DD** resonance, no MI-FTIR reference data are available for comparison. Nevertheless, consistent matrix shifts of 0.5 and -0.1 cm^{-1} relative to the gas phase are observed. Also, the VCI calculations accurately reproduce the band-to-band separation, with a deviation of 0.6 cm^{-1}, further supporting the assignment.

1.3.2 νOD of HD & DD [2700–2600 cm^{-1}]

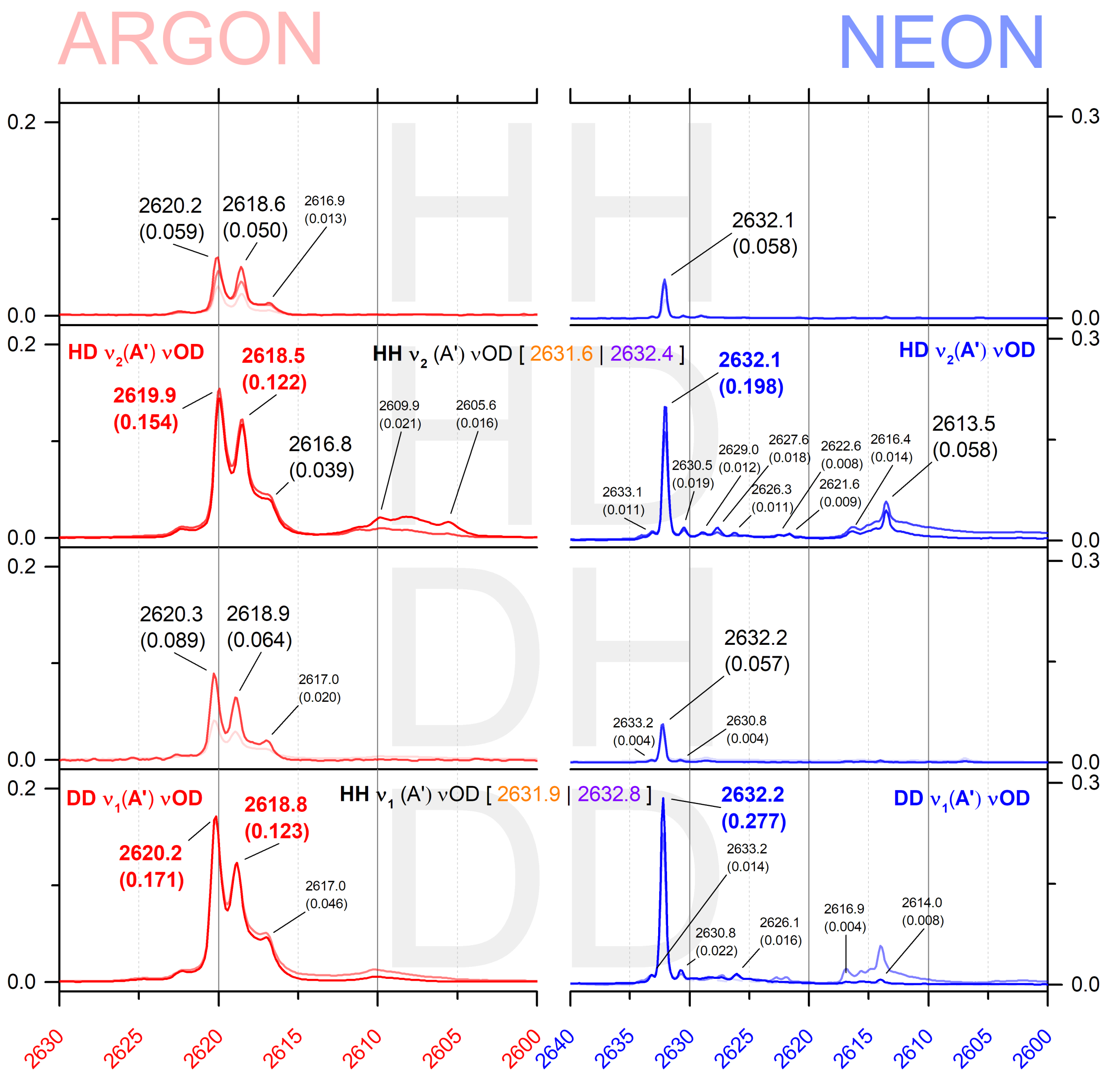

- In this spectral region, we observe the νOD vibration of **HD** and **DD**. These bands are also present in the **HH** and **DH** spectra, most likely due to isotopic exchange. The corresponding νOH vibrations of **HH** and **DH** occur at much higher wavenumbers (cf. Figure 1.1.1).
- For **HD**, the bands appear in argon (2619.9, 2618.5 cm^{-1}) MI-FTIR spectra with characteristic matrix-site splitting, and in neon (2632.1 cm^{-1}) with no such splitting. The observation is in good agreement with gas phase (2631.6 cm^{-1}) reference and VCI (2632.4 cm^{-1}) calculation.
- Similarly for **DD**, the corresponding bands occur in argon (2620.2, 2618.8 cm^{-1}) in neon (2632.2 cm^{-1}) MI-FTIR spectra. The observation is in good agreement with gas phase (2631.9 cm^{-1}) reference and VCI (2632.8 cm^{-1}) calculation.
- Additional, comparatively weak absorptions are observed in argon (2610 cm^{-1}) and in neon (2614 cm^{-1}) MI-FTIR spectra. In the neon MI-FTIR experiments, the intensities of these bands exhibit a pronounced dependence on matrix dilution, indicating that they are more plausibly associated with dimeric species than with monomeric vibrational transitions. Nonetheless, these bands cannot be assigned to the cyclic dimer of C_{2h} symmetry, since the corresponding νOD vibrational mode for this structure is predicted to occur at substantially lower wavenumbers.

1.3.3 $\nu_6+2\nu_9/\nu_4+\nu_5$ resonance of HH [2410–2300 cm^{-1}]

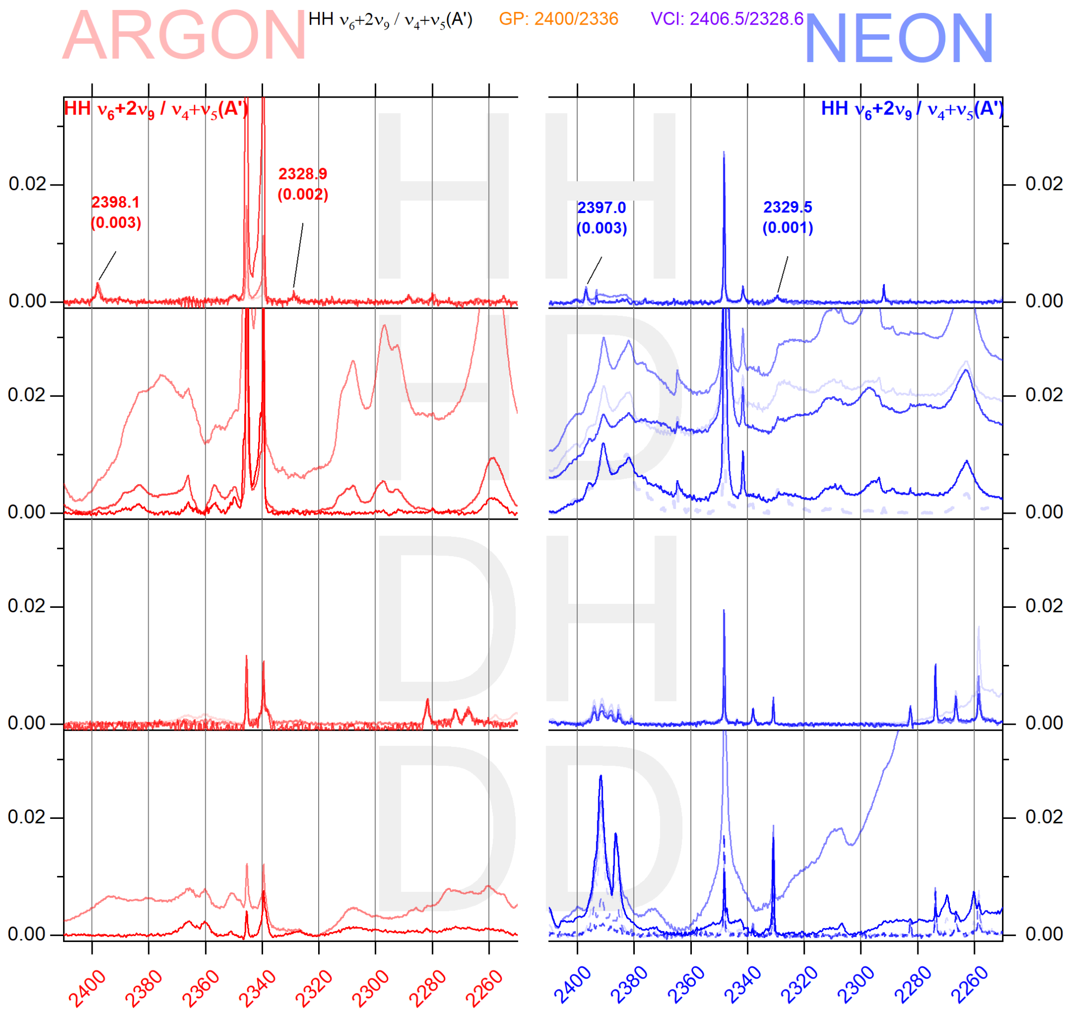

- The intense bands here are due to the antisymmetric stretching vibration of $\mathbf{CO_2}$. Many additional bands that decrease in intensity upon increasing dilution originate from $\mathbf{CO_2}$ aggregates.

- In the **HH** spectra, two additional bands are observed whose normalized intensities remain constant over the investigated dilution range. These bands appear in neon (2397.0, 2329.5 cm^{-1}) and in argon (2398.1, 2328.9 cm^{-1}) MI-FTIR spectra. The band-to-band separation obtained from the VCI (2406.5, 2328.6 cm^{-1}) calculations agrees well with the value expected from gas phase (2400, 2336 cm^{-1}) reference data, with a deviation of only 3.5 cm^{-1}. Upon changing the host matrix from neon to argon, both bands exhibit comparable shifts of 1.1 cm^{-1} and -0.6 cm^{-1}, respectively, which supports their assignment as coupled (ambiguous) states.

- One band in argon (2398.1 cm^{-1}) MI-FTIR spectrum was previously assigned by Macoas *et al.*[3], although not within the context of a resonance. Their anharmonic reference data were obtained from one-dimensional VSCF calculations based on a PES computed at the MP2/6-311++G(2d,2p) level of theory. This methodology leads to discrepancies between calculated and experimental transition energies and fails to adequately describe vibrational resonances. Nejad[6] assigned this band to a different resonance, namely $\nu_6 + 2\nu_9$ / $4\nu_9$, and interpreted the feature at 2328.9 cm^{-1} as a non-resonant (unique) state. Although this alternative assignment may be plausible, it cannot be rigorously evaluated within the present framework, as the computational protocol is limited to excitations up to triple quanta. This restriction was imposed deliberately because the computational effort increases steeply with higher excitation levels, whereas the associated intensities decrease rapidly, rendering such transitions unlikely to be detected experimentally.

1.3.4 νOD of $(DD)_2$ [2320–2250 cm^{-1}]

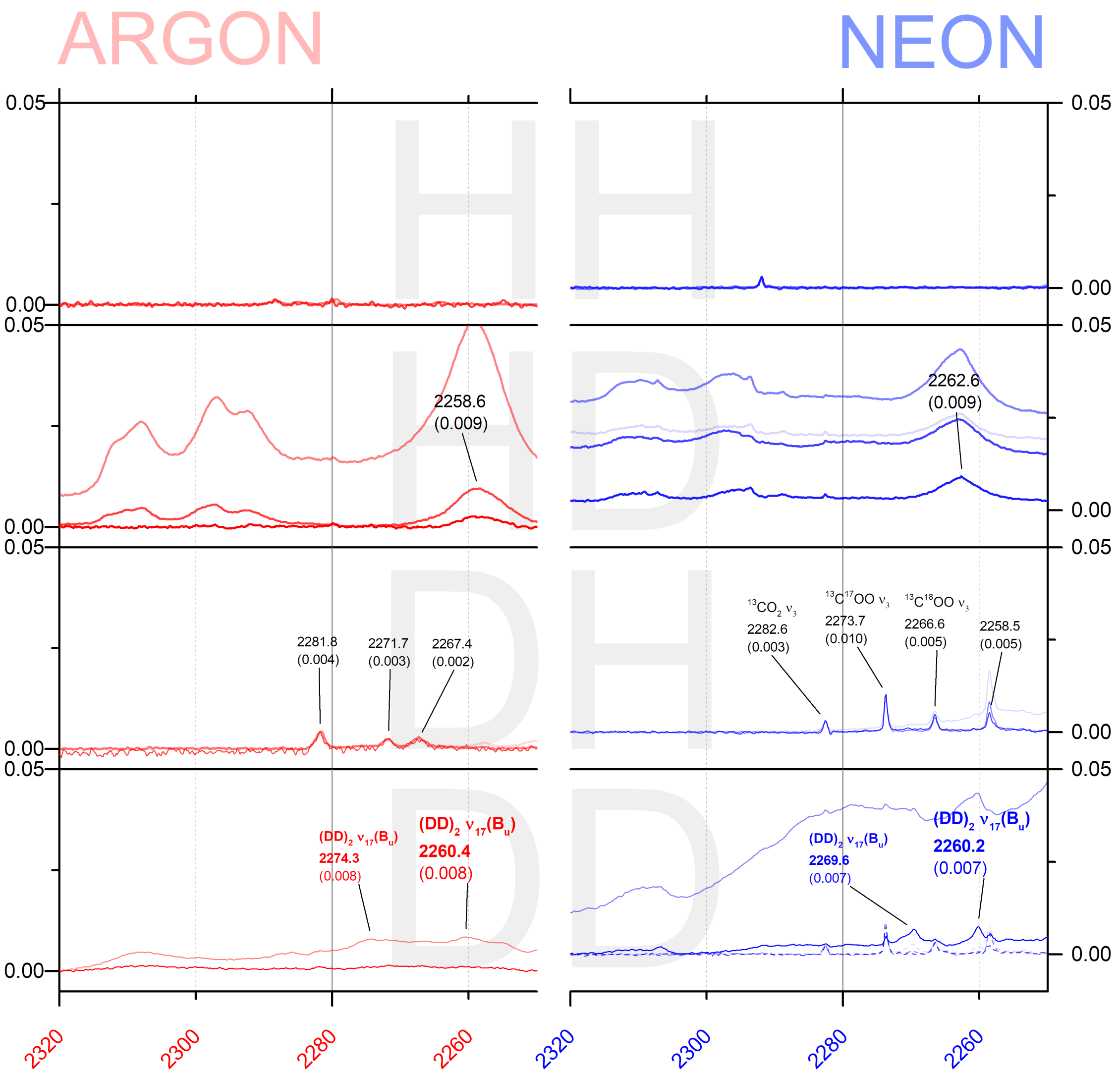

- In this region, we expect the νOD stretching vibration of the cyclic dimer for the $(DD)_2$ isotopocule based on gas phase (2270 cm^{-1}) reference. The VCI (2280, 2269, 2249, 2244, 2165 cm^{-1}) calculation of the cyclic $(DD)_2$ dimer yields a complex pattern of resonances. It is likely that we obtain numerical artifacts from the truncation of the VCI space.
- Ultimately, we consider the value of 2269 cm^{-1} to be the VCI result for the νOD stretching vibration, as it agrees well with experiment and our VPT2 (2273 cm^{-1}) calculation. A more detailed investigation of these resonances is beyond the scope of the present work. Accordingly, in the **DD** sample, the broad bands in argon (2260.4 cm^{-1}) and neon (2260.2 cm^{-1}) MI-FTIR spectra are tentatively assigned to the νOD stretching vibration of the cyclic $(DD)_2$ dimer.
- In the **HD** spectrum, where the νOD stretching vibration of the cyclic $(HD)_2$ dimer may be expected, similar broad bands are observed at slightly shifted frequencies. However, we do not proceed with this assignment here.
- Several sharp bands appear in both argon (2281.8, 2271.7, 2267.4 cm^{-1}) and neon (2282.5, 2273.7, 2266.6 cm^{-1}) MI-FTIR spectra of the **DH** sample under slow-deposition conditions. These arise from the ν_3 mode of various CO_2 isotopocules (see also Figure 1.10.2). Similar contamination is seen in the neon MI-FTIR spectra of the **DD** sample recorded under slow deposition, but not in spectra of the same sample obtained under fast deposition on a different day. In these slow-deposition experiments, CO_2 contamination was particularly strong.

1.4 The νCD region [2250–1800 cm^{-1}]

1.4.1 $2\nu_6$ of HH, νCD of DH, νCD / $\nu_4 + \nu_5(A')$ resonance of DD, νCD of $(\mathrm{DD})_2$ [2250–2190 cm^{-1}]

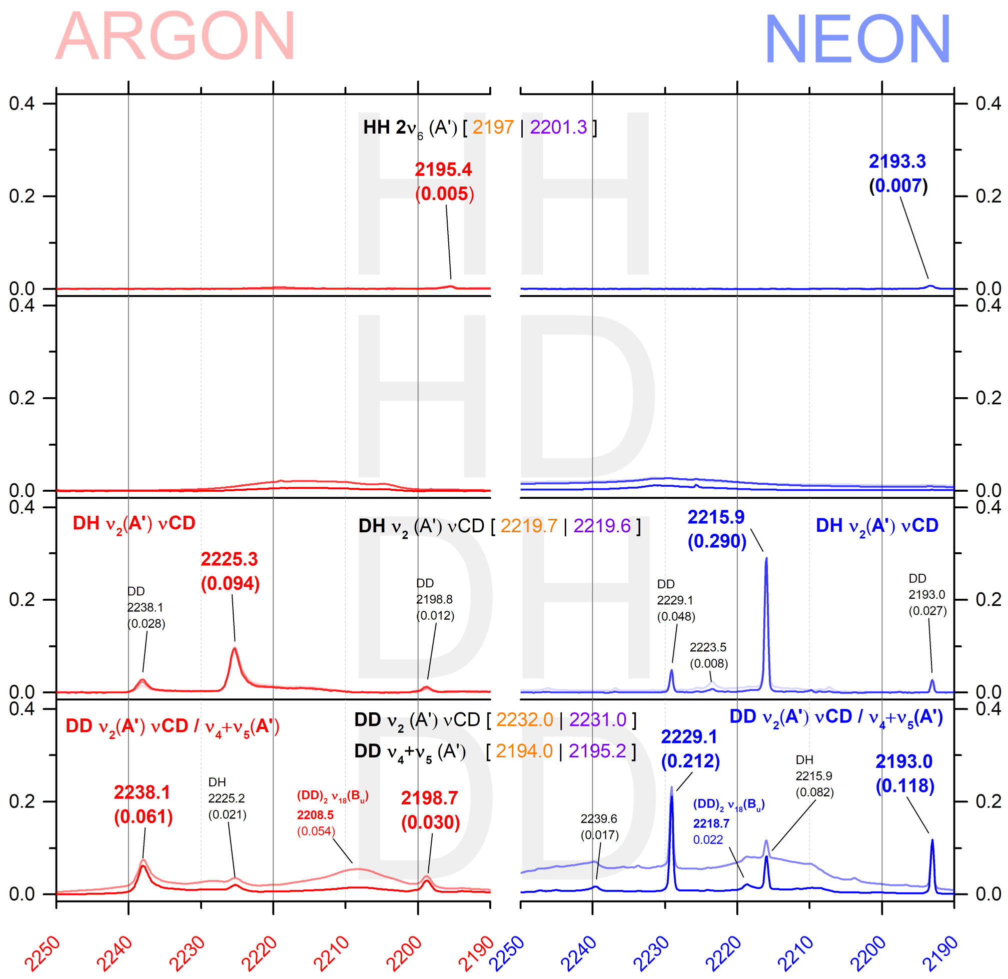

- In the spectra of the **HH** sample, a weak band is observed in both argon (2195.4 cm^{-1}) and neon (2193.3 cm^{-1}) MI-FTIR spectra. It maintains a constant normalized intensity across different dilution ratios. The VCI (2201.3 cm^{-1}) calculation predicts the $2\nu_6$ overtone in this region, with a deviation of 5 cm^{-1} from the gas phase (2197 cm^{-1}) value. This assignment is further supported by previous studies by Macoas *et al.*[3] and Nejad[2], who likewise attribute this band to the $2\nu_6$ mode of **HH**.
- The νCD vibration, or $\nu_2(A')$, of the **DD** isotopocule s is in resonance with the combination band $\nu_4 + \nu_5(A')$. We observe it both in the argon (2238.1 and 2198.7 cm^{-1}) and in neon (2229.1 and 2193.0 cm^{-1}) MI-FTIR spectra. This agrees well with VCI (2231.0 and 2195.2 cm^{-1}) calculation and gas phase (2232 and 2195.0 cm^{-1}) reference data.
- For **DH**, no such resonance is expected. We observe a single band corresponding to νCD ($\nu_2(A')$) in argon (2225.3 cm^{-1}) and neon (2215.9 cm^{-1}) MI-FTIR spectra. These values agree well with the gas phase (2219.7 cm^{-1}) reference and the VCI (2219.6 cm^{-1}) calculation.
- The νCD vibration of the $(\mathbf{DD})_2$ dimer is predicted by VCI (2214 cm^{-1}), but no gas phase reference is available. In the lower dilution experiment (1:500), this vibration can be assigned to bands in argon (2208.5 cm^{-1}) and in neon (2218.7 cm^{-1}) MI-FTIR spectra. However, this assignment remains tentative as the bands appear significantly broadened.

1.4.2 $\nu_5 + 2\nu_9$ / $\nu_5 + \nu_6$ resonance of HD, $\nu_5 + \nu_6$ of DH, $\nu_4 + \nu_6$ of DD, and $\nu_4 + \nu_5$ / ν_2 resonance of DD [2300-2100 cm^{-1}]

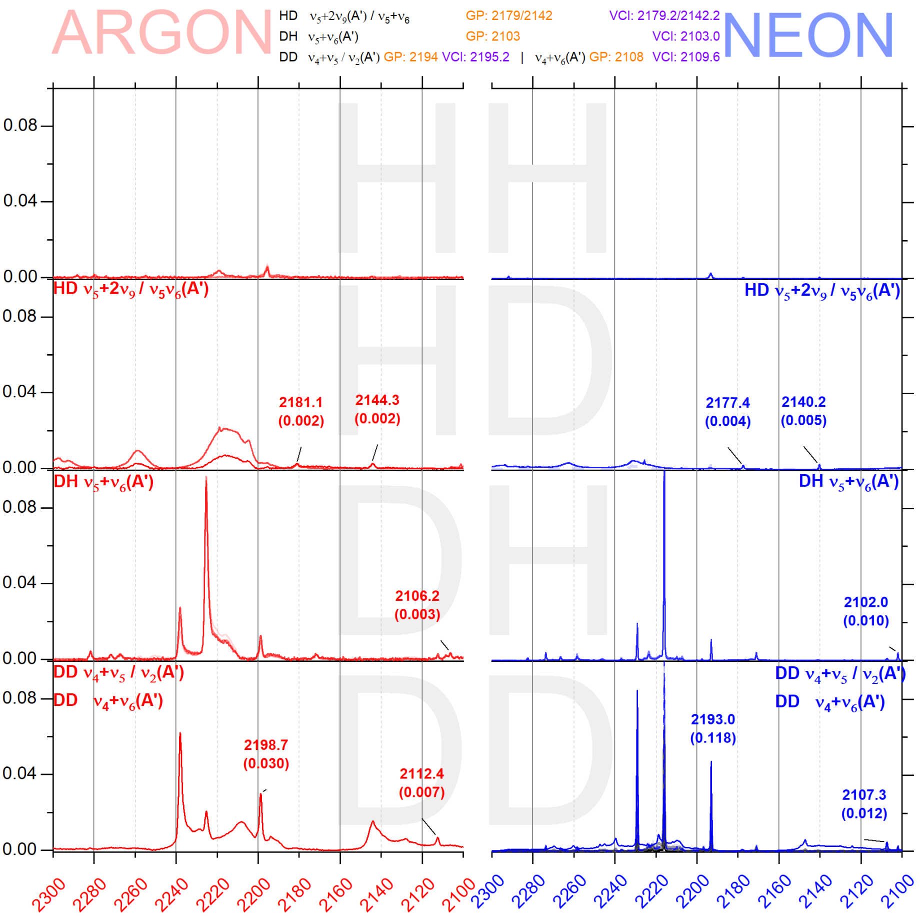

- We here show partially the same spectral region as in Figure 1.4.1. In addition to the dimer region of the **DD** isotopocule and the ν_2 bands of the **DH** and **DD** monomer, several further features are observed that exhibit constant normalized intensities across different dilution ratios.

- For the **HD** monomer, the VCI (2179.2, 2142.2 cm^{-1}) calculations predict a resonance that reproduces the gas phase (2179, 2142 cm^{-1}) band-to-band distance with a deviation of 0 cm^{-1}. In the neon (2177.4 and 2140.2 cm^{-1}) MI-FTIR spectrum, the shifts of both bands are -1.4 and -2.2 cm^{-1}, respectively, while the shifts upon changing the host matrix to argon (2181.1 and 2144.3 cm^{-1}) amount to 3.7 and 4.1 cm^{-1}. Similar features were reported by Williams *et al.*[7], although the resonance itself was not identified due to the lack of accurate reference calculations.

- For the **DH** and **DD** samples, isotopic exchange is again clearly observed in all spectra. The bands in argon (2106.2 cm^{-1}) and neon (2102.0 cm^{-1}) MI-FTIR spectra can be assigned to the **DH** isotopocule based on its pronounced decrease in intensity in the **DD** spectrum. This feature is therefore assigned to the $\nu_5 + \nu_6$ combination band, showing a neon matrix shift of -1.0 cm^{-1} and no deviation between the VCI calculation and the gas phase reference. Nejad[2], however, assigns this band to the $\nu_4 + \nu_6$ combination, which is not supported by the present calculations.

- For the **DD** samples, isotopic exchange allows a direct identification of the corresponding features. The VCI calculations reproduce the band-to-band distance with a deviation of 0.4 cm^{-1}. The assignment of $\nu_4 + \nu_6$ is consistent with the work of Nejad, whereas the assignment of $\nu_4 + \nu_5$ shows a similar discrepancy to the **DH** $\nu_5 + \nu_6$ band, which was also assigned differently by Nejad.

1.4.3 $2\nu_5$ of DD [2100–2000 cm^{-1}]

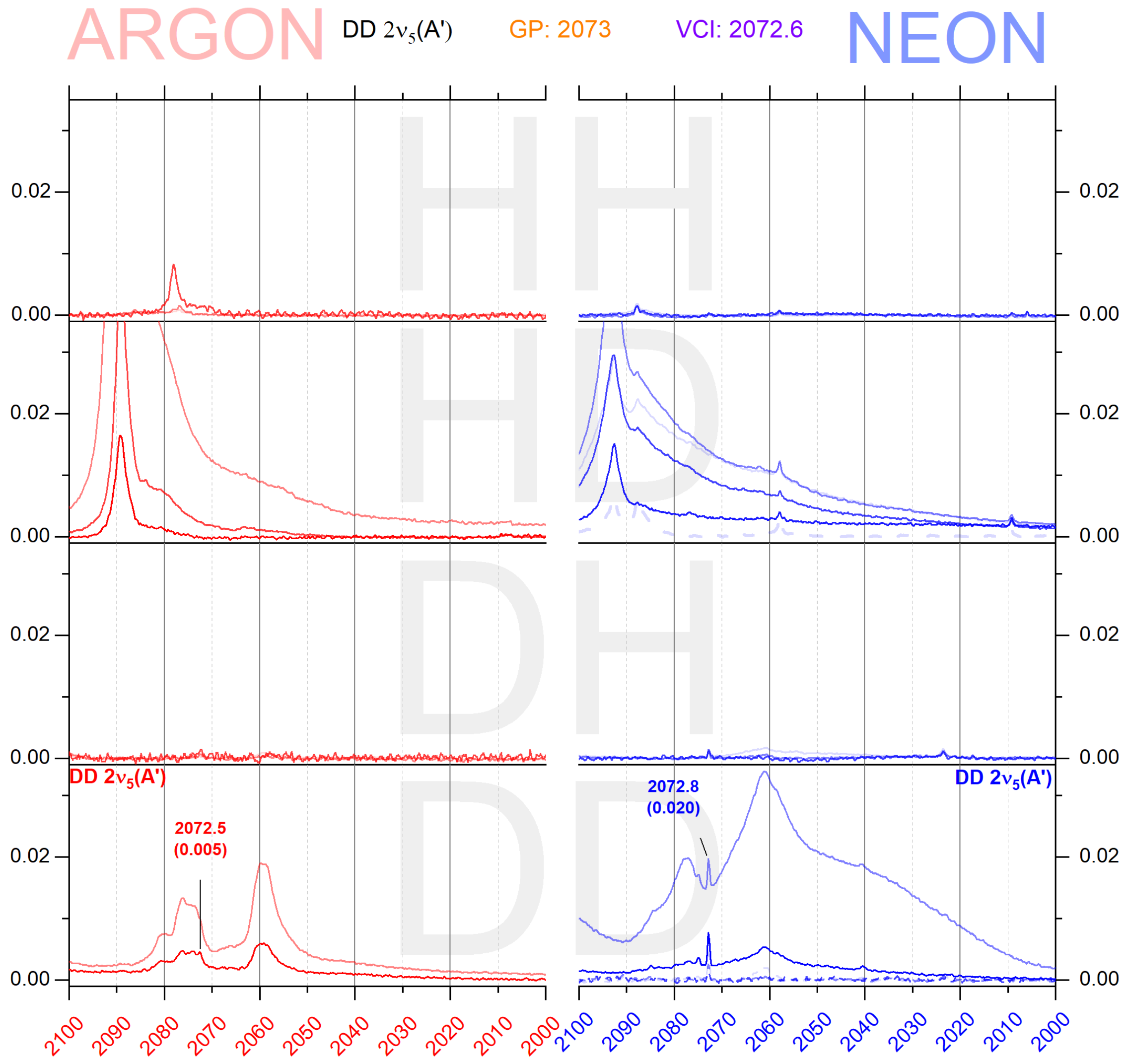

- In the spectral range between 2100 and 2000 cm^{-1} one band in the neon (2072.8 cm^{-1}) MI-FTIR spectrum of the **DD** sample maintains a constant normalized intensity across different dilution rates. Its assessment is difficult, as the band is fully embedded within broad spectral features (probably from aggregation) that raise the overall absorption baseline. The corresponding band in the argon (2072.5 cm^{-1}) MI-FTIR spectrum, is further obscured by poor spectral resolution. No literature data are available for these bands in argon or neon MI-FTIR spectra.
- The predicted $2\nu_5$ overtone from the VCI (2072.6 cm^{-1}) calculation is in very close proximity to this feature, with deviations of 0.4 cm^{-1} from the gas phase (2073 cm^{-1}) reference and 0.2 cm^{-1} from the neon (2072.8 cm^{-1}) MI-FTIR value. Note that in the gas phase reference (cf. Nejad[2]) the corresponding band is assigned as the $2\nu_4$ overtone. This interpretation is not supported by the present calculations and likely arises from differing assignments of the fundamental ν modes.

1.4.4 $2\nu_6$ and $\nu_4 + \nu_7$ of DH [1950–1780 cm^{-1}]

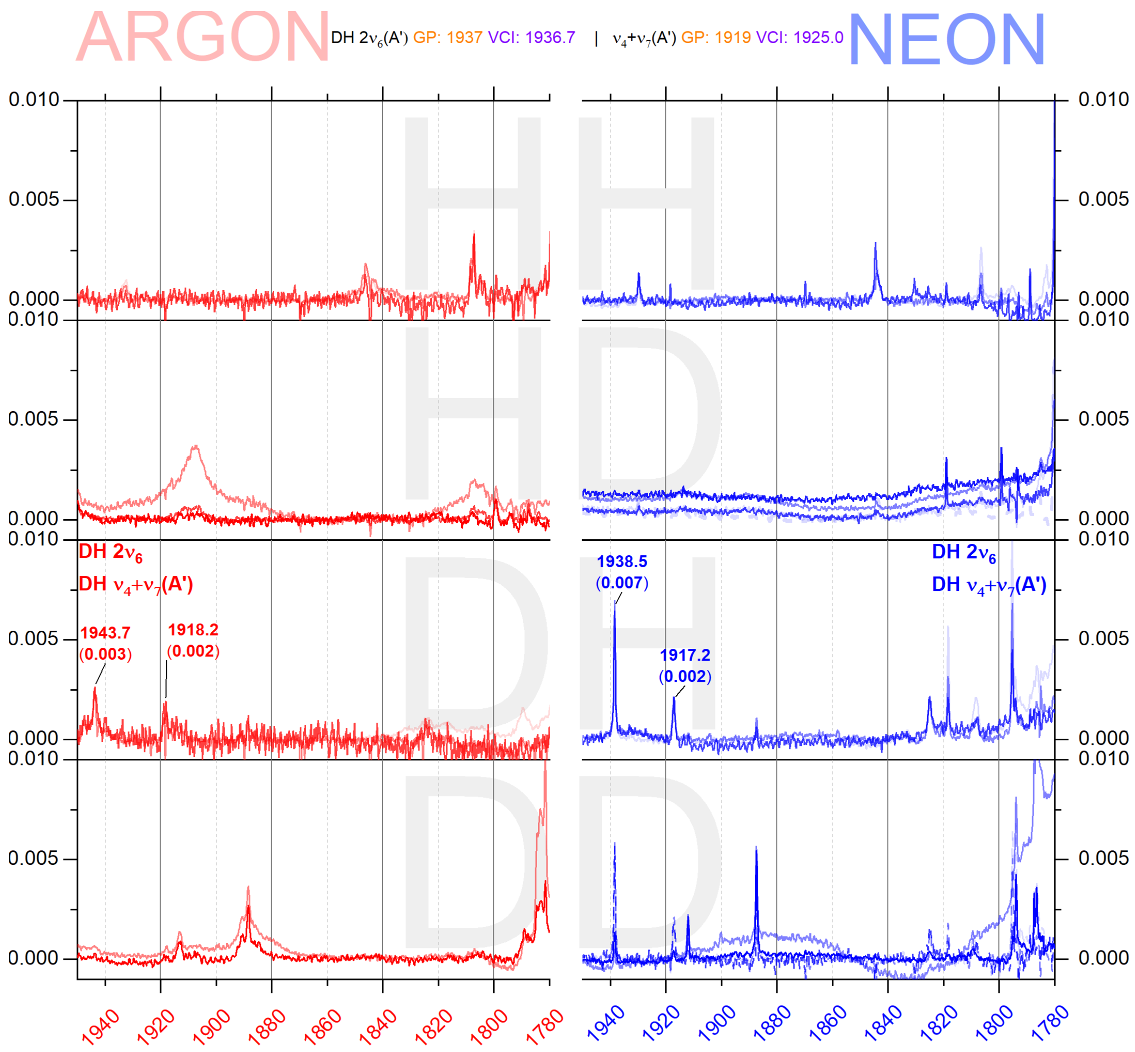

- In the shown region, we expect the $2\nu_6$ overtone and the $\nu_4 + \nu_7$ combination band of the **DH** isotopocule. Based on isotopic exchange relative to the **DD** spectrum, the bands corresponding to the **DH** isotopocule can again be identified unambiguously.
- For the $2\nu_6$ and $\nu_4 + \nu_7$, the VCI (1936.7, 1925.0 cm^{-1}) calculated band-to-band distance, shows a comparatively large deviation of 6.3 cm^{-1}, exceeding the discrepancies observed in our other assignments of combination bands and overtones. For the $\nu_4 + \nu_7$ combination band, we also observe a large discrepancy between VCI (1925.0 cm^{-1}) and gas phase (1919 cm^{-1}) reference values, which amounts to approximately 6 cm^{-1}. These increased deviations likely originate from an insufficient description of the VCI space. As discussed previously for the fundamental region, an expanded correlation space was required to accurately reproduce the ν_4 fundamental, and a similar extension is expected to improve the description of this combination band.
- However, we can rely our assignment on the observation that the assigned $2\nu_6$ and $\nu_4 + \nu_7$ vibrations, both in argon (1943.7 and 1918.2 cm^{-1}) and neon (1938.5 and 1917.2 cm^{-1}) MI-FTIR spectra, are the only ones that exhibit constant normalized intensities. As in previous cases, the assignment by Nejad[2] differs due to differences in the labeling of the fundamental modes. Also, to the best of our knowledge, no literature data are available for this band in argon or neon MI-FTIR spectra.

1.5 The νC=O region [1800–1600cm^{-1}]

1.5.1 νC=O of all isotopocules [1780–1700 cm^{-1}]

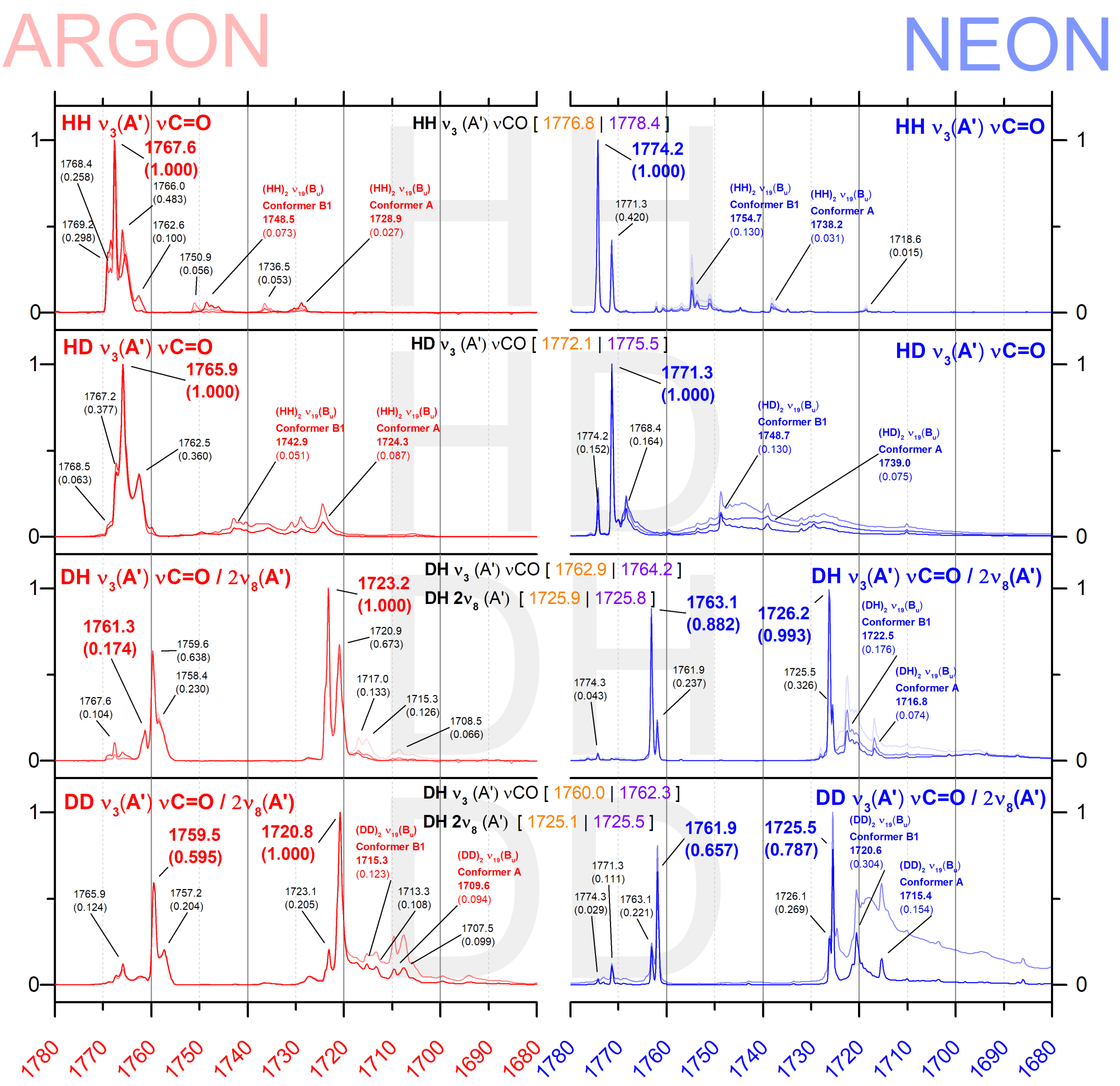

- The figure shows the prominent νC=O vibration of the monomer, which successively redshifts by several wavenumbers from the **HH** sample (argon (1767.6 cm^{-1}), neon (1774.2 cm^{-1})) to **HD** (argon (1765.9 cm^{-1}), neon (1771.3 cm^{-1})), **DH** (argon (1761.3 cm^{-1}), neon (1763.1 cm^{-1})), and **DD** (argon (1759.5 cm^{-1}), neon (1761.9 cm^{-1})).
- In argon MI-FTIR spectra, the band shape suggests a matrix-splitting effect; however, the presence of contaminants complicates a detailed analysis. This is particularly evident for **DH**, where the splitting cannot be resolved due to overlap with the νC=O band of the **DD** impurity in the same spectral region.
- In neon MI-FTIR spectra, mutual contamination between **HH** and **HD**, as well as between **DH** and **DD**, is also apparent. Nevertheless, the bands appear as well-defined single peaks without indications of matrix splitting and can be unambiguously assigned.
- For **DH** and **DD**, a Fermi resonance is expected between the νC=O vibration (ν_3) and the overtone of the in-plane δ_{ip}**CD** vibration ($2\nu_8$). This has been demonstrated in Raman jet experiments[2] and is also supported by the present VCI calculations.
- Additional bands that decrease in intensity with increasing dilution are observed and can be attributed to the νC=O vibration of the dimer.

1.5.2 νC=O of the dimers [1760–1640 cm^{-1}]

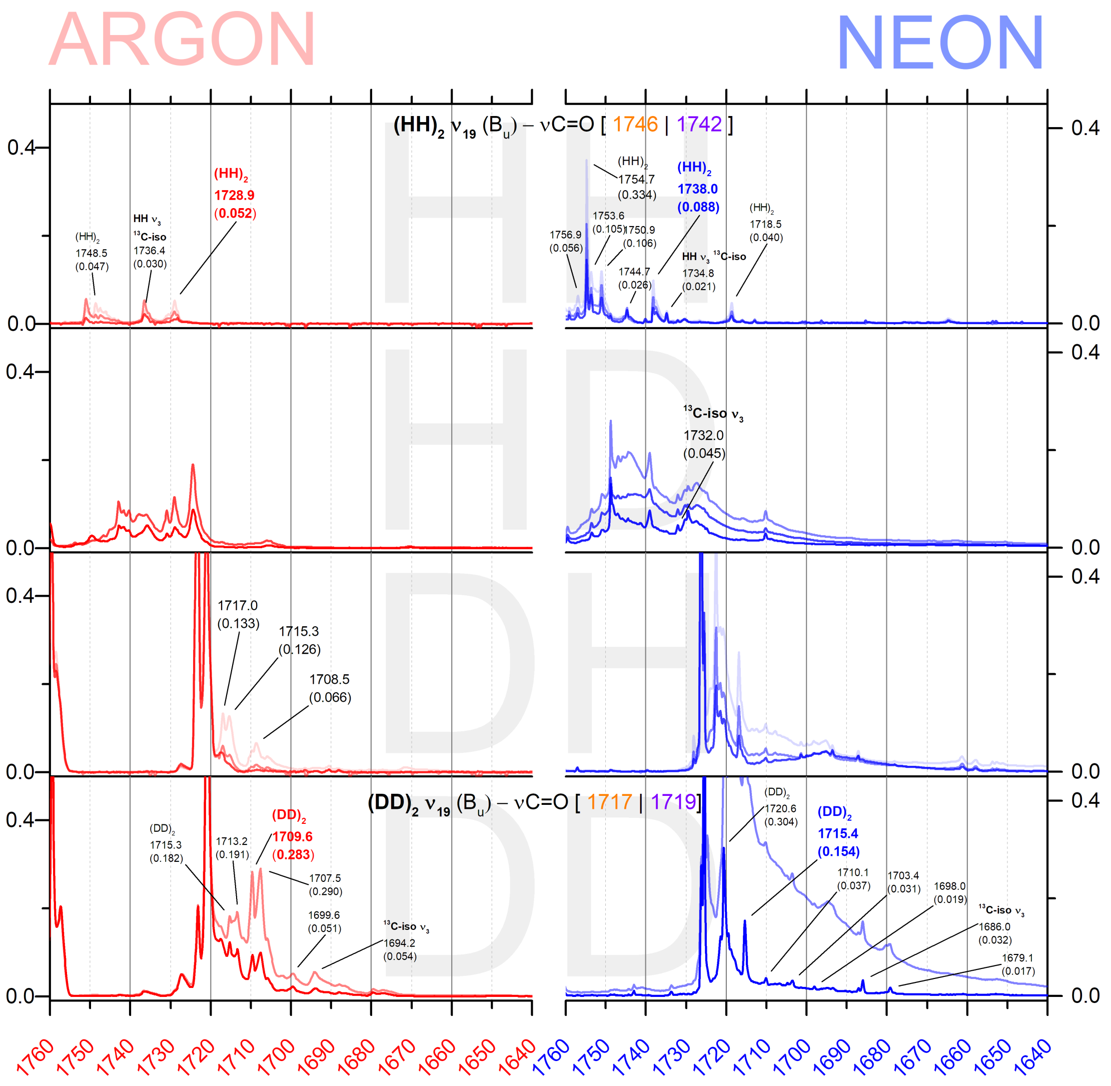

- The νC=O stretching vibration of the formic acid dimer **(HH)$_2$** is anticipated to occur at 1748 and 1728 cm^{-1} in an argon MI-FTIR spectrum, with the lower one corresponding to the cyclic dimer, as documented by Marushkevich et al.[8] In the **HH** sample, we also observe these vibrational bands both in argon (1748.5 and 1728.9 cm^{-1}) and neon (1754.7 and 1738.0 cm^{-1}) MI-FTIR spectra, of which we assign the lower one to the cyclic **(HH)$_2$** dimer.
- In the **HD** and **DH** samples, we can also expect the corresponding **(HD)$_2$** and **(DH)$_2$** dimer band of the νC=O vibration. An assignment is not done here.
- In the **DD** sample, we observe the dimer bands both in both in argon (1715.3 and 1709.6 cm^{-1}) and neon (1720.6 and 1715.4 cm^{-1}) MI-FTIR spectra, of which we assign the lower one to the cyclic **(DD)$_2$** dimer.
- The decrease in band intensity with increasing dilution supports the assignment to the dimers. In contrast, there are some other bands in the same spectral region, which maintain consistent intensities across varying dilution conditions. Some are due to ^{13}C isotopes, others probably due to combination bands (see Nejad: **HH** $v6 + v7$ at 1726, **HD** $v6 + v7$ at 1730, **DD** $v6 + v7$ at 1720).

1.6 The δ_{ip}CH, δ_{ip}COH, and νC−Oregion [1410–1080 cm^{-1}]

1.6.1 δ_{ip}CH of HH, HD & δ_{ip}COH of (HH)$_2$ [1410–1340 cm^{-1}]

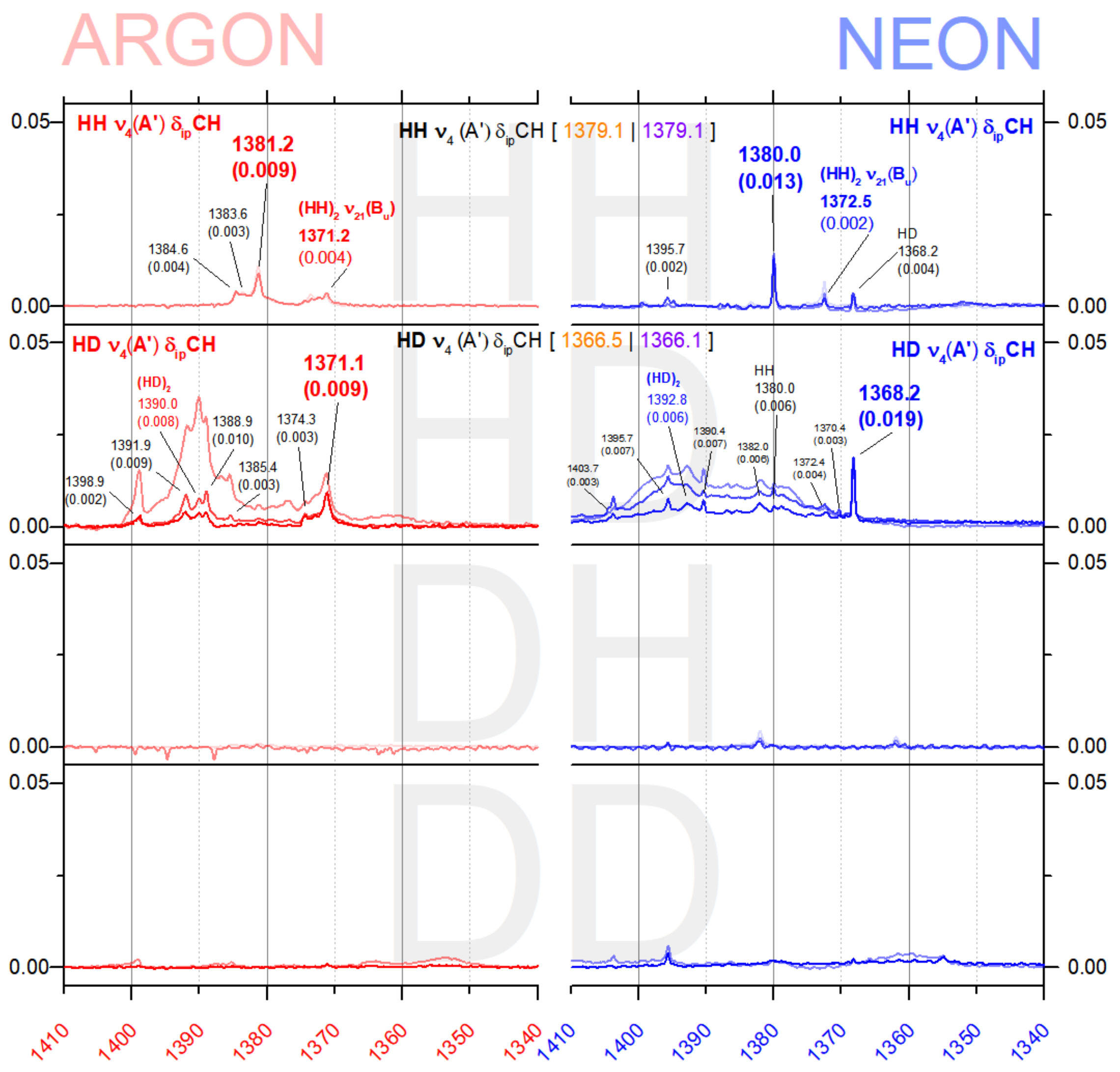

- Within the spectral range from 1410 to 1340 cm^{-1}, the δ_{ip}CH vibrational mode of the *trans*-formic acid monomer is expected.
- In the **HH** sample, we observe the vibration both in argon (1381.2 cm^{-1}) and neon (1380.0 cm^{-1}) MI-FTIR spectra, which is in good agreement with gas phase (1379.1 cm^{-1}) reference and VCI (1379.1 cm^{-1}) calculation. In the **HD** sample, the δ_{ip}CH vibration is at lower wavenumbers, both in argon (1371.1 cm^{-1}) and neon (1368.2 cm^{-1}) MI-FTIR spectra, and also in good agreement with gas phase (1366.5 cm^{-1}) reference and VCI (1366.1 cm^{-1}) calculation.
- In accordance with jet-expansion experiments[2], the δ_{ip}COHvibrational mode is observed in the cyclic, non-polar dimer of **(HH)$_2$**, in both argon (1317.2 cm^{-1}) and neon (1372.5 cm^{-1}). Also in the **HD** sample, we observe the respective band of the **(HD)$_2$** dimer in both argon (1390.0 cm^{-1}) and neon (1392.2 cm^{-1}).

1.6.2 δ_{ip}CH of $(HH)_2$ [1420–1390 cm^{-1}]

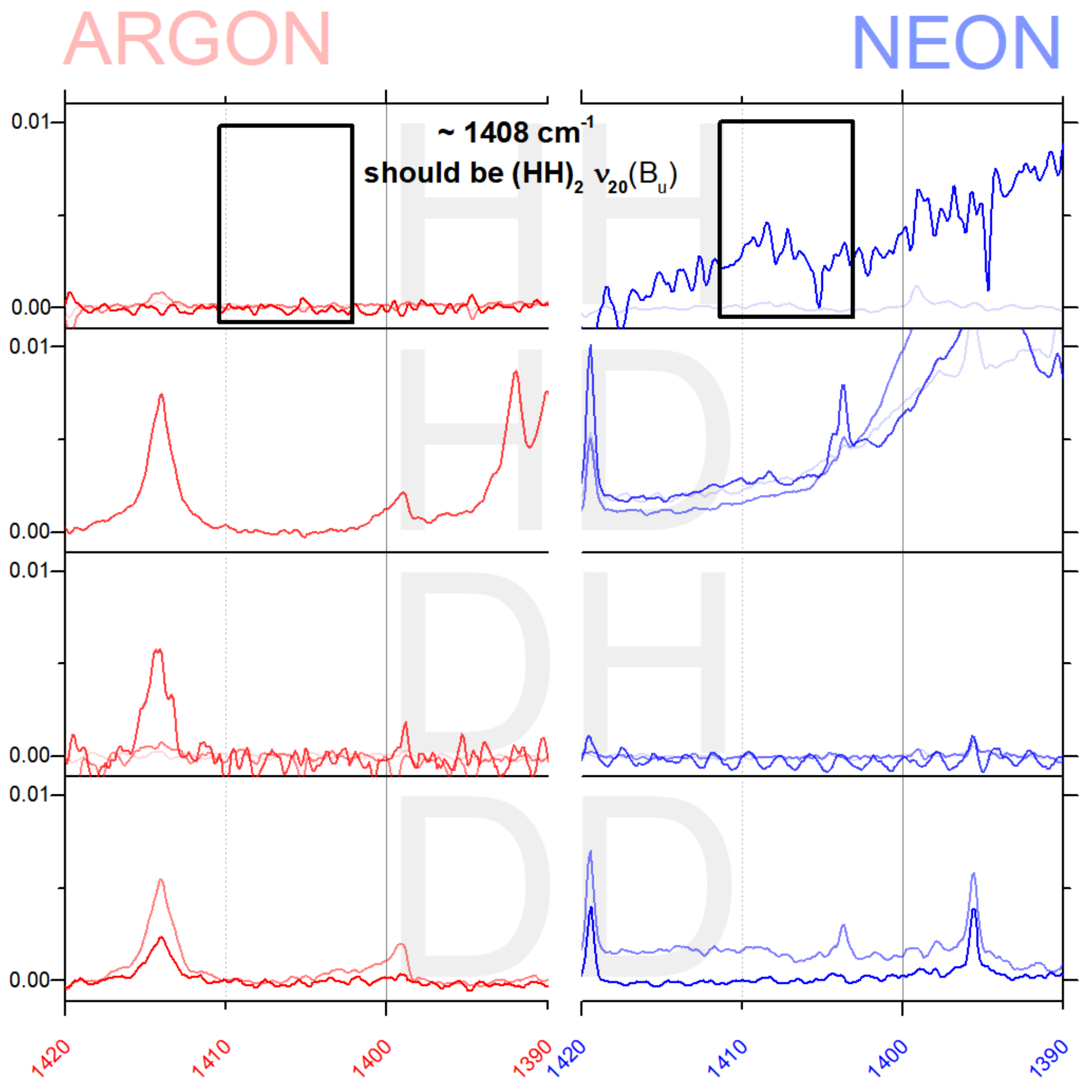

- In the literature, the δ_{ip}CH mode of the dimer has been assigned by Gantenberg et al.[9] to 1445 cm^{-1} in argon MI-FTIR spectra. This band is not observed in the present work. In jet-expansion experiments, the same mode was assigned to 1454 cm^{-1} by Georges et al.[10] and later to 1408 cm^{-1} by Nejad[2]. The latter assignment is in best agreement with the present VCI calculations, which predict a value of 1404 cm^{-1}. We expect the band to occur around 1408 cm^{-1} in the MI-FTIR spectra as well.
- The δ_{ip}CH vibration of the monomer has very low intensity, and the corresponding dimer band is expected to be even weaker as the equilibrium abundance of the dimer is significantly lower than that of the monomer. Consequently, observation of this band by IR spectroscopy is very difficult. In the present MI-FTIR spectra, only very weak features are observed that cannot be distinguished from the background noise.

1.6.3 δ_{ip}COH of HH (& resonance), νC–O of $(HH)_2$, $(DD)_2$ [1310–1190 cm^{-1}]

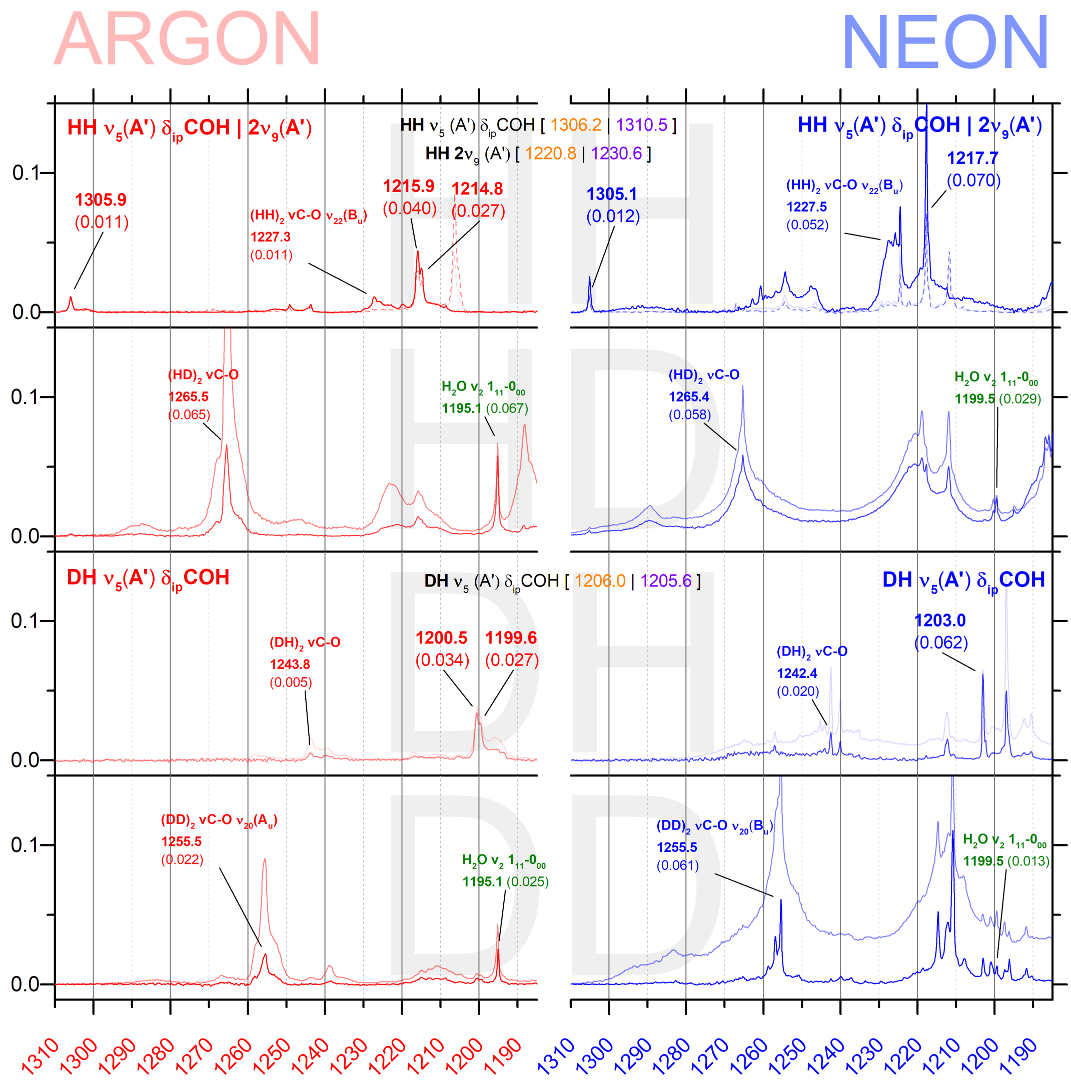

- In the **HH** sample, we observe the δ_{ip}COH fundamental in resonance with the overtone $2\nu_9$, both in argon (1305.9 / 1215.9 cm^{-1}) and neon (1305.1 / 1217.7 cm^{-1}) MI-FTIR spectra, and in good agreement with gas phase (1306.2/1220.8 cm^{-1}) reference and VCI (1310.5/1230.6 cm^{-1}) calculations. We furthermore observe the strC O vibration of the $(HH)_2$ dimer in argon (1227.3 cm^{-1}) and neon (1227.5 cm^{-1}) MI-FTIR spectra.
- In the **HD** sample, we observe the νC–O vibration of the $(HD)_2$ dimer.
- In the **DH** sample, we observe the δ_{ip}COH fundamental only as one band in argon (1200.1 cm^{-1}) and neon (1203.0 cm^{-1}) MI-FTIR spectra. This agrees with gas phase (1206.0 cm^{-1}) reference data, where also only one band is listed. However, we would expect a resonance from the VCI (1298.4/1205.6 cm^{-1}) calculation, similar to the **HH** species, which is not observed. We furthermore observe the νC–O vibration of the $(DH)_2$ dimer in argon and neon MI-FTIR spectra.
- In the **DD** sample, we observe the νC–O vibration of the $(DD)_2$ dimer both in argon (1255.5 cm^{-1}) and neon (1255.5 cm^{-1}) MI-FTIR spectra.

1.6.4 νC$-$O region [1190–1080 cm^{-1}]

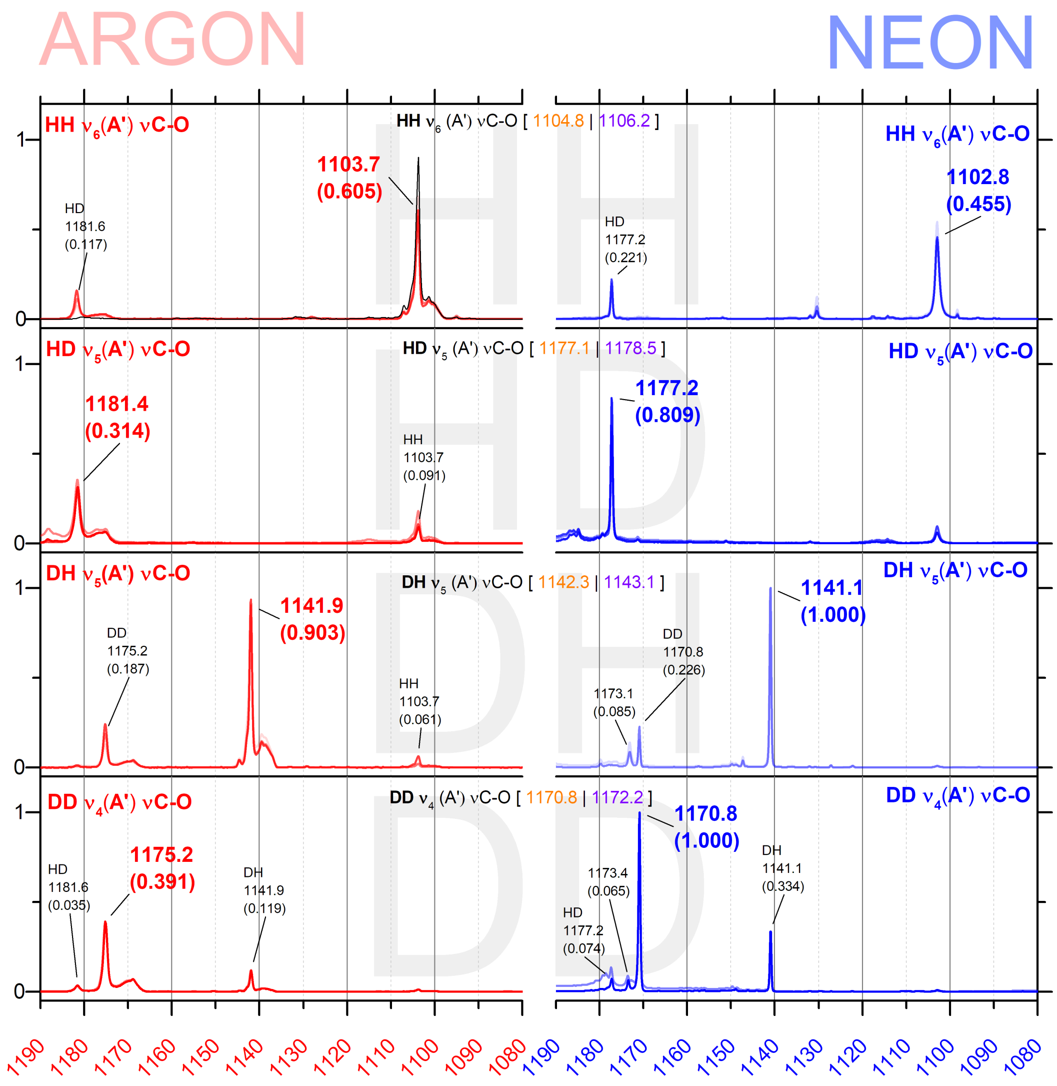

- The spectral region shown includes the νC$-$O vibration of the monomer, which is observed for all considered isotopocules of formic acid. The agreement with literature data and VCI calculations is very good and supports the assignment. Significant mutual contamination is observed between **DH** and **DD**, as well as between **HH** and **HD**.

1.7 The δ_{oop}CH region [1080–840 cm^{-1}]

1.7.1 δ_{oop}CH of $(HH)_2$ & δ_{ip}COD of $(DD)_2$ [1080–1050 cm^{-1}]

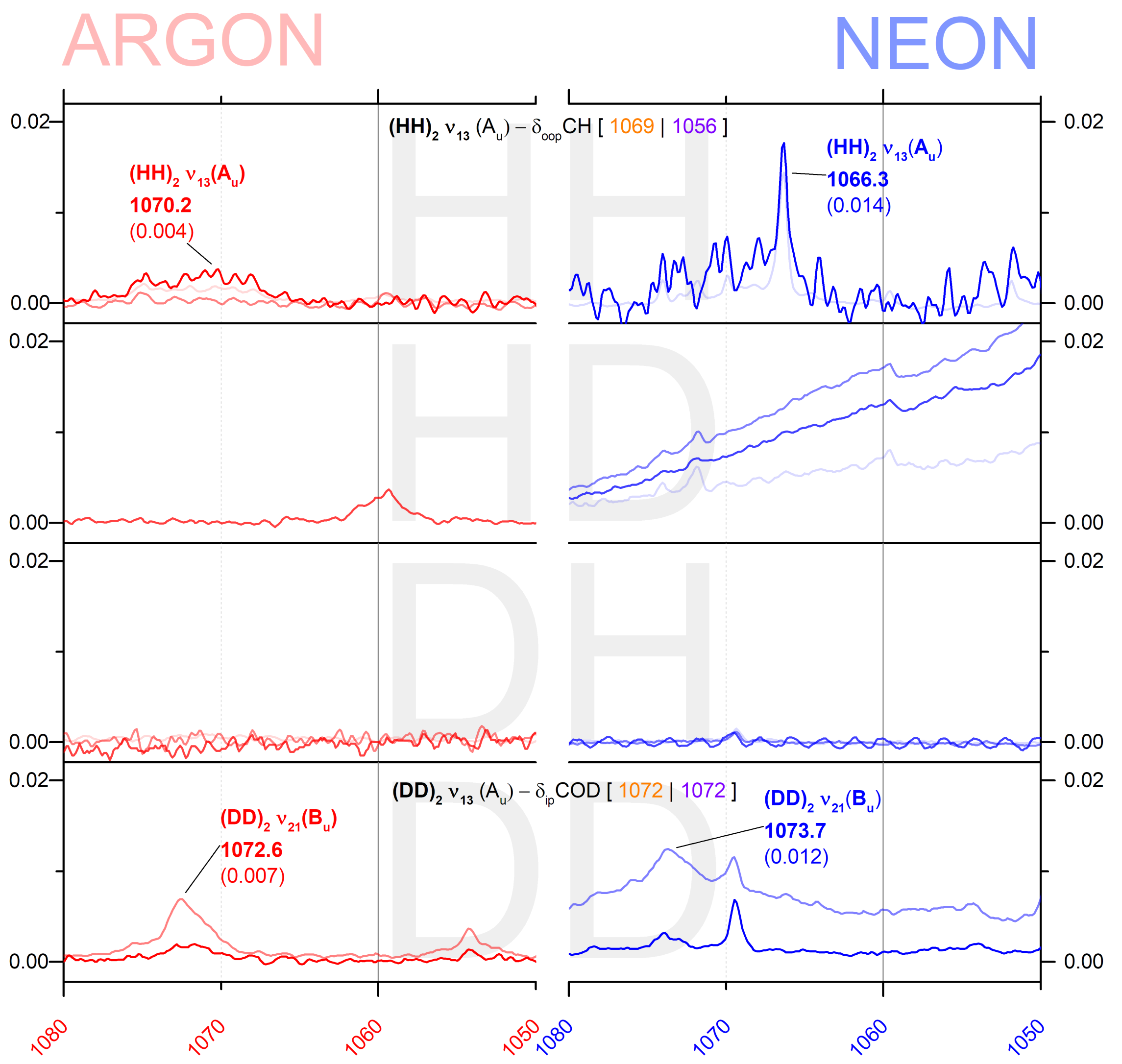

- In the spectral region shown, the δ_{oop}CH vibration of **$(HH)_2$** is expected. In the **HH** sample, we can tentatively assign some weak bands in both argon (1070.2 cm^{-1}) and neon (1066.3 cm^{-1}) MI-FTIR spectra.
- In the **HD** spectrum, similar features are likely present and may also correspond to the δ_{oop}CH vibration of **$(HH)_2$**. However, a detailed analysis of these dimer species is beyond the scope of the present work.
- In the **DD** spectrum, a band can be assigned to the δ_{ip}**COD** vibration of the **$(DD)_2$** dimer.
- The corresponding δ_{oop}CH and δ_{ip}**COD** vibrations of the respective monomer species are shown in Figure 1.7.2.

1.7.2 δ_{oop}CH of HH, HD & δ_{ip}COD of DD [1080–1010 cm^{-1}]

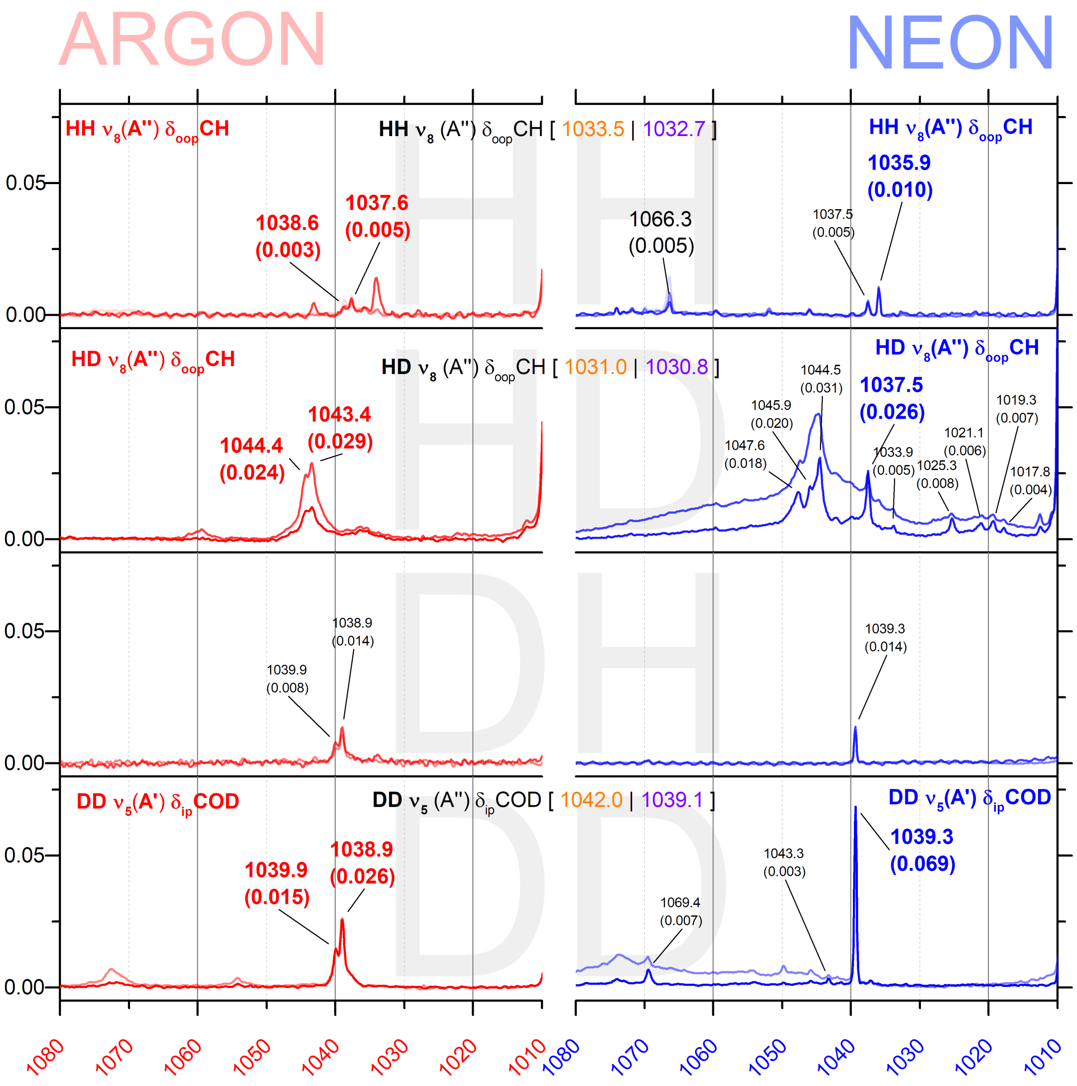

- The spectral region contains the δ_{oop}CH vibration of **HH** and **HD**. Similar to the other CH-related vibrations, the intensity is comparatively low.
- For **HH**, the bands are observed in both argon (1038.6, 1037.6 cm^{-1}) and neon (1035.5 cm^{-1}) MI-FTIR spectra and are in good agreement with gas phase (1033.5 cm^{-1}) reference data and VCI (1032.7 cm^{-1}) calculations.
- For **HD**, the bands are observed in both argon (1044.4, 1043.4 cm^{-1}) and neon (1037.5 cm^{-1}) MI-FTIR spectra and are in good agreement with gas phase (1031.0 cm^{-1}) reference data and VCI (1030.8 cm^{-1}) calculations.
- The corresponding δ_{oop}CD vibrations of **DH** and **DD** occur at significantly lower wavenumbers (cf. Figure 1.7.5).
- In the spectra of the **DD** sample, a relatively strong band is observed that can be assigned to the δ_{ip}**COD** vibration in argon (1039.9, 1038.9 cm^{-1}) and neon (1039.3 cm^{-1}) MI-FTIR spectra. This assignment is also in good agreement with gas phase (1042.0 cm^{-1}) reference data and VCI (1039.1 cm^{-1}) calculations.

1.7.3 δ_{ip}COD of HD & δ_{ip}CD of DH, DD, and $(DD)_2$ [1010–940 cm^{-1}]

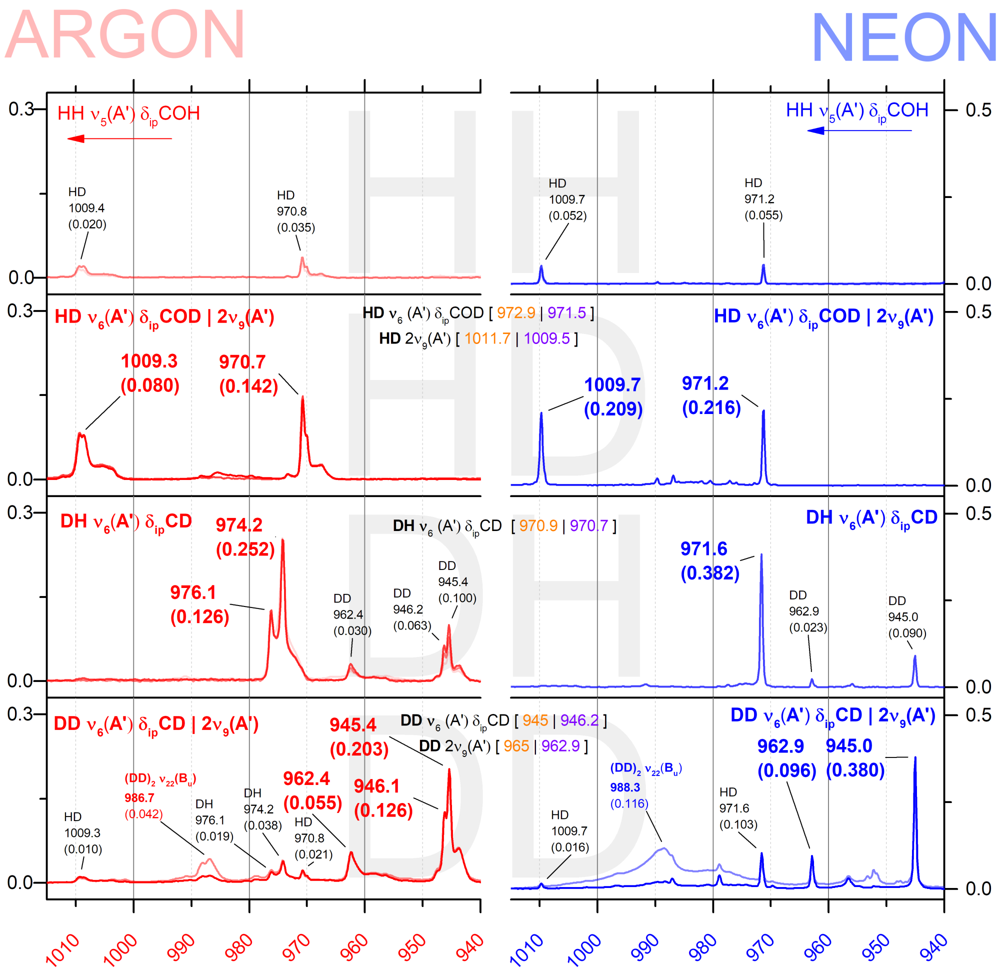

- In the spectra of the **HH** sample, only the δ_{ip}COD vibration of **HD** (i.e., the ν_6 / $2\nu_9$ resonance of **HD**) is observed as a result of isotopic exchange. The corresponding δ_{ip}COH vibration (and its resonance partner) of **HH** occurs at significantly higher wavenumbers (cf. Figure 1.6.3).
- In the spectra of the **HD** sample, the δ_{ip}COD vibration (ν_6) is observed in resonance with the $2\nu_9$ overtone. The corresponding bands appear in both argon (1009.3/970.7 cm^{-1}) and neon (1009.7/971.2 cm^{-1}) MI-FTIR spectra and are in good agreement with gas phase (1011.7/972.9 cm^{-1}) reference data and VCI calculations.
- For the **DH** sample, a distinct band in argon (974.1 cm^{-1}) (with splitting) and neon (971.6 cm^{-1}) MI-FTIR spectra can be assigned to the δ_{ip}CD vibration, in good agreement with gas phase (970.9 cm^{-1}) reference and VCI (970.7 cm^{-1}) calculation. Additional weaker features arise from isotopic exchange. The corresponding δ_{ip}COH vibration of **DH** is again located at higher wavenumbers (cf. Figure 1.6.3).
- In the spectra of the **DD** sample, a pattern of bands is observed that can be assigned to the δ_{ip}CD vibration (ν_6) in resonance with the $2\nu_9$ overtone. These bands are present in both argon (962.4 / 945.4 cm^{-1}) and neon (962.9/945.0 cm^{-1}) MI-FTIR spectra and agree well with gas phase (965/945 cm^{-1}) reference data and VCI (962.9/946.2 cm^{-1}) calculations. The corresponding δ_{ip}COD vibration of **DD** occurs at higher wavenumbers (cf. Figure 1.7.2), and additional bands can be attributed to impurities of **HD** and **DH**.
- For the **DD** sample, an additional band in argon (986.7 cm^{-1}) and neon (988.3 cm^{-1}) MI-FTIR spectra can be assigned to the δ_{ip}CD vibration of the $(DD)_2$ dimer.

1.7.4 δ_{oop}COH of (HH)$_2$, δ_{ip}CD of (DD)$_2$ [1000–920 cm^{-1}]

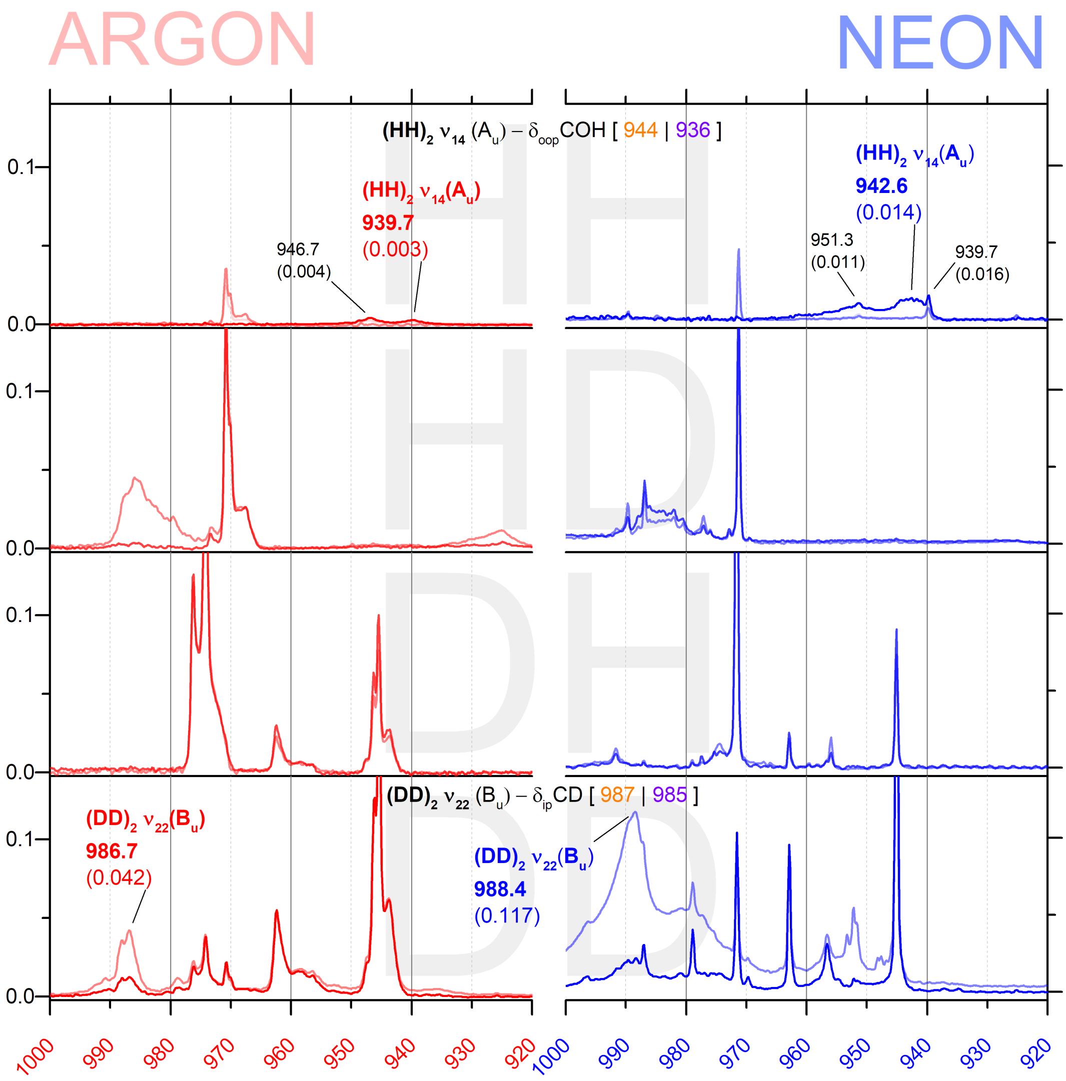

- For the **HH** sample, we can assign the δ_{oop}COH of **(HH)$_2$**, in argon (939.7 cm^{-1}) and neon (942.6 cm^{-1}) MI-FTIR spectra.
- For the **DD** sample, we show again the δ_{ip}CD vibration of the **(DD)$_2$** dimer, as observed in argon (986.7 cm^{-1}) and neon (988.3 cm^{-1}) MI-FTIR spectra.

1.7.5 δ_{oop}CD of DH, DD [920–840 cm^{-1}]

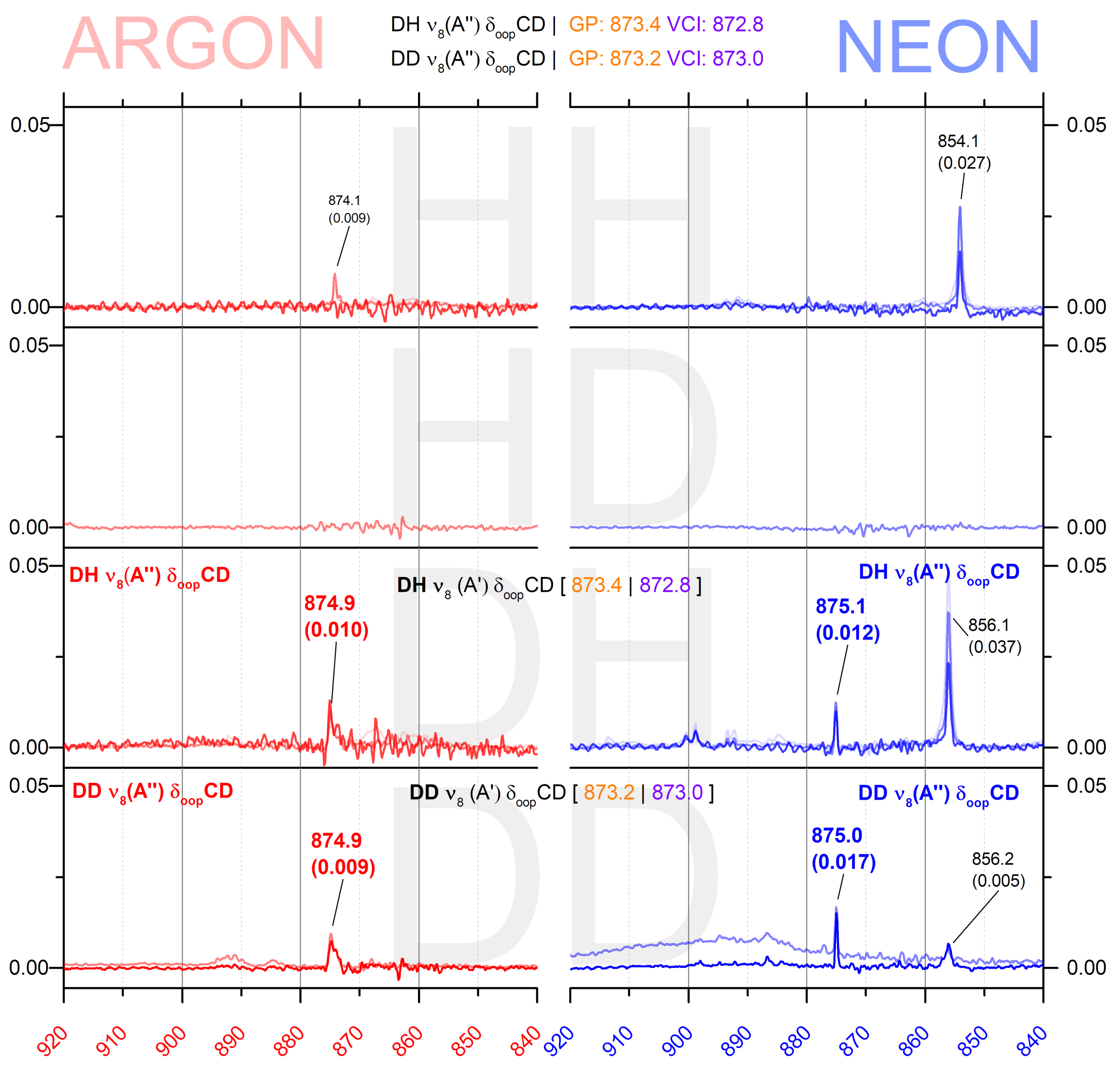

- The spectral region contains the δ_{oop}CD vibration of **DH** and **DD**, which are almost identical in vibrational frequency. The intensity of these bands is, similar to the corresponding δ_{oop}CH vibrations in **HH** and **HD**, very low.
- In both the **DH** and **DD** sample, the δ_{oop}CD vibration can be identified in both argon (874.9 and 874.9 cm^{-1}) and neon (875.1 and 875.0 cm^{-1}) MI-FTIR spectra and show good agreement with gas phase (873.4 and 873.2 cm^{-1}) reference data and VCI (872.8 and 873.0 cm^{-1}) calculations.
- The corresponding δ_{oop}CH vibrations of **HH** and **HD** occur at significantly higher wavenumbers (cf. Figure 1.7.2).
- In the neon MI-FTIR spectra, an additional band is observed at 854 cm^{-1} in the **HH** spectrum and at 856 cm^{-1} in the **DH** and **DD** spectra, which remains unassigned in the present work. The intensity of this feature is particularly pronounced in the **HH** and **DH** spectra, and no corresponding band is predicted by the present VCI calculations.

1.7.6 δ_{oop}CD of (DD)$_2$ [920–880 cm^{-1}]

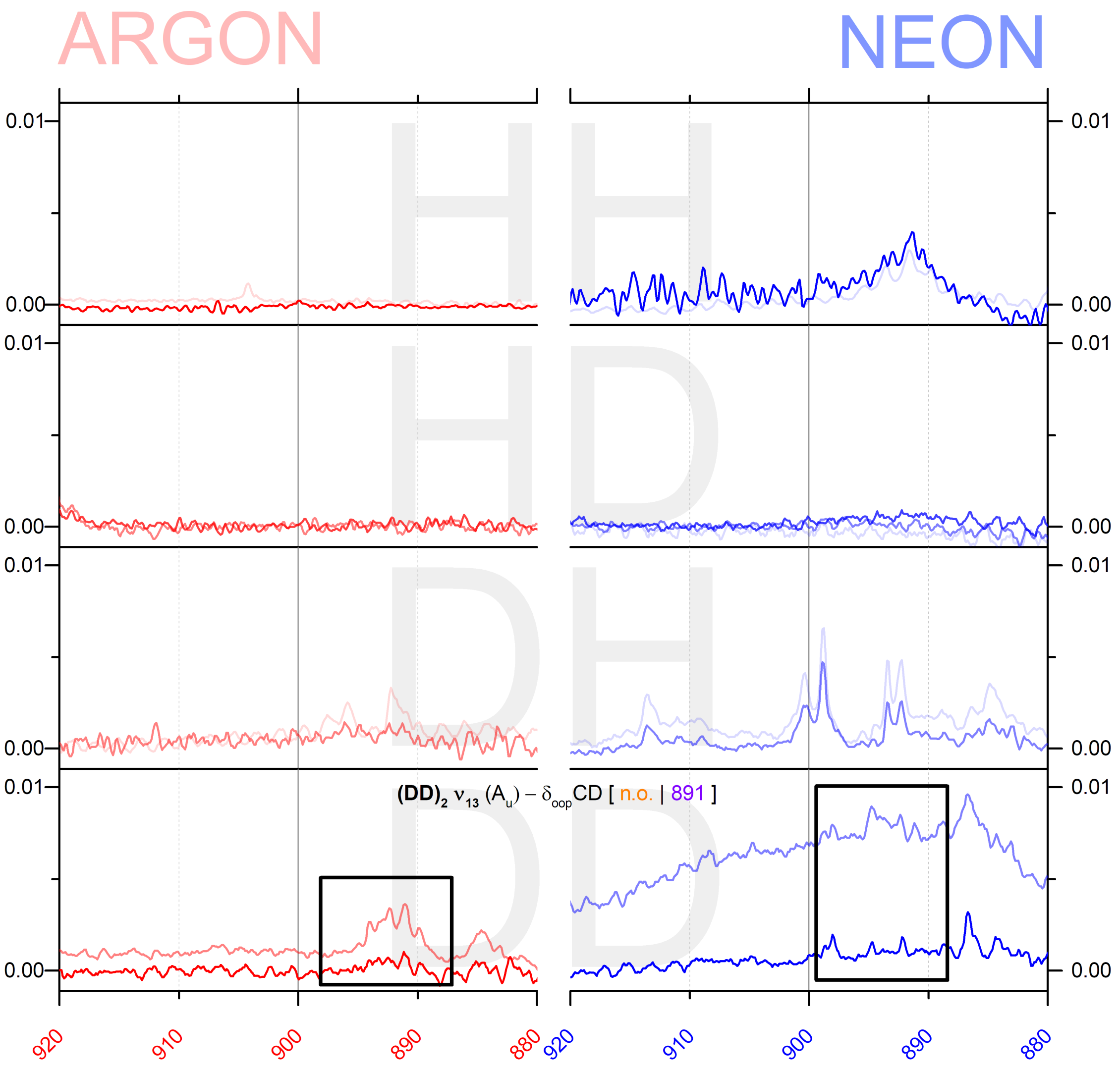

- The δ_{oop}CD vibration of **(DD)$_2$** is predicted by VCI calculations to appear in this spectral region. However, its intensity is expected to be very low. This likely explains why no gas-phase or matrix-isolation reference data for this band are available.
- In the argon MI-FTIR spectra, a weak feature is observed in this region, but it is close to the noise level, preventing a reliable assignment. In the neon MI-FTIR spectra, the signal quality is improved and several sharp, low-intensity bands are observed. However, an unambiguous assignment remains difficult, as it is unclear which feature corresponds to the δ_{oop}CD vibration of **(DD)$_2$**.
- While the present data may indicate a first observation of the δ_{oop}CD vibration of **(DD)$_2$**, further investigation is required to confirm this assignment. Therefore, a definitive assignment is not made here.

1.8 Dimer region [840–650 cm^{-1}]

1.8.1 The "empty" spectrum [840–740 cm^{-1}]

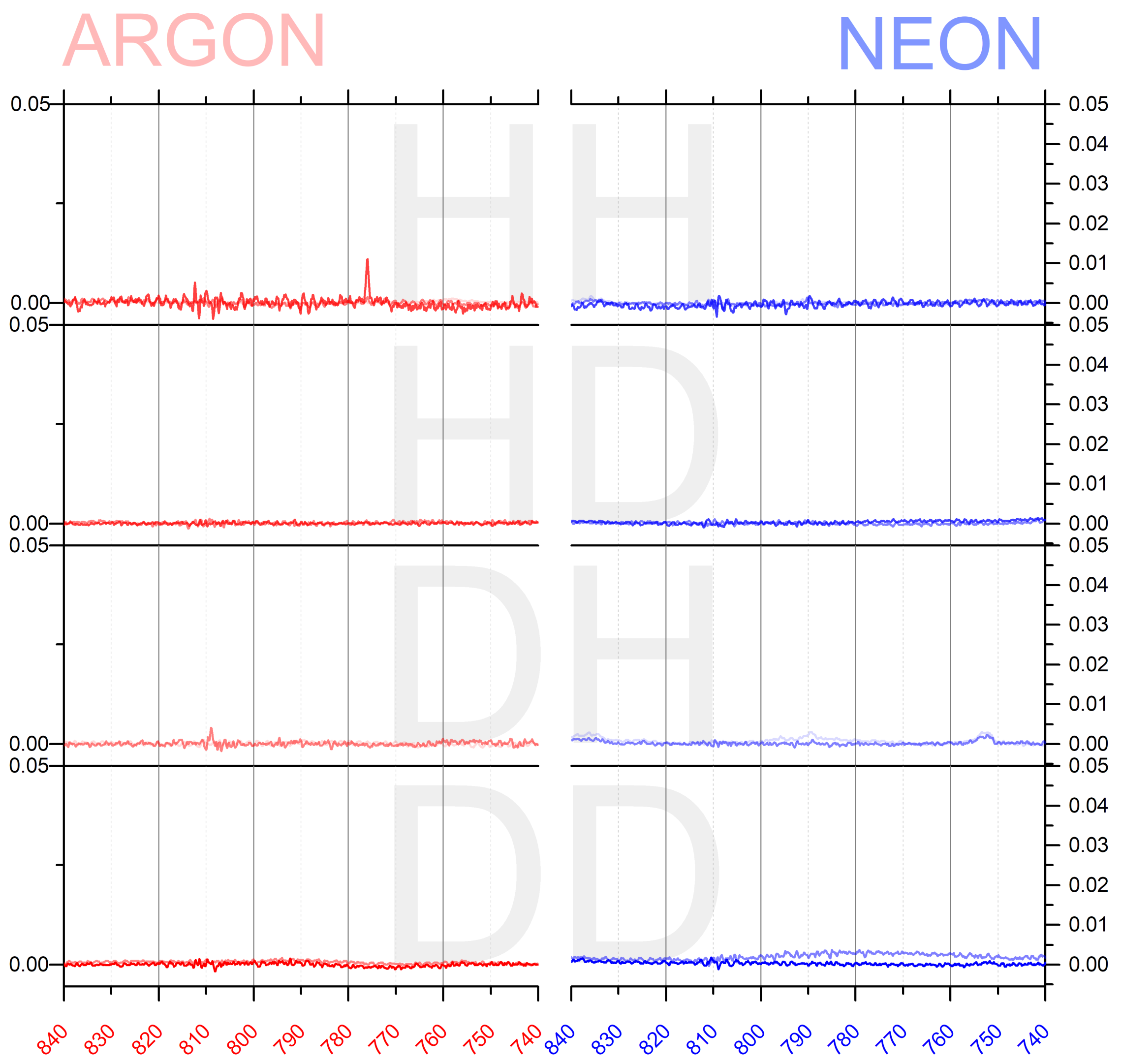

- Although some very weak signals are present, the spectral region shown here can be regarded as effectively empty, i.e., no bands are expected or observed that can be assigned to formic acid or its isotopocules.

1.8.2 δ_{ip}OCO of (HH)$_2$ & δ_{oop}COD of (DD)$_2$ [740–680 cm^{-1}]

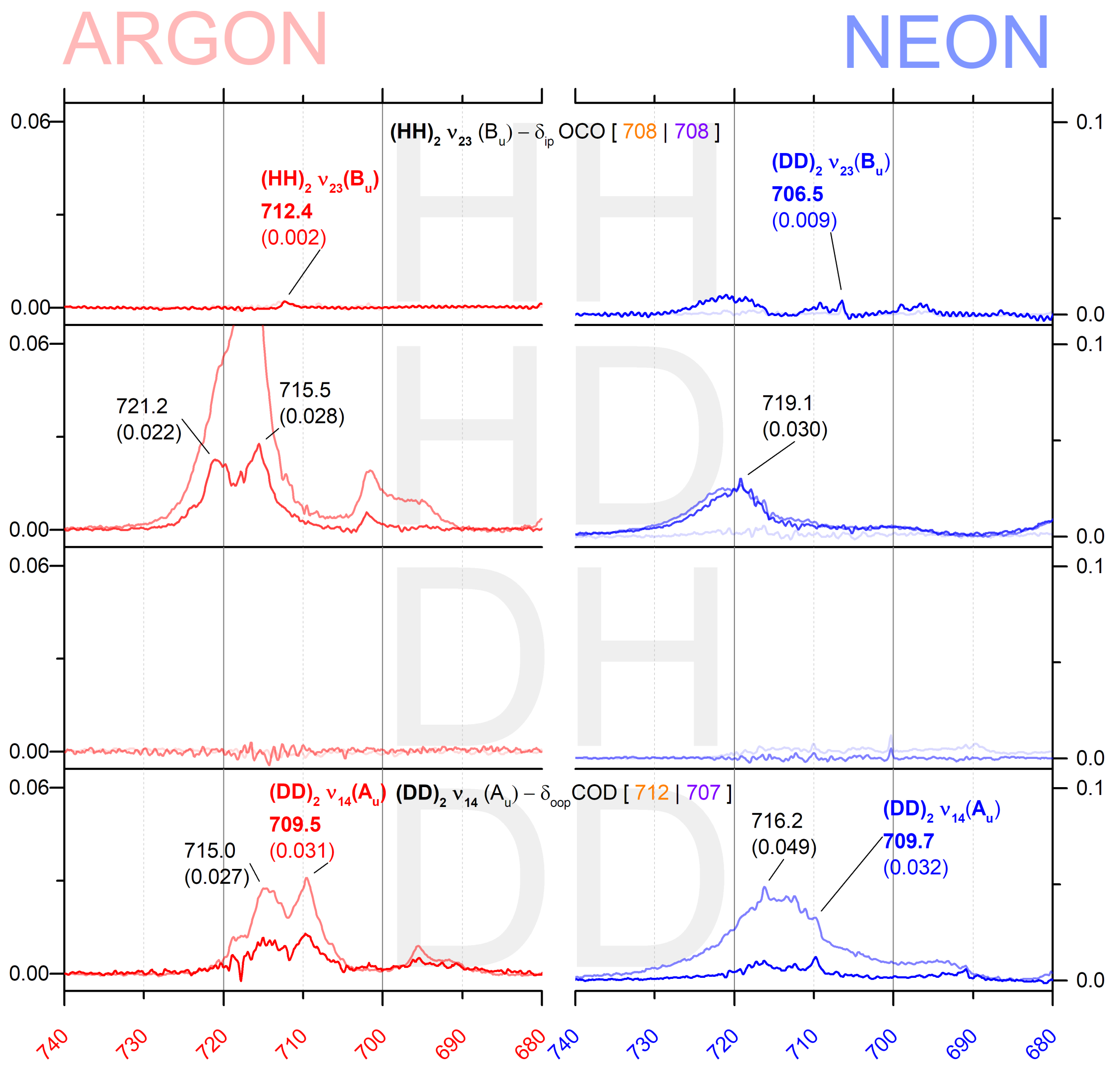

- In the **HH** sample, we can assign the δ_{ip}OCO vibration of **(HH)$_2$** in argon (712.4 cm^{-1}) and neon (706.5 cm^{-1}) MI-FTIR spectra.
- In the **DD** sample, we can assign the δ_{oop}COD vibration of **(DD)$_2$** in argon (709.5 cm^{-1}) and neon (709.5 cm^{-1}) MI-FTIR spectra.

1.8.3 δ_{ip}OCO of (DD)$_2$ & CO_2 ν_2 [740–650 cm^{-1}]

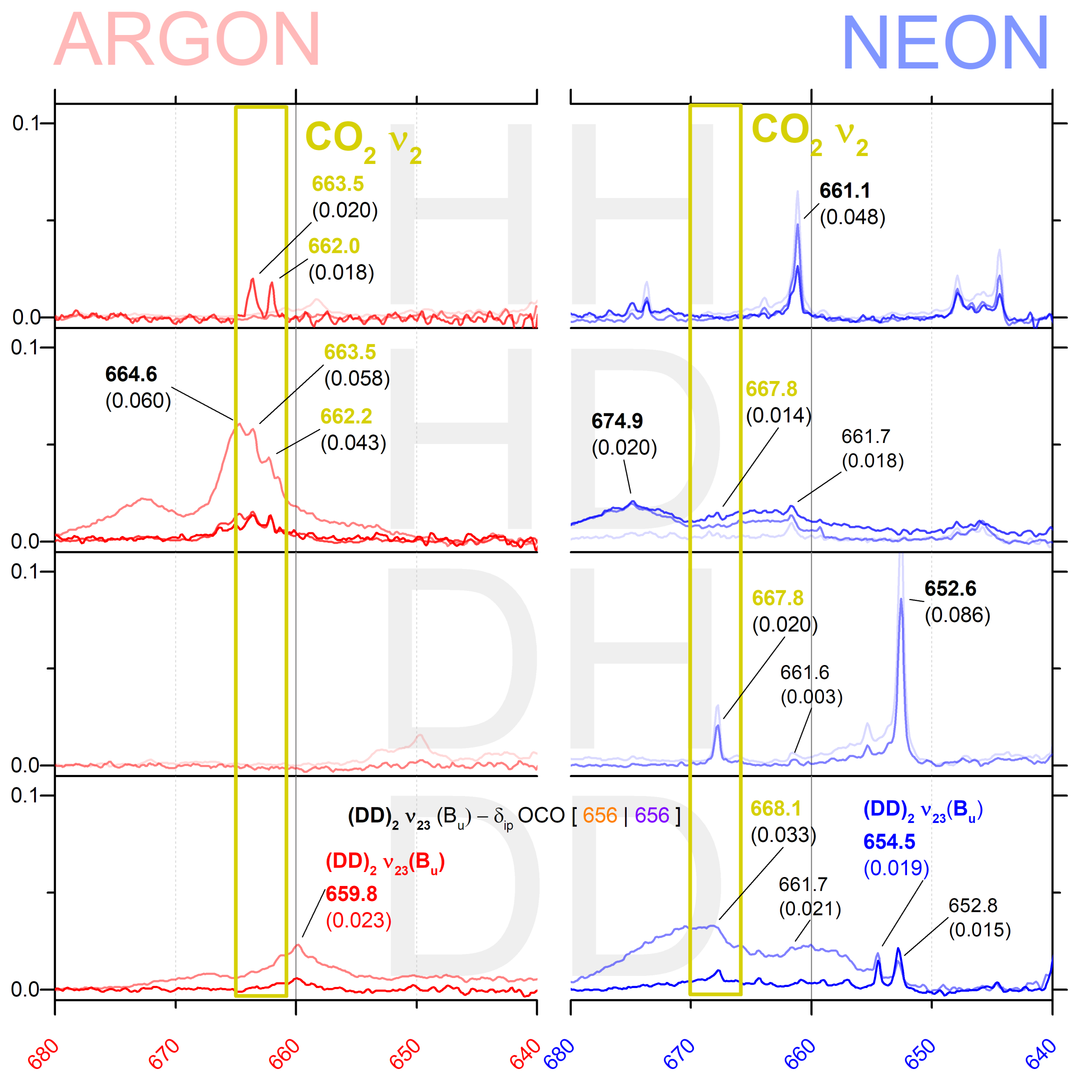

- In this spectral region, the ν_2 bending vibration of CO_2 is observed in almost all samples.
- In close proximity to this band, the δ_{ip}OCO vibration of the **(DD)$_2$** dimer is also expected. In the **DD** sample, each one band in argon (659.8 cm^{-1}) and neon (654.5 cm^{-1}) MI-FTIR can be assigned. However, the assignment is tentative in argon MI-FTIR spectra due to low intensity.
- In all samples, several additional bands are observed that cannot be assigned either to monomer vibrational modes of the respective formic acid isotopocules or to the CO_2 impurity. Further analysis of dimers of **HD** and **DH** may be required to fully elucidate these features.

1.9 The δ_{oop}COH & δ_{ip}OCO region [650–500 cm^{-1}]

1.9.1 δ_{oop}COH & δ_{ip}OCO of HH & DH [650–600 cm^{-1}]

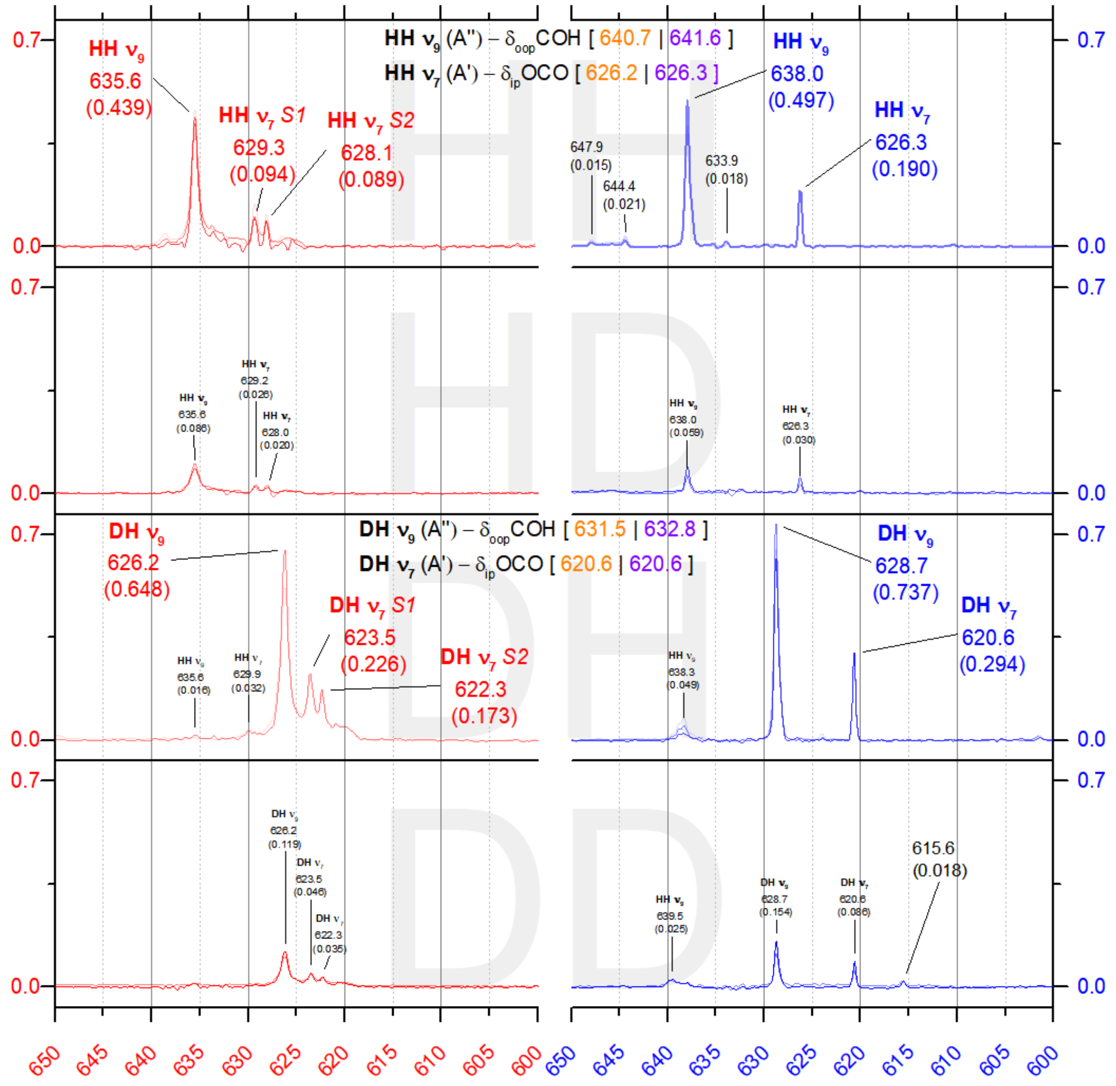

- In this spectral region, the δ_{oop}COH and δ_{ip}OCO vibrations are observed for the **HH** and **DH** samples in both argon and neon MI-FTIR spectra. In argon MI-FTIR spectra, matrix splitting is evident. In the **HD** and **DD** spectra, slight contamination by **HH** and **DH**, respectively, is observed due to isotopic exchange at the hydroxy group.
- The corresponding δ_{oop}OCO vibrations of **HD** and **DD** occur at lower wavenumbers (cf. Figure 1.9.2).
- The corresponding δ_{oop}COD vibrations of **HD** and **DD** are expected below 510 cm^{-1}. As this spectral region was not recorded in the present work, these vibrations are not assigned or further discussed.

1.9.2 δ_{oop}COH & δ_{ip}OCO of HD & DD [580–530 cm^{-1}]

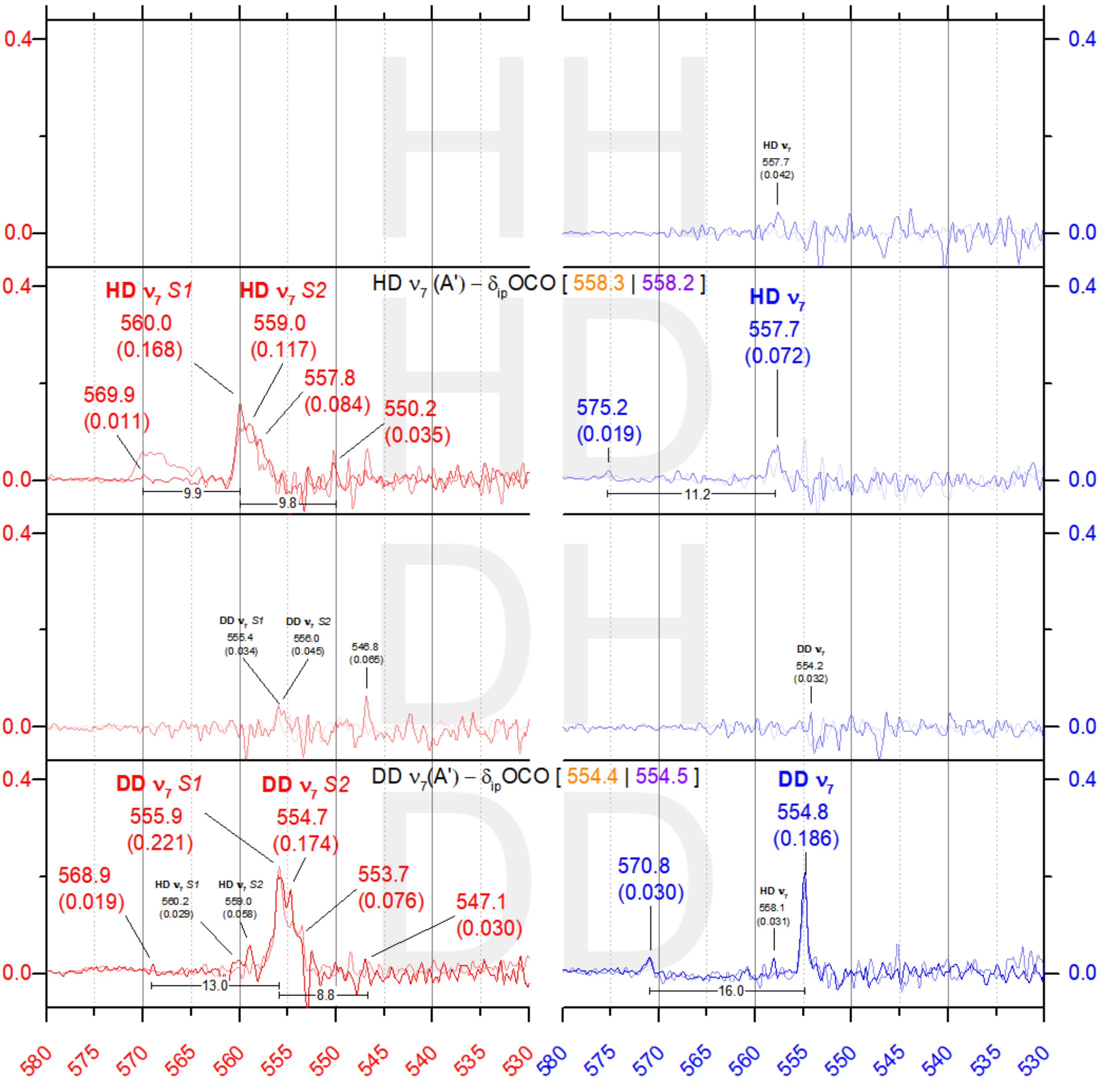

- In the spectral region shown, the δ_{ip}OCO vibration of the **HD** and **DD** species is observed in both argon and neon MI-FTIR spectra. The intensities are comparatively low, and the signal-to-noise ratio is insufficient to clearly resolve matrix splitting in argon MI-FTIR spectra. However, the presence of two trapping sites in argon matrices can be inferred.
- The corresponding δ_{oop}OCO vibrations of **HH** and **DH** occur at higher wavenumbers (cf. Figure 1.9.1).

1.10 Water and carbon dioxide contamination

1.10.1 D_2O and HDO [2840–2700 cm^{-1}]

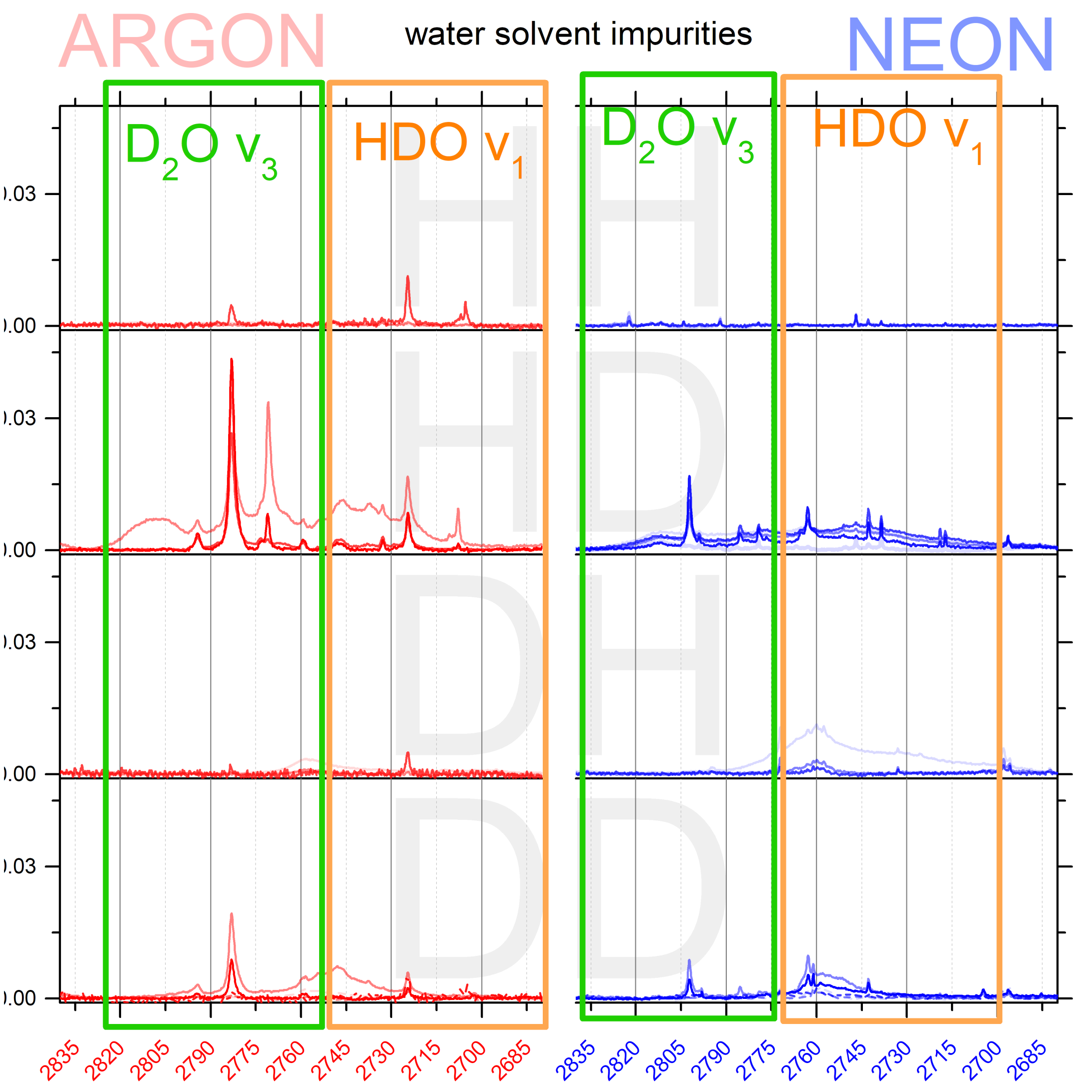

- In the spectral region from 2840 to 2700 cm^{-1}, vibrational bands corresponding to ν_3 of $\mathbf{D_2O}$ and ν_1 of **HDO** are observed, originating from residual water isotopocules trapped in the matrix.
- These water-related features arise from trace impurities that persist when the freeze–thaw purification is not performed with optimal efficiency.

1.10.2 CO_2 [2360–2220 cm^{-1}]

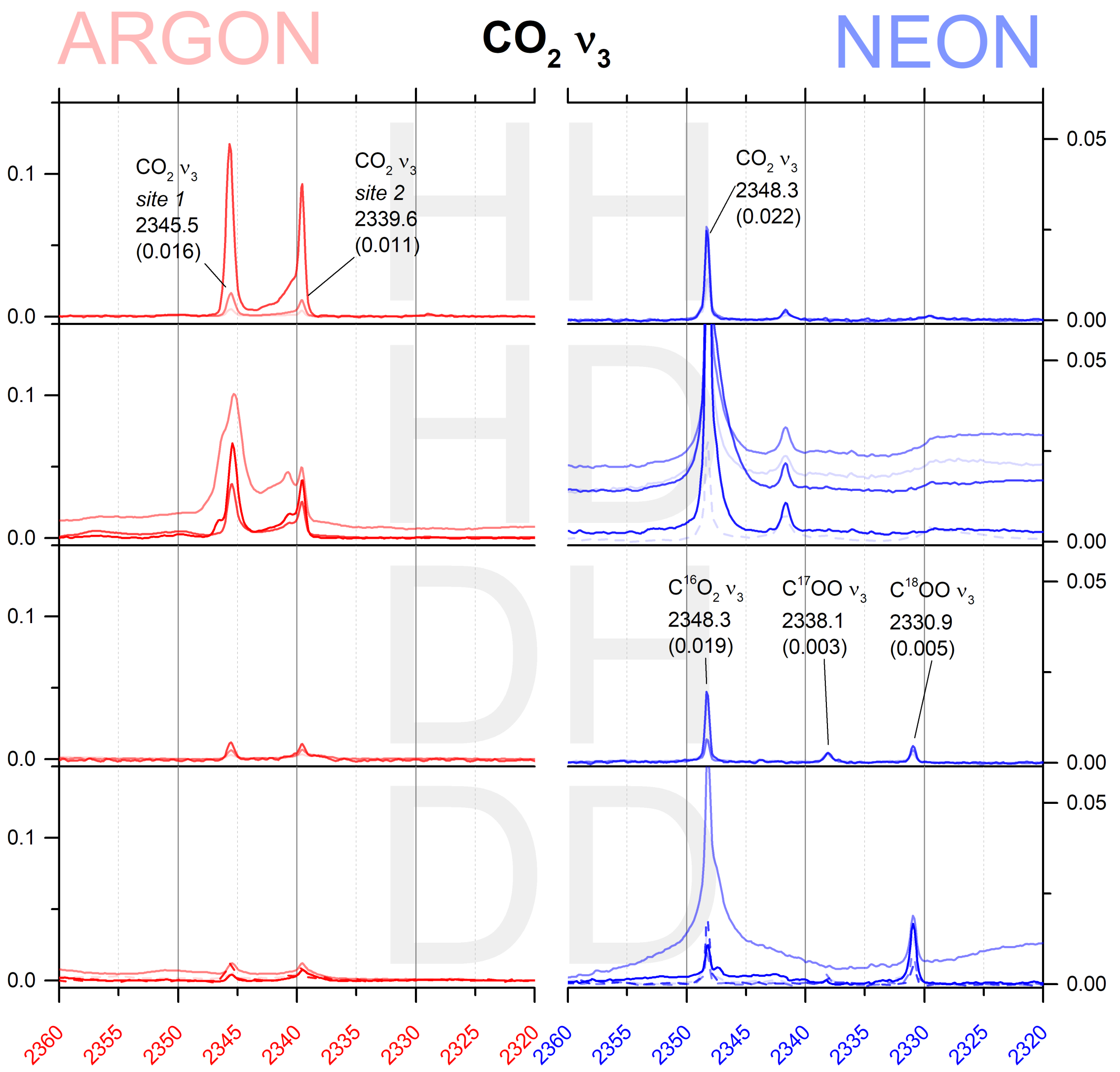

- In the spectral region from 2360 to 2220 cm^{-1}, the characteristic asymmetric stretching vibration ν_3 of CO_2 is observed, which is consistently present in conventional matrix isolation FTIR spectra.
- The freeze–thaw procedure used to reduce residual CO_2 was not performed with equal effectiveness in all experiments, resulting in varying levels of CO_2 contamination in the affected spectra.
- In the **DH** sample, there is a significant amount of ^{13}**C**, ^{17}**O** isotopic contamination, but only in the neon MI-FTIR spectra under slow deposition. The origin of this is unknown. Further ν_3 of CO_2 isotopocules are observable in Figure 1.3.4.

1.10.3 HDO [1600–1400 cm^{-1}]

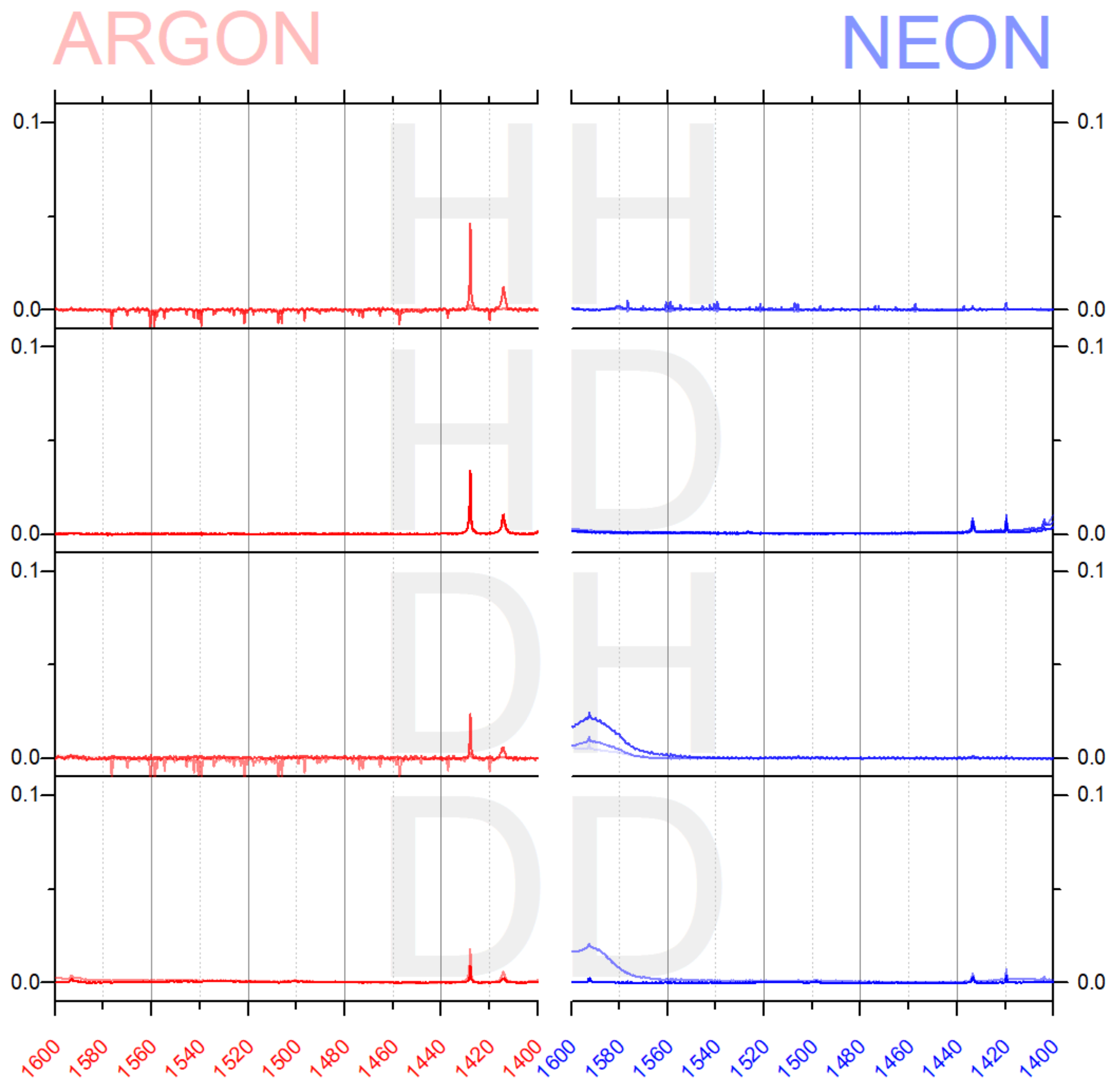

- MI-IR spectrum from 1600 to 1400 cm^{-1}, showing the bending vibration region of residual water, especially the bending of the HDO isotopocule.

2 Compilation of Vibrational Assignments and Reference Spectroscopic Data

Here we list our working tables containing all directly observed and assigned vibrational transitions in argon and neon matrices, together with their relative intensities. The respective data for the formic-acid monomers, also including reference data from previous matrix isolation experiments in argon and neon, as well as gas-phase experiments reported in the literature, are listed in Table S1 for **HCOOH**, Table S2 for **HCOOD**, Table S3 for **DCOOH**, and Table S4 for **DCOOD**. The respective data for the dimers **$(HCOOH)_2$** and **$(DCOOD)_2$** are shown in Table S5 and S6. The references are abbreviated as [AutYY], where Aut denotes the first three letters of the first author's surname and YY the last two digits of the publication year:

[Din26] Data from Dinu 2026, i.e., the present work.[11]

[Hen87] Data from Henderson 1987.[12]

[Rev94] Data from Reva 1994.[13]

[Lun94] Data from Lundell 1994.[14]

[Gan00] Data from Gantenberg 2000.[9]

[Maç03] Data from Maçôas 2003.[3]

[Red77] Data from Redington 1977.[15]

[Ber82] Data from Bertie 1982.[16]

[Ber86] Data from Bertie 1986.[17]

[Mar10] Data from Marushkevich 2010.[8]

[Fre02] Data from Freytes 2002.[5]

[Hal98] Data from Halupka 1998.[18]

[Ito08] Data from Ito 2008.[19]

[Ito15] Data from Ito 2015.[20]

[Mar87] Data from Maréchal 1987.[21]

[Wac87] Data from Wachs 1987.[22]

[Zie07] Data from Zielke 2007.[23]

[Geo04] Data from George 2004.[24]

[Gut08] Data from Gutberlet 2008.[25]

[Nej22] Data from Nejad 2022.[2]

Table S1: Calculated (VCI, VPT2) and experimental vibrational fundamentals, overtones, and combination bands (cm^{-1}) of the *trans*-formic acid monomer (**HCOOH**) in argon and neon matrices and in the gas phase. Relative intensities, normalized to the strongest band (100), are given in parentheses where available.

	Calculation		Argon matrix						Neon matrix		Gas phase					
Mode	VCI	VPT2	[Din26]	[Hen87]	[Rev94]	[Lun94]	[Gan00]	[Maç03]	[Din26]	[Red77]	[Red77] (comp.)	[Ber82]	[Mar10]	[Fre02]	[Nej22] (IR, comp.)	[Nej22] (Raman jet)
ν_1	3571.5 (8.7)	3568.3 (23.2)	3550.2 (19.1)	3550.6	3546 (27.2)	3550.4	3549.9 (22.5)	3549.35 (m)	3570.0 (22.3)	3569.4	3570	3568.9	3567	3570.5	3570.5	3570
ν_2	2940.3 (8.4)	2942.6 (6.0)	2954.5 (3.2)	2953.9	2950 (10.2)	2952.9	3066.5 (11.6)	2954.6 (m)	2938.5 (13.6)	2937.8	2943.8	2942	2941	2942.06	2942.06	2942
ν_3	1778.4 (100.0)	1778.8 (100.0)	1768.4 (100.0)	1768.1	1766 (81.8)	1767.2	1766.9 (98.3)	1768.05 (vs)	1774.2 (100.0)	1773.9	1776.2	1776.6	1775	1776.83	1776.83	1776
ν_4	1379.1 (0.9)	1378.2 (0.7)	1381.2 (0.9)	1381.1	1379 (1.3)	1381.0		1382.7 (w)	1380.0 (1.3)	1379.7	1387	1380.6		1380	1379.05	1379
ν_5	1310.5 (1.0)	1302.0 (2.3)	1305.9 (1.1)			1305.7		1305.7 (w)	1305.1 (1.2)						1306.2	1306
ν_6	1106.2 (86.0)	1105.6 (86.5)	1103.7 (60.5)	1104.4	1102 (79.8)	1103.4	1103.5 (100)	1103.55 (s)	1102.8 (45.5)	1102.8	1105.4	1103.8	1104	1104.85	1104.85	1104
ν_7	626.3 (12.2)	627.3 (12.2)	628.7 (18.3)	629.8	628 (21.8)	629.2		628.65 (m)	626.3 (19.0)	625.9	625.4	624.9	612	626.16	626.17	626
ν_8	1032.7 (0.8)	1033.0 (0.8)	1038.1 (0.8)	1037.5	1036 (1.0)	1037.4		1037.95 (w)	1035.9 (1.0)	1035.6	1033.4			1033.47	1033.47	1033
ν_9	641.6 (42.7)	636.3 (42.8)	635.6 (43.9)	636.4	635 (56.7)	635.2	635.1 (63.5)	635.4 (s)	638.0 (49.7)	637.6	641.8	642	640	640.72	640.73	
$2\nu_3$	3537.9 (1.3)	3538.9 (0.5)	3516.5 (0.7)					3519	3529.6 (1.6)			2564?[a]			3534	3534
$\nu_3 + \nu_6$	2879.6 (0.6)	2879.2 (0.7)	2866.2 (0.3)						2872.0 (0.4)					2876.6	2876.6	
$2\nu_9 + \nu_6$	2406.5 (0.3)	2396.1 (0.5)	2398.1 (0.3)					d.a.[b]	2397.0 (0.3)					d.a.[b]	2400.2	2400
$\nu_4 + \nu_5$	2328.6 (0.6)	2309.5 (0.0)	2328.9 (0.2)					d.a.[b]	2329.5 (0.1)					2298.6		2336
$2\nu_6$	2201.3 (1.5)	2199.6 (0.8)	2195.4 (0.5)					2196.1?[a]	2193.3 (0.7)					2196.3	2196.3	2197
$2\nu_9$	1230.6 (4.9)	1213.6 (3.1)	1215.9 (6.7)	1216.1	1214 (5.2)	1215.8	1215.2 (16.8)	1215.3	1217.7 (7.0)	1217.6	1223		1218	1223	1220.83	1220

The references are abbreviated as [AutYY], where Aut denotes the first three letters of the first author's surname and YY the last two digits of the publication year. Qualitative intensities: vs = very strong, s = strong, m = medium, w = weak; .

[a] Tentative assignment in the original work.

[b] d.a. = different assignment in the original work.

Table S2: Calculated (VCI, VPT2) and experimental vibrational fundamentals, overtones, and combination bands (cm^{-1}) of the *trans*-**HCOOD** monomer in argon and neon matrices and in the gas phase. Relative intensities, normalized to the strongest band (100), are given in parentheses where available.

	Calculation		Argon matrix				Neon matrix			Gas phase					
Mode	VCI	VPT2	[Din26]	[Hen87]	[Lun94]	[Mar10]	[Din26]	[Red77]	[Mar10]	[Red77] (comp.)	[Ber86]	[Mare87]	[Mar10] (comp.)	[Nej22] (IR, comp.)	[Nej22] (Raman jet)
ν_1	2939.4 (8.0)	2941.0 (4.0)	2941.4 (1.0)	2961.5		2961.2 (m,s)	2936.1 (4.1)	2939.4	2951.9 (m)	2944	2938/2942	2942	2942	2938	2938
ν_2	2632.4 (16.5)	2630.2 (16.6)	2619.2 (27.6)	2619.5	2616.6	2618.75 (s)	2632.1 (19.8)	2631.6	2631.5 (s)	2633.5	2631.4	2637	2631.4	2631.64	2631
ν_3	1775.5 (100.0)	1775.2 (100.0)	1765.9 (100.0)	1766	1759.3	1766.4 (vs)	1771.3 (100.0)	1770.9	1771 (vs)	1773	1773	1766	1773.6	1772.12	1772
ν_4	1366.1 (1.0)	1366.8 (1.0)	1371.1 (0.9)	1371.1		1372.7 (vw)	1368.2 (1.9)	1368.1	1367.9 (w)		1368		1368	1366.48	1365
ν_5	1178.5 (60.9)	1178.7 (60.1)	1181.4 (31.4)	1181.5	1180.9	1181.7 (vs)	1177.2 (80.9)	1177	1176.9 (vs)	1177.7	1177.9	1176	1177.1	1177.09	1176
ν_6	971.5 (11.8)	972.6 (12.0)	970.7 (14.2)	971.1	970.6	970.3 (m,s)	971.2 (21.6)	971.1	971.1 (s)	972.2	972	972	972.8	972.85	972
ν_7	558.2 (13.8)	558.6 (13.8)	559.5 (28.5)	560	559.8	559.2 (m,s)	557.7 (7.2)	555.9	557.9 (m)	556.3	560		560.2	558.27	558
ν_8	1030.8 (0.3)	1031.2 (0.4)	1043.9 (5.3)	1039.7	1038.8	1035.75 (vw)	1037.5 (2.6)	1037.4	1037.3 (vw)					1031	1031
ν_9	506.2 (28.9)	506.9 (28.8)		505.2	506.8	505.95 (s)		506.3	506.9 (s)	507.5			507.5	508.13	
$2\nu_3$	3532.9 (0.5)	3532.6 (0.5)	3512.9 (0.4)			3512.1	3524.7 (1.3)		3524.1					3529	3529
$\nu_3 + \nu_5$	2956.8 (1.7)	2957.0 (1.7)	2961.6 (2.8)				2952.4 (4.7)							2954	2954
$\nu_5 + \nu_6 + \nu_7$	2693.8 (0.1)	2696.1 (0.0)	2677.8 (1.0)				2696.5 (0.3)								2692
$2\nu_9 + \nu_5$	2179.2 (0.6)	2181.6 (0.0)	2181.1 (0.2)				2177.4 (0.4)							2178.8	
$\nu_5 + \nu_6$	2142.2 (0.6)	2144.2 (0.7)	2144.3 (0.2)				2140.2 (0.5)							2142.4	
$2\nu_9$	1009.5 (16.4)	1009.5 (14.7)	1009.3 (8.0)	1009.4	1009.2		1009.7 (20.9)							1011.68	1010

The references are abbreviated as [AutYY], where Aut denotes the first three letters of the first author's surname and YY the last two digits of the publication year. Qualitative intensities: vs = very strong, s = strong, m = medium, w = weak, vw = very weak; .

[a] Tentative assignment.

Table S3: Calculated (VCI, VPT2) and experimental vibrational fundamentals, overtones, and combination bands (cm^{-1}) of the *trans*-**DCOOH** monomer in argon and neon matrices and in the gas phase. Relative intensities, normalized to the strongest band (100), are given in parentheses where available.

	Calculation		Argon matrix				Neon matrix		Gas phase			
Mode	VCI	VPT2	[Din26]	[Hen87]	[Lun94]	[Maç03]	[Din26]	[Red77]	[Red77] (comp.)	[Ber86]	[Nej22] (IR, comp.)	[Nej22] (Raman jet)
ν_1	3574.0 (19.3)	3568.8 (35.0)	3551.5 (37.0)	3553	3551.5	3550.8 (m)	3570.4 (29.5)	3569.5	3570	3566	3566	3569
ν_2	2219.6 (21.4)	2227.3 (15.8)	2225.3 (9.4)	2227	2225.5	2225.2 (m)	2215.9 (29.0)	2221	2220	2218	2219.69	2219
ν_3	1764.2 (62.1)	1766.9 (72.1)	1761.3 (17.4)	1760.3	1761.5	1760.6 (s,vs)	1763.1 (88.2)	1762.6		1760	1762.9	1762
ν_4	1205.6 (3.2)	1243.9 (0.0)	1200.1 (6.1)	1200.9	1200.4	1199.95 (w)	1203.0 (6.2)	1202.9			1206	1206
ν_5	1143.1 (100.0)	1143.5 (100.0)	1141.9 (90.3)	1142.6	1141.7	1141.9 (s)	1141.1 (100.0)	1140.8	1142	1140	1142.31	1142
ν_6	970.7 (23.5)	974.3 (23.2)	975.2 (37.8)	974.6	976	975.15 (s)	971.6 (38.2)	971.4	970		970.89	971
ν_7	620.5 (18.1)	621.5 (18.5)	622.9 (39.9)	623.6	623.5	622.9 (m)	620.6 (29.4)	620.4	620	620	620.57	620
ν_8	872.8 (0.3)	876.5 (0.2)	874.9 (1.0)	875.1	875.7	874.9 (w)	875.1 (1.2)	874.8	870		873.38	
ν_9	632.8 (61.0)	627.3 (61.0)	626.2 (64.8)	625.7	627.8	626.15 (m)	628.7 (73.7)	628.3	629		631.54	
$2\nu_3$	3475.0 (1.0)	3482.3 (0.3)	3461.8 (0.6)			3461.3	3470.1 (1.1)					
$\nu_5 + \nu_3$	2900.3 (0.4)	2903.2 (0.0)	2896.1 (0.2)				2897.6 (0.4)					2898
$2\nu_8 + \nu_5$	2861.0 (0.4)	2864.6 (0.4)	2857.7 (0.4)				2859.6 (0.8)					2860
$\nu_6 + \nu_5$	2103.0 (0.8)	2107.3 (1.8)	2106.2 (0.3)				2102.0 (1.0)					2103
$2\nu_6$	1936.7 (0.6)	1942.8 (1.6)	1943.7 (0.3)				1938.5 (0.7)					1937
$\nu_4 + \nu_7$	1925.0 (0.3)	1925.7 (0.0)	1918.2 (0.2)				1917.2 (0.2)					1919
$2\nu_8$ [a]	1725.8 (64.2)	1728.8 (65.0)	1723.2 (100.0)	1722.8	1722.8	1723.3 (m,vs)	1726.2 (99.3)	1725.7	1738.7		1725.87	1725
$2\nu_7$	1298.4 (0.1)	1294.3 (0.0)										

The references are abbreviated as [AutYY], where Aut denotes the first three letters of the first author's surname and YY the last two digits of the publication year. Qualitative intensities: vs = very strong, s = strong, m = medium, w = weak; .

[a] See text.

Table S4: Calculated (VCI, VPT2) and experimental vibrational fundamentals, overtones, and combination bands (cm^{-1}) of the *trans*-**DCOOD** monomer in argon and neon matrices and in the gas phase. Relative intensities, normalized to the strongest band (100), are given in parentheses where available.

	Calculation		Argon matrix			Neon matrix		Gas phase				
Mode	VCI	VPT2	[Din26]	[Hen87]	[Lun94]	[Din26]	[Red77]	[Red77] (comp.)	[Ber82]	[Mare87]	[Nej22] (IR, comp.)	[Nej22] (Raman jet)
ν_1	2632.8 (22.4)	2631.1 (26.1)	2619.5 (29.4)	2620	2619.8	2632.2 (27.7)	2632.1	2632	2631.9	2635	2631.87	2632
ν_2	2231.0 (19.4)	2239.0 (17.1)	2238.1 (6.1)	2238	2237.8	2229.1 (21.2)	2200	2232	2231.8	2225	2232	2231
ν_3	1762.3 (95.4)	1735.1 (66.0)	1759.5 (59.5)	1758.9	1760.9	1761.9 (65.7)	1761.2	1742	1760	1740	1760	1761
ν_4	1172.2 (100.0)	1172.0 (100.0)	1175.2 (39.1)	1175.2	1175	1170.8 (100.0)	1170.5	1171	1169.7	1168	1170.8	1170
ν_5	1039.1 (5.2)	1039.4 (4.6)	1039.4 (4.1)	1033.8		1039.3 (6.9)	1031.9	1040	1042	1035	1042	1039
ν_6	946.2 (31.4)	947.1 (32.1)	945.75 (32.9)	945.6	946.1	945.0 (38.0)	944.9	945	945	946	945	945
ν_7	554.5 (23.8)	554.7 (24.3)	555.3 (39.5)	556.3	555.9	554.8 (18.6)	554.9	558	555.5	536	554.44	554
ν_8	873.0 (0.6)	876.7 (0.6)	874.9 (0.9)	875.1	873.1	875.0 (1.7)	874.8	873			873.2	
ν_9	490.4 (46.1)	491.5 (46.5)		489.8	489.8		490.4	491			492.23	
$2\nu_3$	3472.0 (1.4)	3456.0 (0.2)	3459.46 (0.5)			3466.9 (1.4)						
$\nu_3 + \nu_4$	2929.0 (0.2)	2924.1 (0.2)	2929.6 (0.1)			2927.4 (0.3)						2926
$\nu_4 + 2\nu_8$	2890.3 (0.3)	2899.2 (0.2)	2892.5 (0.1)			2889.5 (0.3)						2888
$\nu_3 + \nu_6$	2706.4 (0.4)	2702.9 (0.0)				2704.5 (0.2)						2704
$\nu_6 + 2\nu_8$	2669.8 (0.7)	2677.2 (0.4)	2661.1 (0.2)			2667.9 (1.1)						2668
$\nu_4 + \nu_5$	2195.2 (9.1)	2198.4 (4.3)	2198.7 (3.0)		2198.5	2193.0 (11.8)	2192.5		2195.1		2194	2194
$\nu_4 + \nu_6$	2109.6 (1.3)	2116.7 (2.9)	2112.4 (0.7)			2107.3 (1.2)						2108
$2\nu_5$	2072.6 (0.4)	2071.8 (1.7)	2072.5 (0.5)			2072.8 (2.0)						2073
$2\nu_8$	1725.5 (50.0)	1711.9 (23.4)	1720.8 (100.0)	1720.4	1720.5	1725.5 (78.7)	1725.5	1742	1723.5		1725.12	1725
$2\nu_9$	962.9 (6.3)	963.3 (4.5)	962.4 (5.5)	962.2	962.4	962.9 (9.6)	n.o.[a]				965	965

The references are abbreviated as [AutYY], where Aut denotes the first three letters of the first author's surname and YY the last two digits of the publication year.
.

[a] A band at 1020.4 cm^{-1} is reported in the original work; the assignment is unclear.

Table S5: Calculated (VCI, VPT2, [Din26]) and experimental vibrational fundamentals (cm^{-1}) of the formic acid dimer (**$(HCOOH)_2$**) in argon and neon matrices and in the gas phase. Calculated IR intensities are given in parentheses in km/mol; experimental relative intensities are normalized to the strongest band (100). Values from [Ito08] and [Ito15] are averaged over the two matrix sites.

	Calculation		Argon matrix					Neon matrix	Gas phase					
Mode	VCI	VPT2	[Din26]	[Hal98]	[Gan00]	[Ito08]	[Ito15]	[Din26]	[Ber82] (Raman)	[Wac87] (IR)	[Mar87] (IR)	[Zie07] (Raman jet)	[Geo04]	[Nej22] (comp.)
ν_1 (A_g)	2943.0	2949.4			3100									
ν_2 (A_g)	2960.0	2913.6			complex				2948.9					2948.9
ν_3 (A_g)	1671.0	1670.9							1669.9					1669.9
ν_4 (A_g)	1431.6	1429.3							1415					1430
ν_5 (A_g)	1375.3	1362.6							1374.8					1375
ν_6 (A_g)	1221.4	1225.5							1214					1224
ν_7 (A_g)	680.0	679.3							667.3			680		681
ν_8 (A_g)	195.5	193.2										194		194
ν_9 (A_g)	159.5	152.7							137.1			165		161
ν_{10} (B_g)	1055.2	1051.7							1059.7					1058
ν_{11} (B_g)	905.3	903.7												911
ν_{24} (B_g)	239.9	228.8							229.6			242		242
ν_{13} (A_u)	1056.0 (9.0)	1058.0 (10.7)	1070.2 (0.4)		1069.3 (6.1)	1070	1070	1066.3 (1.4)		1063.2				1069.3
ν_{14} (A_u)	935.6 (131.0)	943.5 (108.9)	939.7 (0.3)	942 (18)	939.9 (22)	943.5	942.5	942.6 (1.4)		923.4	908		922	944
ν_{15} (A_u)	166.3 (7.2)	158.9 (8.6)											168.5	168.47
ν_{16} (A_u)	67.5 (1.8)	67.9 (1.8)											69.2	69.2
ν_{17} (B_u)	3031.0 (118.5)	3094.8 (115.9)	3072 (0.2)	2992 (22)	3100	3076		3078 (0.2)		complex	3000		3084	3084
ν_{18} (B_u)	2955.0 (90.1)	2906.9 (86.8)		2950 (22)		2947				2938.4	2944		2938.5	2938.5
ν_{19} (B_u)	1741.8 (706.7)	1741.4 (723.7)	1728.9 (2.7)	1728 (100)	1728.3 (100)	1729.5	1729.5	1738.2 (3.1)		1745.7	1740		1746	1746
ν_{20} (B_u)	1403.8 (2.1)	1404.1 (2.4)	*		1445.6 (0.1)		*						1454	1407
ν_{21} (B_u)	1369.7 (36.4)	1361.6 (47.5)	1371.2 (0.4)	1373 (8)	1371.9 (21.8)	1372.5		1372.5 (0.2)		1362.2	1364		1364	1371.78
ν_{22} (B_u)	1230.0 (263.8)	1228.9 (332.6)	1227.3 (1.1)	1226 (41)	1223.5 (56.9)	1225	1225	1227.5 (5.2)		1217.1	1215		1218	1230
ν_{23} (B_u)	708.1 (35.4)	705.4 (36.6)	712.4 (0.2)	712 (5)	710.6 (17.4)		712	706.5 (0.9)		698.7	699		698	707.6
ν_{12} (B_u)	266.1 (64.6)	262.3 (63.4)								262			268	264

The references are abbreviated as [AutYY], where Aut denotes the first three letters of the first author's surname and YY the last two digits of the publication year.

Table S6: Calculated (VCI, VPT2, [Din26]) and experimental vibrational fundamentals (cm^{-1}) of the fully deuterated formic acid dimer (**$(DCOOD)_2$**) in argon and neon matrices and in the gas phase. Calculated IR intensities are given in parentheses in km/mol; experimental relative intensities are normalized to the strongest band (100).

	Calculation		Argon matrix	Neon matrix	Gas phase			
Mode	VCI	VPT2	[Din26]	[Din26]	[Ber82] (Raman)	[Mare87] (IR)	[Gut08]	[Nej22]
ν_1 (A_g)	2241.2	2236						
ν_2 (A_g)	2210.6	2208.28			2210.8			2210.8
ν_3 (A_g)	1637.3	1640.71			1647.6			1647.6
ν_4 (A_g)	1257.5	1253.76			1250			1258
ν_5 (A_g)	1093.1	1089.29			1080.7			1092
ν_6 (A_g)	991.8	989.1			989.6			992
ν_7 (A_g)	622.3	619.99			617.4			623
ν_8 (A_g)	193.0	191.61						192
ν_9 (A_g)	155.8	155.95			130			157
ν_{10} (B_g)	892.3	893.31			892			893
ν_{11} (B_g)	669.5	656.43						669
ν_{12} (B_g)	207.5	208.22			194.4			210
ν_{13} (A_u)	890.9 (0.0)	893.31	*	*				
ν_{14} (A_u)	707.1 (103.7)	710.84 (104.4)	709.5 (3.1)	709.7 (3.2)				711.7
ν_{15} (A_u)	138.1 (5.6)	131.79 (7.9)						
ν_{16} (A_u)	68.9 (1.3)	64.93 (1.6)						
ν_{17} (B_u)	2268.7 (63.7)	2272.94 (972.5)	2260.4 (0.8)	2260.2 (0.7)		2270		2270
ν_{18} (B_u)	2213.5 (86.3)	2204.42 (689.0)	2208.3 (5.4)	2218.7 (2.2)		n.m.		n.m.
ν_{19} (B_u)	1719.7 (263.2)	1716.07 (502.0)	1709.6 (9.4)	1715.4 (15.4)		1720	1717	1717
ν_{20} (B_u)	1264.1 (212.0)	1254.85 (142.6)	1255.5 (2.2)	1255.5 (6.1)		1249		1256.6
ν_{21} (B_u)	1072.4 (11.8)	1069.12 (43.5)	1072.6 (0.7)	1073.7 (1.2)		1070		1072
ν_{22} (B_u)	985.5 (64.9)	981.87 (62.9)	986.7 (4.2)	988.3 (11.6)		984		987.1
ν_{23} (B_u)	655.9 (41.9)	653.37 (320.3)	659.8 (2.3)	654.5 (1.9)		669		656
ν_{24} (B_u)	249.6 (55.4)	248.38 (403.4)						250

The references are abbreviated as [AutYY], where Aut denotes the first three letters of the first author's surname and YY the last two digits of the publication year.

3 Computational Accuracy for Different Computational Methods

For the meta-analysis in Figure 6 of the manuscript, we used the reference data listen in Tables S7 and S8. The references are abbreviated as [AutYY], where Aut denotes the first three letters of the first author's surname and YY the last two digits of the publication year:

`[Ala25]` Data from Alavi 2025.[26]

`[Avi23]` Data from Avila 2023.[27]

`[Dem07]` Data from Demaison 2007.[28]

`[Käs22]` Data from Käser 2022.[29]

`[Maç03]` Data from Maçôas 2003.[3]

`[Mar22]` Data from Martin Santa Daria 2022.[30]

`[Miz13]` Data from Mizukami 2013.[31]

`[Nej21]` Data from Nejad 2021.[32]

`[Nej22]` Data from Nejad 2022.[2]

`[Pit15]` Data from Pitsevich 2015.[33]

`[Qu16]` Data from Qu and Bowman 2016.[34]

`[Qu18]` Data from Qu and Bowman 2018.[35]

`[Ric18]` Data from Richter 2018.[36]

`[Sha22]` Data from Shanavas Rasheeda 2022.[37]

`[Tew16]` Data from Tew 2016.[38]

`[ThisWork]` Data from the present work.[11]

Table S7: Comparison of calculated vibrational fundamentals (cm^{-1}) of the *trans*-formic acid monomer obtained using different theoretical methods.

Reference [1]	Method[2]	ν_1	ν_2	ν_3	ν_4	ν_5	ν_6	ν_7	ν_8	ν_9
[Dem07]	HARM	3783	3106	1836	1429	1331	1144	634	1078	681
[Ric18]	HARM	3823	3014	1845	1416	1288	1124	655	1036	520
[Maç03]	HARM	3784	3134	1789	1427	1317	1123	632	1065	677
[Tew16]	HARM	3767	3092	1818	1412	1323	1140	632	1056	673
[ThisWork]	HARM	3761	3091	1814	1409	1318	1138	632	1055	672
[Miz13]	HARM	3573	3081	1809	1414	1318	1137	629	1048	670
[Miz13]	VSCF	3505	2915	1783	1376	1296	1123	636	1034	687
[Maç03]	VMP2	3551	2951	1757	1389	1268	1078	621	1035	598
[Tew16]	I-VSCF	3551	2918	1790	1383	1305	1129	683	1040	648
[Tew16]	I-VMP2	3553	2917	1790	1372	1289	1127	657	1042	633
[Dem07]	VPT2	3594	2949	1801	1402	1291	1114	628	1042	643
[Miz13]	VASCI	3542	2915	1783	1377	1321	1124	629	1034	635
[Tew16]	I-VCI	3575	2939	1783	1379	1222	1108	627	1034	641
[Miz13]	VASPT2	3560	2923	1776	1364	1301	1101	621	1027	625
[Miz13]	VMP2	3587	2944	1775	1365	1272	1102	624	1027	642
[Käs22]	VPT2/PN	3548	2935	1768	1375	1296	1099	622	1029	632
[Ala25]	VPT2/ANI	3572	2933	1779	1376	1300	1103	638	1033	620
[Ric18]	MCTDH	3567	2937	1774	1375	1301	1106	623	1032	637
[Nej21]	CVPT6	3568	2939	1773	1374	1300	1106	623	1032	637
[Mar22]	GENIUSH	3576	2938	1783	1379	1304	1108	627	1034	639
[Nej21]	CVPT6	3576	2940	1783	1380	1305	1108	627	1035	640
[Avi23]	GENIUSH	3576	2938	1783	1379	1304	1108	627	1034	639
[ThisWork]	VPT2	3568	2943	1779	1378	1302	1106	627	1033	636
[ThisWork]	VCI	3572	2940	1778	1379	1311	1106	626	1033	642
[Nej22]	"Gas phase"	3571	2942	1777	1379	1306	1105	626	1033	641

[1] The references are abbreviated as [AutYY], where Aut denotes the first three letters of the first author's surname and YY the last two digits of the publication year.

[2] HARM: Harmonic frequency calculation. VSCF: Vibrational self-consistent field. VMP2: Vibrational Møller–Plesset perturbation theory of second order. VCI: Vibrational configuration interaction. I-VSCF/I-VMP2/I-VCI: Respective methods using internal coordinates. VPT2: Vibrational perturbation theory of second order. VASCI: Vibrational adaptive sampling configuration interaction. VASPT2: Vibrational active-space second-order perturbation theory. MCTDH: Multiconfiguration time-dependent Hartree. GENIUSH: General rovibrational code with Numerical, Internal-coordinate, User-Specified Hamiltonians.

Table S8: Comparison of calculated vibrational fundamentals (cm^{-1}) of the *trans*-formic acid dimer obtained using different theoretical methods.

**IR active** (A_u, B_u) Reference[1]	Method[2]	ν_{13}	ν_{14}	ν_{15}	ν_{16}	ν_{17}	ν_{18}	ν_{19}	ν_{20}	ν_{21}	ν_{22}	ν_{23}	ν_{24}
[Qu16]	VCI						2943	1730	1407	1365	1234	703	
[Nej22]	HARM	1100	970						1448	1406	1258	716	
[Nej22]	HARM	1099	977						1453	1405	1253	711	
[Nej22]	PyVCI+VPT2	1073	959						1415	1378	1233	709	
[Nej22]	VCI+F	1068	934						1404	1377	1232	707	
[Nej22]	DVPT2	1065	937						1400	1369	1229	705	
[Qu18]	AIMD	1089	950	168	69			1768	1428	1387	1243	708	266
[Qu18]	VCI	1091	975	209	96		2960	1761	1424	1388	1252	726	286
[Qu16]	HARM	1094	989	222	92	3303	3107	1785	1466	1396	1263	716	293
[ThisWork]	HARM	1098	983	180	69	3310	3099	1782	1455	1406	1258	714	259
[Pit15]	HARM	1098	983	180	69	3310	3099	1782	1455	1406	1258	714	274
[Qu18]	VSCF	1112	1007	250	103	2953	2942	1751	1421	1381	1248	713	277
[ThisWork]	VSCF	1113	1005	246	101	2965	2931	1751	1424	1383	1245	714	277
[Pit15]	VPT2	1075	989	184	69	2836	2872	1738	1428	1373	1234	720	276
[ThisWork]	VPT2	1058	944	159	68	3095	2907	1741	1404	1362	1229	705	262
[Sha22]	HDNNP-VPT2	1074	964	166	68	3041	2941	1745	1416	1375	1233	706	264
[ThisWork]	VCI	1056	936	166	67	3031	2955	1742	1404	1370	1230	708	266
[Nej22] [3]	"Gas phase"	1069	944	168	69	3084	2939	1746	1407	1372	1230	708	264

**Raman active** (A_g, B_g) Reference[1]	Method[2]	ν_{1}	ν_{2}	ν_{3}	ν_{4}	ν_{5}	ν_{6}	ν_{7}	ν_{8}	ν_{9}	ν_{10}	ν_{11}	ν_{12}
[Qu16]	VCI				1455	1374	1248	692	208				
[Nej22]	HARM				1481	1408	1255	693			1084	956	
[Nej22]	HARM				1478	1410	1248	684			1080	953	
[Nej22]	PyVCI+VPT2				1436	1381	1229	682			1058	930	
[Nej22]	VCI+F				1426	1379	1228	687			1059	914	
[Nej22]	DVPT2				1430	1374	1228	680			1058	909	
[Qu18]	AIMD			1660	1465	1397	1236	686	202	151	1075	933	248
[Qu18]	VCI	2990		1671	1457	1391	1252	700	213	178	1079	945	273
[Qu16]	HARM	3101	3204	1721	1492	1422	1260	691	230	183	1093	972	290
[ThisWork]	HARM	3210	3103	1718	1482	1410	1254	686	209	168	1084	957	274
[Pit15]	HARM	3210	3103	1718	1482	1410	1254	686	209	168	1084	957	259
[Qu18]	VSCF	2969	2767	1681	1458	1384	1246	688	204	171	1095	981	303
[ThisWork]	VSCF	2803	2960	1682	1455	1387	1241	685	203	170	1094	980	298
[Pit15]	VPT2	2698	2882	1644	1479	1372	1236	686	199	161	1058	958	256
[ThisWork]	VPT2	2949	2914	1671	1429	1363	1226	679	193	153	1052	904	229
[Sha22]	HDNNP-VPT2	2920	2948	1677	1433	1375	1229	682	197	164	1058	934	247
[ThisWork]	VCI	2943	2960	1671	1432	1375	1221	680	196	159	1055	905	240
[Nej22] [3]	"Gas phase"		2949	1670	1430	1375	1224	681	194	161	1058	911	242

[1] The references are abbreviated as [AutYY], where Aut denotes the first three letters of the first author's surname and YY the last two digits of the publication year.
[2] HARM: Harmonic frequency calculation. VSCF: Vibrational self-consistent field. VCI: Vibrational configuration interaction. VPT2: Vibrational perturbation theory of second order. AIMD: Ab initio molecular dynamics.
[3] This includes the reference data listed by Nejad below 1500 cm^{-1} and other references for the values above.

4 Normal Mode Decomposition of the Formic Acid Monomer

We applied the NOMODECO toolkit to perform a normal mode decomposition and derive a suitable chemist notation for the formic acid monomer and its cyclic dimer. The normal mode decomposition scheme is based on *primitive* (Wilson-type) internal coordinates (ICs), the algorithmic details can be found in our original publication and the tool as well as the source code are freely available on Github (https://github.com/KemalOenen/decomposing-vibrations).

NOMODECO was applied for the *trans*-formic acid isotopocules **HH**, **HD**, **DH**, and **DD**, with the atomic labeling shown in Figure S1. Table S9 for **HH**, Table S10 for **HD**, Table S11 for **DH** and Table S12 for **DD** show the possible internal coordinate sets generated for the respective molecules. Based on topological rules and a metric the toolkit then selects the optimal internal coordinate set for the representation of the vibrational motions and performs a normal mode decomposition. The resulting potential energy distribution (PED) matrices are displayed as contribution heatmaps in Figure S2 for **HH**, in Figure S3 for **HD**, in Figure S4 for **DH**, and in Figure S5 for **DD**, such that for each normal mode ν the respective contributions of the ICs of the optimal set are shown.

Table S13 for **HH**, Table S14 for **HD**, Table S15 for **DH**, and Table S16 for **DD** contain the harmonic frequency ω and the intrinsic frequency ω^I in cm^{-1}, together with the main contributing internal coordinates. Harmonic frequencies were computed at the HF/6-311G(d,p) level of theory using analytical differentiation for the Hessian. Each normal mode is given in the spectroscopic notation $\nu_i(\text{irrep})$. The integer indices i follow decreasing harmonic frequency, and the irreducible representation (*irrep*) corresponds to the C_s point group, taking the values A′ or A″. The spectroscopic modes are therefore grouped such that ν_1–ν_7 belong to A′, and ν_8–ν_9 to A″. Because this notation reflects ordering by harmonic frequency, isotopic shifts that interchange two modes lead to spectroscopic indices that are not directly comparable between isotopocules. From the heatmaps in Figures S2,S3,S4, and S5, we derive the chemist vibrational notation as summarized in the respective tables. This is based on the main contribution internal coordinates (ICs) to a given normal mode. The chemist notation follows the usual abbreviations: ν for stretching, δ_{ip} for in-plane deformation, and δ_{oop} for out-of-plane deformation (wagging).

Table S9: Possible internal coordinates for trans-HH

IC Type	Atom Indices
**Bonds**	(O_1, H_2), (O_2, C), (C, H_1), (O_1, C)
**In-plane Angles**	(C, O_1, H_2), (O_1, C, O_2), (O_1, C, H_1), (O_2, C, H_1)
**Out-of-plane Angles**	(C, O_1, O_2, H_1), (C, O_2, O_1, H_1), (C, H_1, O_1, O_2), (C, H_1, O_2, O_1), (C, O_2, H_1, O_1)
**Dihedrals**	(H_2, O_1, C, O_2), (H_1, C, O_1, H_2)

Table S10: Possible internal coordinates for trans-HD

Ic Type	Atom Indices
**Bonds**	(O_1, D_2), (O_2, C_1), (O_1, C_1), (C_1, H_1)
**In-plane Angles**	(C_1, O_1, D_2), (O_1, C_1, O_2), (O_1, C_1, H_1), (O_2, C_1, H_1)
**Out-of-plane Angles**	(C_1, O_1, O_2, H_1), (C_1, O_2, O_1, H_1), (C_1, H_1, O_1, O_2), (C_1, H_1, O_2, O_1)
**Dihedrals**	(D_2, O_1, C_1, O_2), (D_2, O_1, C_1, H_1)

Table S11: Possible internal coordinates for trans-DH

Ic Type	Atom Indices
**Bonds**	(O_2, C_1), (O_1, C_1), (C_1, D_1), (O_1, H_2)
**In-plane Angles**	(C_1, O_1, H_2), (O_1, C_1, O_2), (O_1, C_1, D_1), (O_2, C_1, D_1)
**Out-of-plane Angles**	(C_1, O_1, O_2, D_1), (C_1, O_2, O_1, D_1), (C_1, D_1, O_1, O_2), (C_1, D_1, O_2, O_1)
**Dihedrals**	(D_1, C_1, O_1, H_2), (H_2, O_1, C_1, O_2)

Table S12: Possible internal coordinates for trans-DD

Ic Type	Atom Indices
**Bonds**	(O_1, D_2), (O_1, C_1), (O_2, C_1), (C_1, D_1)
**In-plane Angles**	(C_1, O_1, D_2), (O_1, C_1, O_2), (O_1, C_1, D_1), (O_2, C_1, D_1)
**Out-of-plane Angles**	(C_1, O_1, O_2, D_1), (C_1, O_2, O_1, D_1), (C_1, D_1, O_1, O_2)
**Dihedrals**	(D_2, O_1, C_1, O_2), (D_1, C_1, O_1, D_2)

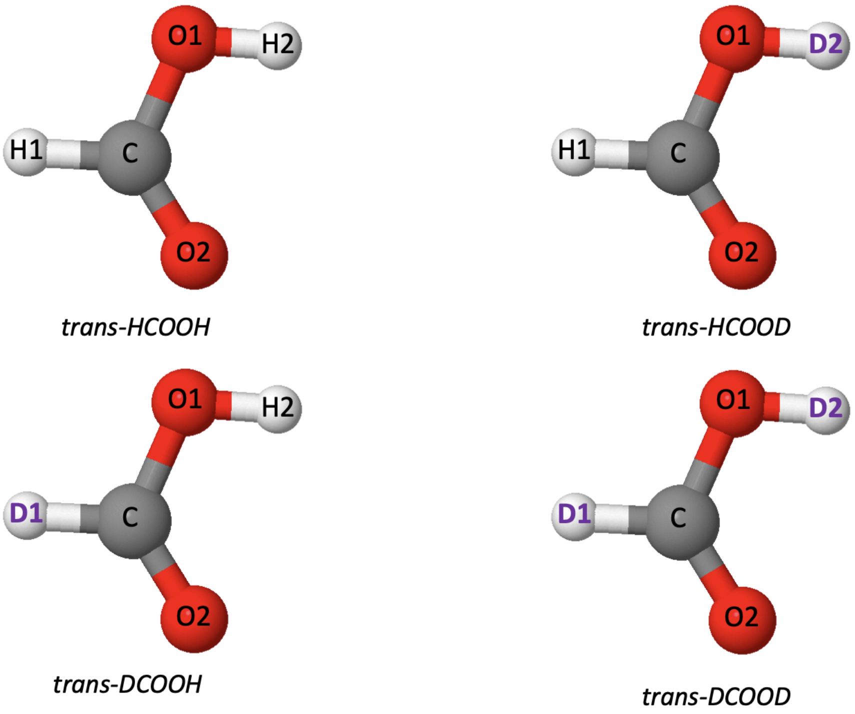

Figure S1: Atomic labeling of **HH**, **HD**, **DH** and **DD** for subsequent `NOMODECO` analysis.

4.1 Contribution heatmap and vibrational notation for trans-HH

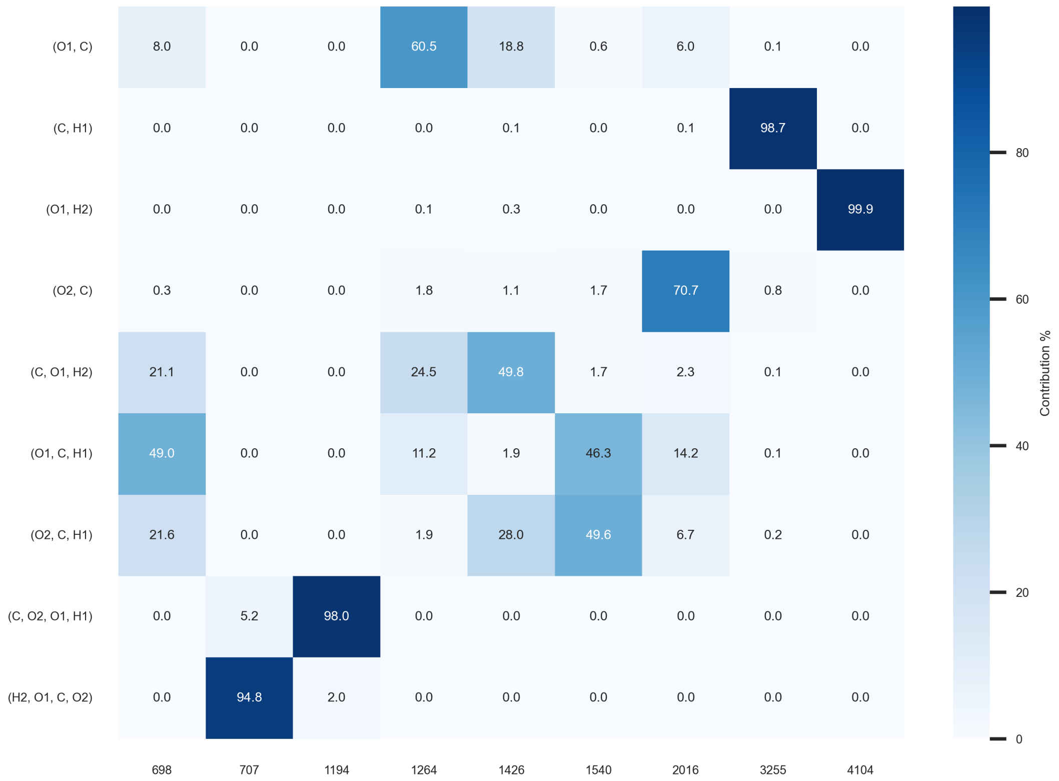

Figure S2: Contribution Table for trans-HH in C_s symmetry. Rows represent internal coordinates (ICs) and columns represent the harmonic frequencies (ω in cm^{-1}) for each normal mode ν.

Table S13: Vibrational notation for HH in C_s symmetry, determined through the analysis of the computed intrinsic frequencies and contribution table.[1]

ν_i (irrep)	ω^2	ω^I	$\lvert\omega$ - $\omega^I\rvert$	Label[3]	main contributing IC
ν_1 (A')	4104	4104	0	νOH	r(O1H2) 99.9%
ν_2 (A')	3255	3248	7	νCH	r(CH1) 98.7%
ν_3 (A')	2016	1961	55	νC=O	r(O2C) 70.7%
ν_4 (A')	1540	1291	249	δ_{ip}CH	ϕ(O2CH1) 49.6% & ϕ(O1CH1) 46.3%
ν_5 (A')	1426	1370	56	δ_{ip}COH	ϕ(OCO1H2) 49.8%
ν_6 (A')	1264	1286	22	νC−O	r(O1C) 60.5%
ν_8 (A")	1194	1341	147	δ_{oop}CH	γ(CO2O1H1) 98.0%
ν_9 (A")	707	815	108	δ_{oop}COH	τ(H2O1CO2) 94.8%
ν_7 (A')	698	1242	544	δ_{ip}OCO	ϕ(O1CH1) 49.0%

[1] For each normal mode, given in the spectroscopic notation ν together with the irreducible representation in brackets, the harmonic ω and intrinsic frequency ω^I are given in cm^{-1}, together with the main contributing internal coordinates (ICs).

[2] Harmonic frequencies ω are computed with HF/6-311g(d,p) level of theory using analytical differentiation for the computation of the Hessian.

[3] Assignment is formulated in the so-called chemist notation. Abbreviations: ν=stretch, δ_{ip}=in-plane bend

4.2 Contribution heatmap and vibrational notation for trans-HD

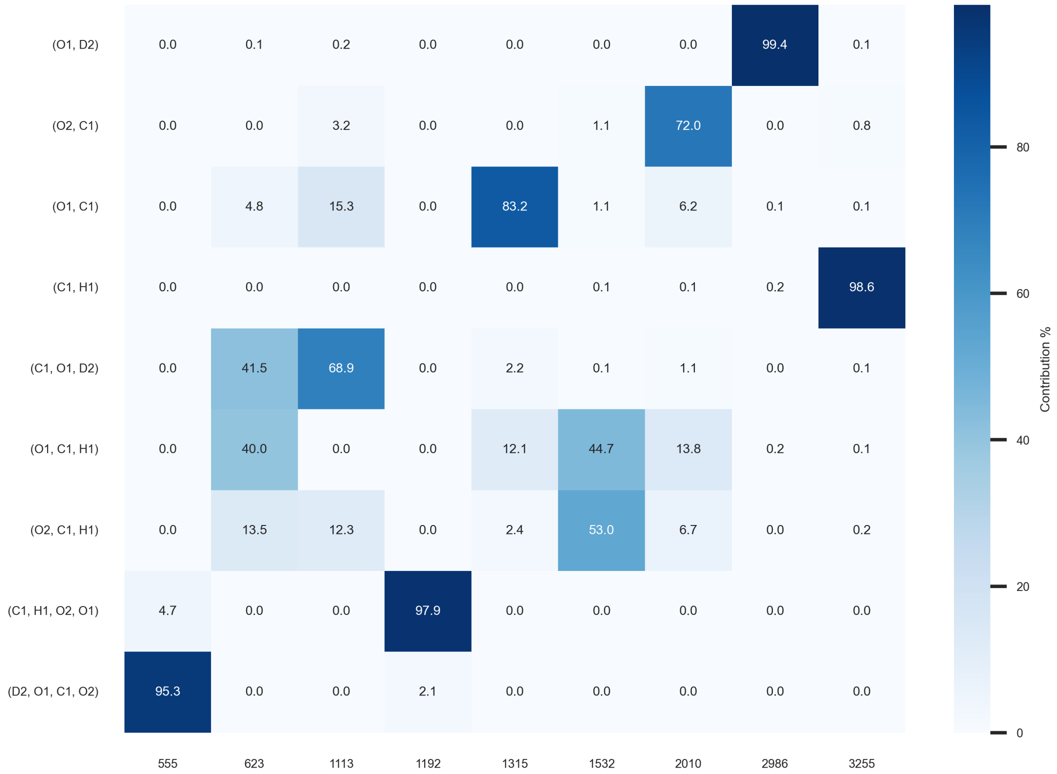

Figure S3: Contribution Table for trans-HD in C_s symmetry. Rows represent internal coordinates (ICs) and columns represent the harmonic frequencies (ω in cm^{-1}) for each normal mode ν.

Table S14: Vibrational notation for HD in C_s symmetry, determined through the analysis of the computed intrinsic frequencies and contribution table.[1]

ν_i (irrep)	ω^2	ω^I	$\lvert\omega$ - $\omega^I\rvert$	Label[3]	main contributing IC
ν_1 (A')	3255	3248	7	νCH	r(C1H1) 98.6%
ν_2 (A')	2986	2986	0	νOD	r(O1D2) 99.4%
ν_3 (A')	2010	1962	48	νC=O	r(O2C) 72.0%
ν_4 (A')	1532	1291	241	δ_{ip}CH	ϕ(O2C1H1) 53.0% & ϕ(O1CH1) 44.7%
ν_5 (A')	1315	1286	29	νC−O	r(O1C1) 83.2%
ν_8 (A")	1192	1320	128	δ_{oop}CH	γ(CO2O1H1) 97.9%
ν_6 (A')	1113	1039	74	δ_{ip}COD	ϕ(C1O1D2) 68.9%
ν_7 (A')	623	1039	416	δ_{ip}OCO	ϕ(C1O1D2) 41.5% & ϕ(O1C1H1) 40.0%
ν_9 (A")	555	661	106	δ_{oop}COD	τ(D2O1C1O2) 95.3%

[1] For each normal mode, given in the spectroscopic notation ν together with the irreducible representation in brackets, , the harmonic ω and intrinsic frequency ω^I are given in cm^{-1}, together with the main contributing internal coordinates (ICs).
[2] Harmonic frequencies ω are computed with HF/6-311g(d,p) level of theory using analytical differentiation for the computation of the Hessian.
[3] Assignment is formulated in the so-called chemist notation. Abbreviations: ν=stretch, δ_{ip}=in-plane bend

4.3 Contribution heatmap and vibrational notation for trans-DH

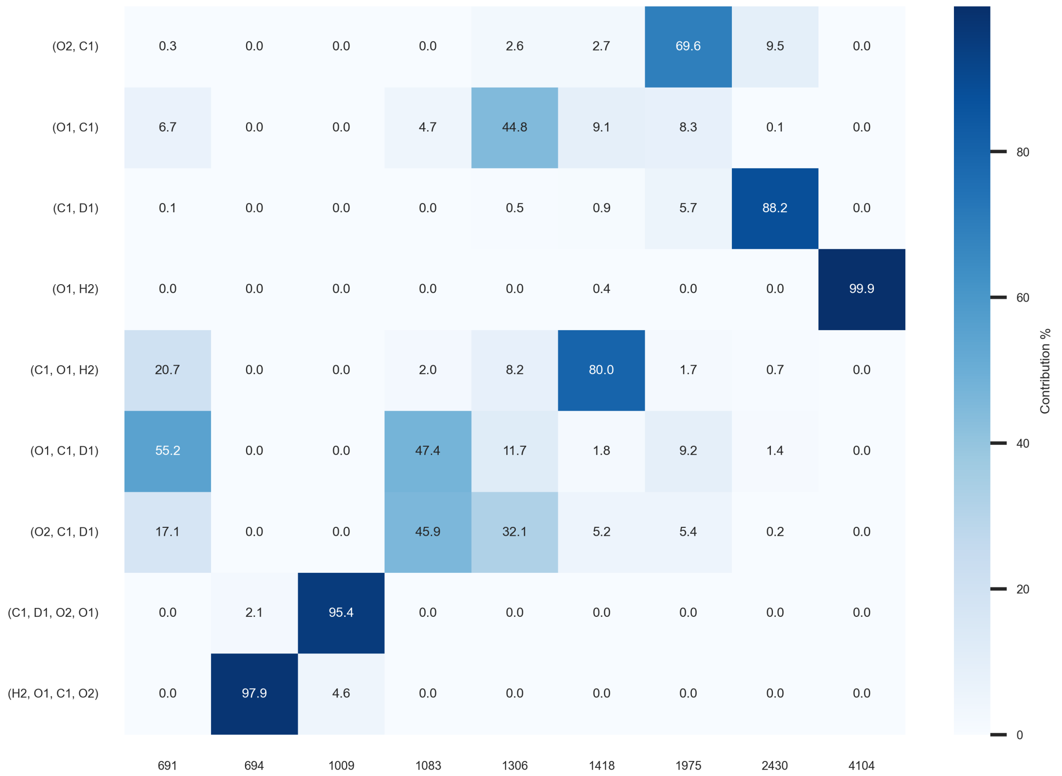

Figure S4: Contribution Table for trans-DH in C_s symmetry. Rows represent internal coordinates (ICs) and columns represent the harmonic frequencies (ω in cm^{-1}) for each normal mode ν.

Table S15: Vibrational notation for DH in C_s symmetry, determined through the analysis of the computed intrinsic frequencies and contribution table.[1]

ν_i (irrep)	ω^2	ω^I	$\|\omega$ - $\omega^I\|$	Label[3]	main contributing IC
ν_1 (A')	4104	4104	0	νOH	r(O1H2) 99.9%
ν_2 (A')	2430	2377	53	νCD	r(CD1) 88.2%
ν_3 (A')	1975	1961	14	νC=O	r(O2C) 69.6%
ν_4 (A')	1418	1370	48	δ_{ip}OH	ϕ(C1O1H2) 80.0%
ν_5 (A')	1306	1286	20	νC−O	r(O1C1) 44.8% & ϕ(O2C1D1) 32.1%
ν_6 (A')	1083	990	93	δ_{ip}**DCO**	ϕ(O1C1D1) 47.4% & ϕ(O2D1D1) 45.9%
ν_8 (A")	1009	1078	69	δ_{oop}CD	γ(C1D1O2O1) 95.4%
ν_9 (A")	694	808	114	δ_{oop}**OH**	τ(H2O1C1O2) 97.9%
ν_7 (A')	691	990	299	δ_{ip}OCO	ϕ(O1C1D1) 55.2%

[1] For each normal mode, given in the spectroscopic notation ν together with the irreducible representation in brackets, , the harmonic ω and intrinsic frequency ω^I are given in cm^{-1}, together with the main contributing internal coordinates (ICs).

[2] Harmonic frequencies ω are computed with HF/6-311g(d,p) level of theory using analytical differentiation for the computation of the Hessian.

[3] Assignment is formulated in the so-called chemist notation. Abbreviations: ν=stretch, δ_{ip}=in-plane bend

4.4 Contribution heatmap and vibrational notation for trans-DD

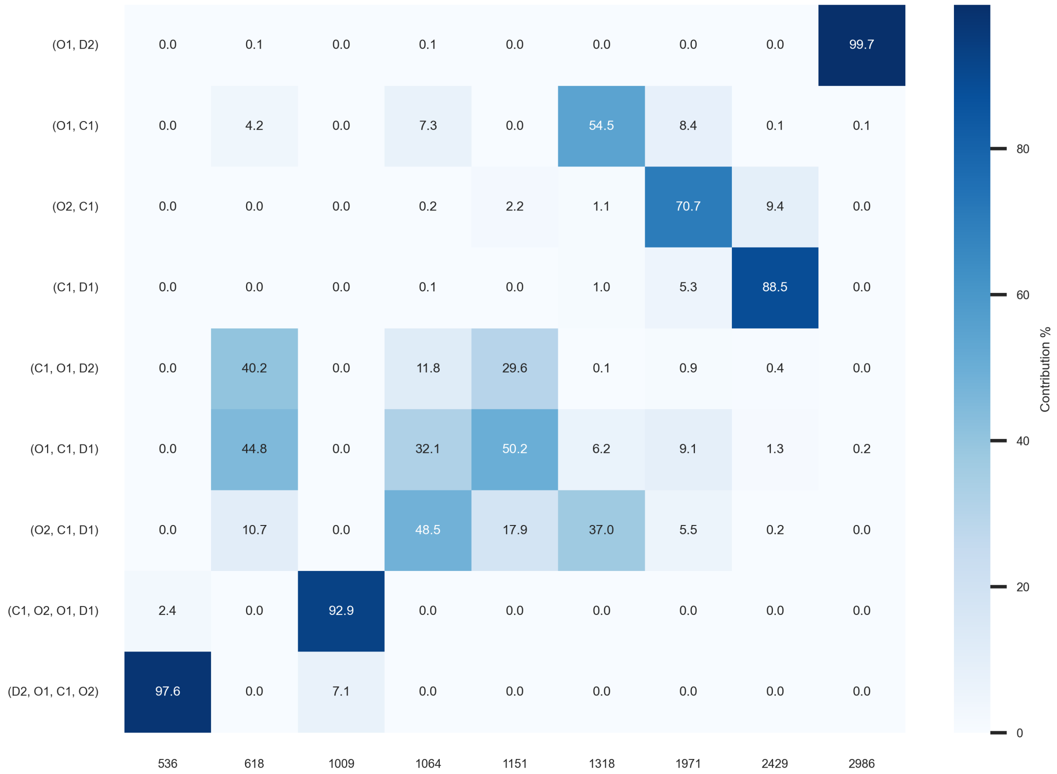

Figure S5: Contribution Table for trans-DD in C_s symmetry. Rows represent internal coordinates (ICs) and columns represent the harmonic frequencies (ω in cm^{-1}) for each normal mode ν.

Table S16: Vibrational notation for DD in C_s symmetry, determined through the analysis of the computed intrinsic frequencies and contribution table.[1]

ν_i (irrep)	ω^2	ω^I	$\lvert\omega - \omega^I\rvert$	Label[3]	main contributing IC
ν_1 (A')	2986	2985	1	νOD	r(O1D1) 99.7%
ν_2 (A')	2429	2377	52	νCD	r(C1D1) 88.5%
ν_3 (A')	1971	1962	9	νC=O	r(O2C1) 70.7%
ν_4 (A')	1318	1286	32	νC−O	r(O1C1) 54.5% & ϕ(O2C1D1) 37.0%
ν_5 (A')	1151	990	161	δ_{ip}**OD**	ϕ(O1C1D1) 50.2% & ϕ(C1O1D2) 29.6%
ν_6 (A')	1064	1075	11	δ_{ip}CD	ϕ(O2C1D1) 48.5% & ϕ(O1C1D1) 32.1%
ν_8 (A")	1009	937	72	δ_{oop}CD	γ(C1O2O1D1) 92.9%
ν_7 (A')	618	990	372	δ_{ip}OCO	ϕ(O1C1D1) 44.8% & ϕ(C1O1D2) 40.2%
ν_9 (A")	536	585	49	δ_{oop}COD	τ(D2O1C1O2) 97.6%

[1] For each normal mode, given in the spectroscopic notation ν together with the irreducible representation in brackets, , the harmonic ω and intrinsic frequency ω^I are given in cm^{-1}, together with the main contributing internal coordinates (ICs).

[2] Harmonic frequencies ω are computed with HF/6-311g(d,p) level of theory using analytical differentiation for the computation of the Hessian.

[3] Assignment is formulated in the so-called chemist notation. Abbreviations: ν=stretch, δ_{ip}=in-plane bend

5 Normal Mode Decomposition of the Formic Acid Cyclic Dimer

Considering the formic acid dimer **(HH)$_2$** and its deuterated counterpart **(DD)$_2$** additional ICs spanned by between Acceptor$\cdots$H atoms of the hydrogen bonds as well as the Acceptor$\cdots$Donor atoms were considered in order to generate a complete and non-redundant coordinate set. The internal coordinates used are as follows:

- r bond-stretching coordinate
- ϕ in-plane angle bending coordinate
- ϕ' linear angle bending coordinate
- τ dihedral angle/torsion coordinate
- γ out-of-plane bending coordinate

The structures of the cyclic dimer species (**(HH)$_2$**, **(HH)$_2$**) are shown in Figure S6. In order to generate a complete set of ICs for these hydrogen-bonded systems additional intermolecular ICs are needed. These are listed in Table S17 for **(HH)$_2$** and Table S18 for **(DD)$_2$**. The resulting potential energy distribution (PED) matrices are displayed as contribution heatmaps in Figure S7 for **(HH)$_2$** and in Figure S8 for **(DD)$_2$**, such that for each normal mode ν the respective contributions of the ICs of the optimal set are shown. Table S19 for **(HH)$_2$** and Table S20 for **(DD)$_2$** contain the harmonic frequency ω and the intrinsic frequency ω^I in cm^{-1}, together with the main contributing internal coordinates.

Table S17: Possible intermolecular internal coordinates for **(HH)$_2$**

IC Type	Atom Indices
**Hydrogen bonds (HB)**	(H_4, O_2), (H_2, O_4)
**Acceptor donor bonds (AD)**	(O_1, O_4), (O_2, O_3)
**HB in-plane angles**	(H_4, O_2, C_1), (H_2, O_4, C_2)
**AD in-plane angles**	(O_1, O_4, C_2), (C_2, O_3, O_2), (C_1, O_2, O_3), (O_4, O_1, C_1)
**HB linear valence-angles**	(O_4, H_2, O_1), (O_2, H_4, O_3), (O_4, H_2, O_1), (O_2, H_4, O_3)
**HB dihedrals**	(C_2, O_4, H_2, O_1), (H_4, O_2, C_1, O_1), (C_2, O_3, H_4, O_2), (C_1, O_1, H_2, O_4), (C_1, O_2, H_4, O_3), (H_2, O_4, C_2, H_3), (H_2, O_4, C_2, O_3), (H_1, C_1, O_2, H_4)
**AD dihedrals**	(C_1, O_1, O_4, C_2), (O_2, O_3, C_2, O_4), (O_2, C_1, O_1, O_4), (H_1, C_1, O_2, O_3), (H_1, C_1, O_1, O_4), (O_1, O_4, C_2, O_3), (O_1, C_1, O_2, O_3), (H_3, C_2, O_4, O_1), (H_3, C_2, O_3, O_2), (C_1, O_2, O_3, C_2)
**HB out-of-plane angles**	(C_1, O_1, H_1, O_2), (C_2, O_4, H_3, O_3), (C_2, H_3, O_4, O_3)
**AD out-of-plane angles**	(C_1, O_1, H_1, O_2), (O_3, O_2, H_4, C_2), (C_2, O_4, H_3, O_3), (C_2, H_3, O_4, O_3), (O_1, H_2, O_4, C_1), (O_3, H_4, C_2, O_2), (O_3, C_2, H_4, O_2), (O_1, C_1, O_4, H_2), (O_1, O_4, C_1, H_2)

Table S18: Possible intermolecular internal coordinates for **(DD)$_2$**

IC Type	Atom indices
**Hydrogen bonds (HD)**	(D_4, O_2), (D_2, O_4)
**Acceptor donor bonds (AD)**	(O_1, O_4), (O_2, O_3)
**HB in-plane angles**	(D_4, O_2, C_1), (C_2, O_4, D_2)
**AD in-plane angles**	(C_1, O_1, O_4), (O_1, O_4, C_2), (O_2, O_3, C_2), (O_3, O_2, C_1)
**HB linear valence-angles**	(O_1, D_2, O_4), (O_2, D_4, O_3), (O_1, D_2, O_4), (O_2, D_4, O_3)
**HB dihedrals**	(C_1, O_2, D_4, O_3), (D_1, C_1, O_2, D_4), (C_2, O_3, D_4, O_2), (D_4, O_2, C_1, O_1), (C_1, O_1, D_2, O_4), (C_2, O_4, D_2, O_1), (D_2, O_4, C_2, O_3), (D_2, O_4, C_2, D_3)
**AD dihedrals**	(O_1, O_4, C_2, O_3), (C_1, O_2, O_3, C_2), (O_2, O_3, C_2, O_4), (D_1, C_1, O_2, O_3), (O_2, C_1, O_1, O_4), (D_1, C_1, O_1, O_4), (D_3, C_2, O_4, O_1), (O_1, C_1, O_2, O_3), (C_1, O_1, O_4, C_2), (D_3, C_2, O_3, O_2)
**HB out-of-plane angles**	(C_2, O_3, D_3, O_4)
**AD out-of-plane angles**	(O_1, O_4, C_1, D_2), (O_3, O_2, C_2, D_4), (O_1, C_1, O_4, D_2), (C_2, O_3, D_3, O_4), (O_3, D_4, O_2, C_2), (O_3, C_2, O_2, D_4), (O_1, D_2, C_1, O_4)

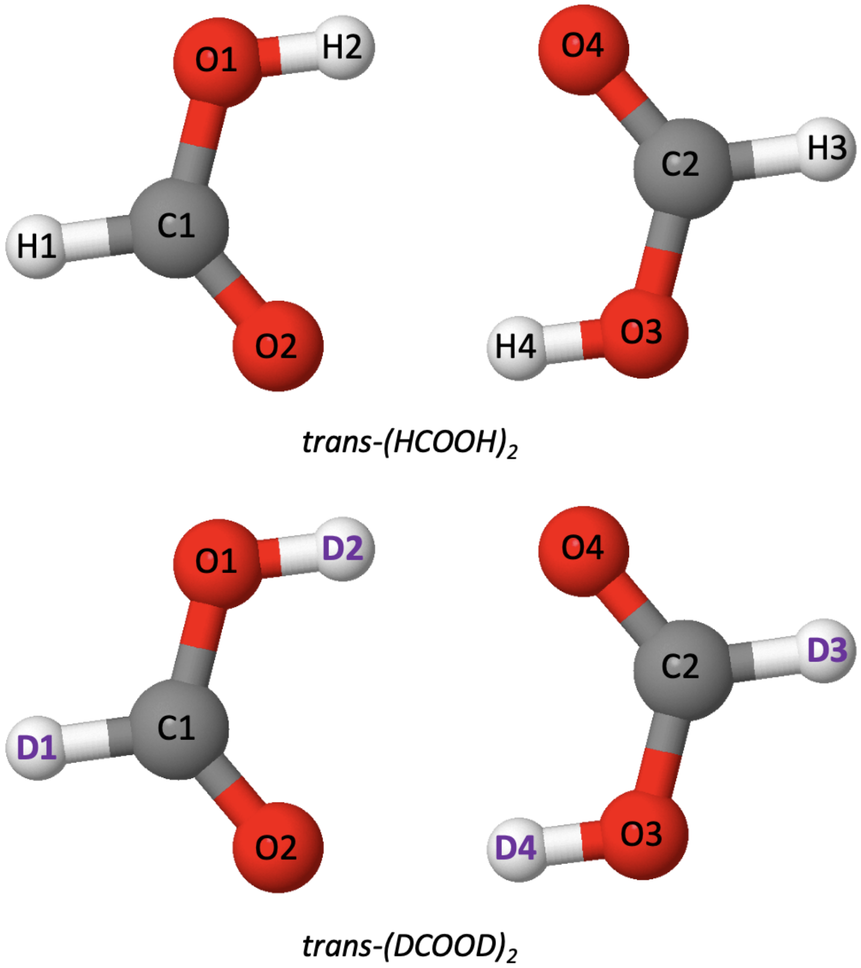

Figure S6: Labelling of **(HH)$_2$** and **(DD)$_2$** for subsequent NOMODECO analysis

5.1 Contribution heatmap and vibrational notation for (*trans*−HH)$_2$

IC	75	164	167	179	219	244	737	750	910	958	1210	1213	1346	1350	1499	1519	1553	1563	1926	1978	3263	3266	3818	3864
(O1, O4)	0.0	40.6	0.0	53.5	45.7	0.0	16.2	1.0	0.0	0.0	0.0	0.0	5.0	0.0	0.6	4.3	5.9	0.0	5.1	3.5	0.0	0.0	0.0	0.0
(C2, O3)	0.0	0.0	0.0	0.0	0.0	0.0	0.7	1.3	0.0	0.0	0.0	0.0	11.8	11.2	2.9	2.5	0.1	0.0	2.8	3.5	0.1	0.1	0.0	0.0
(C2, O4)	0.0	0.0	0.0	0.0	0.0	0.0	0.1	0.2	0.0	0.0	0.0	0.0	0.4	0.7	0.0	0.0	1.3	1.5	23.9	24.0	0.3	0.3	0.0	0.0
(C2, H3)	0.0	0.0	0.0	0.0	0.0	0.0	0.0	0.0	0.0	0.0	0.0	0.0	0.0	0.0	0.0	0.0	0.0	0.0	0.0	0.0	45.9	48.8	0.0	0.0
(O3, H4)	0.0	0.0	0.0	0.0	0.0	0.0	0.0	0.0	0.0	0.0	0.0	0.0	0.0	0.0	0.0	0.1	0.0	0.0	0.1	0.0	0.1	0.0	1.1	1.1
(C1, O1)	0.0	0.0	0.0	0.0	0.0	0.0	0.7	1.3	0.0	0.0	0.0	0.0	11.9	11.3	2.9	2.5	0.1	0.0	2.8	3.5	0.1	0.1	0.0	0.0
(C1, O2)	0.0	0.0	0.0	0.0	0.0	0.0	0.1	0.2	0.0	0.0	0.0	0.0	0.4	0.7	0.0	0.0	1.3	1.5	23.1	23.2	0.3	0.3	0.0	0.0
(C1, H1)	0.0	0.0	0.0	0.0	0.0	0.0	0.0	0.0	0.0	0.0	0.0	0.0	0.0	0.0	0.0	0.0	0.0	0.0	0.0	0.0	45.9	48.8	0.0	0.0
(O1, H2)	0.0	0.3	0.0	0.1	0.0	0.0	0.2	0.7	0.0	0.0	0.0	0.0	0.1	1.6	1.7	3.3	0.3	0.7	6.1	0.2	3.5	0.6	51.3	51.3
(H2, O4, C2)	0.0	1.8	0.0	0.3	0.1	0.0	0.1	11.4	0.0	0.0	0.0	0.0	0.1	1.2	0.5	1.9	0.4	1.1	0.0	0.2	0.0	0.0	0.0	0.0
(O3, C2, H3)	0.0	0.0	0.0	0.0	0.0	0.0	3.9	6.6	0.0	0.0	0.0	0.0	3.9	4.8	1.6	8.2	19.8	12.0	7.2	7.5	0.1	0.1	0.0	0.0
(O4, C2, H3)	0.0	0.0	0.0	0.0	0.0	0.0	2.1	4.2	0.0	0.0	0.0	0.0	0.6	1.1	7.6	17.2	20.7	9.8	3.4	4.2	0.1	0.1	0.0	0.0
(C2, O3, H4)	0.0	0.1	0.0	0.0	0.0	0.0	0.6	0.8	0.0	0.0	0.0	0.0	3.3	4.7	8.1	4.3	2.0	4.8	1.8	1.3	0.0	0.0	0.0	0.0
(O1, C1, H1)	0.0	0.0	0.0	0.0	0.0	0.0	3.8	6.5	0.0	0.0	0.0	0.0	3.8	4.7	1.6	8.0	19.3	11.7	7.0	7.4	0.1	0.1	0.0	0.0
(O2, C1, H1)	0.0	0.0	0.0	0.0	0.0	0.0	2.0	4.0	0.0	0.0	0.0	0.0	0.6	1.0	7.3	16.4	19.7	9.3	3.3	4.0	0.1	0.1	0.0	0.0
(C1, O1, H2)	0.0	0.3	0.0	0.0	0.0	0.0	1.5	1.9	0.0	0.0	0.0	0.0	8.4	12.0	20.6	11.1	5.2	12.3	4.5	3.2	0.1	0.1	0.0	0.0
(O4, H2, O1)$^{\alpha}$	0.0	56.2	0.8	45.8	54.0	0.3	64.0	47.9	0.8	0.7	0.0	0.0	44.6	38.9	35.6	15.2	1.5	28.6	8.2	13.4	3.5	0.5	47.5	47.5
(O4, H2, O1)$^{\beta}$	0.0	0.7	51.7	0.2	0.2	17.7	4.0	12.0	46.8	41.1	1.5	2.0	5.1	6.2	9.1	5.0	2.4	6.4	0.7	0.8	0.0	0.0	0.0	0.0
(C2, H3, O3, O4)	4.3	0.0	24.9	0.0	0.0	9.6	0.0	0.0	0.1	0.1	47.4	51.8	0.0	0.0	0.0	0.0	0.0	0.0	0.0	0.0	0.0	0.0	0.0	0.0
(C1, H1, O1, O2)	3.7	0.0	21.3	0.0	0.0	8.2	0.0	0.0	0.1	0.1	40.5	44.3	0.0	0.0	0.0	0.0	0.0	0.0	0.0	0.0	0.0	0.0	0.0	0.0
(H2, O4, C2, O3)	5.0	0.0	1.0	0.0	0.0	54.0	0.0	0.0	10.2	19.2	6.9	0.7	0.0	0.0	0.0	0.0	0.0	0.0	0.0	0.0	0.0	0.0	0.0	0.0
(C1, O1, O4, C2)	47.3	0.0	0.3	0.0	0.0	7.2	0.0	0.0	0.1	0.0	1.7	0.1	0.0	0.0	0.0	0.0	0.0	0.0	0.0	0.0	0.0	0.0	0.0	0.0
(H4, O3, C2, O4)	17.6	0.0	0.0	0.0	0.0	1.3	0.0	0.0	18.6	17.1	0.8	0.4	0.0	0.0	0.0	0.0	0.0	0.0	0.0	0.0	0.0	0.0	0.0	0.0
(H2, O1, C1, O2)	22.2	0.0	0.0	0.0	0.0	1.7	0.0	0.0	23.4	21.6	1.1	0.6	0.0	0.0	0.0	0.0	0.0	0.0	0.0	0.0	0.0	0.0	0.0	0.0

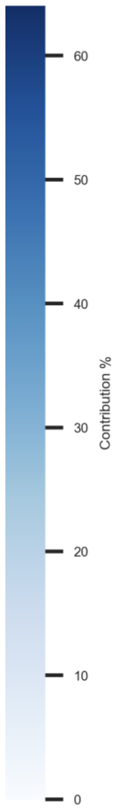

Figure S7: Contribution Table for (HH)$_2$ in C_{2h} symmetry. Rows represent internal coordinates (ICs) and columns represent the harmonic frequencies (ω in cm^{-1}) for each normal mode ν. The linear angle coordinate ϕ'(O4H2O1) occurs twice, one in plane denoted as α and the other out-of-plane denoted as β.

Table S19: Vibrational notation for $(HH)_2$ in C_{2h} symmetry, determined through the analysis of the computed intrinsic frequencies and contribution table.[1]

ν_i (irrep)	ω^2	ω^I	$\|\omega - \omega^I\|$	Label[3]	main contributing IC
**Raman active**					
ν_1 (Ag)	3818	3522	296	νOH M1/M2	r(O1H2) 51.0% & ϕ'_α(O4H2O1) 47.5%
ν_2 (Ag)	3266	3259	7	νCH M1/M2	r(C1H1) 48.8% & r(C2H3) 48.8%
ν_3 (Ag)	1926	1892	34	νC=O M1/M2	r(C1O2) 23.1% & r(C2O4) 23.9%
ν_4 (Ag)	1563	1627	64	δ_{ip}COH M1/M2	ϕ'_α(O4H2O1) 28.6% & ϕ(C1O1H2) 12.3 % & ϕ(O3C2H3) 12.0 %
ν_5 (Ag)	1519	1319	200	δ_{ip}CH M1/M2	ϕ(O4C2H3) 17.2% & ϕ(O2C1H1) 16.4%
ν_6 (Ag)	1346	1173	173	νC−O M1/M2	r(C1O1) 11.9% & r(C2O3) 11.8%
ν_7 (Ag)	737	1627	890	δ_{ip}OCO M1/M2	ϕ'_α(O4H2O1) 64.0% + r(O1O4) 16.2%
ν_8 (Ag)	179	161	18	M1-M2 "stretch"	r(O1O4) 53.5% & ϕ'_α(O4H2O1) 45.8%
ν_9 (Ag)	164	1627	1463	M1-M2 "ip bend"	ϕ'_α(O4H2O1) 56.2% & r(O1O4) 40.6%
ν_{10} (Bg)	1210	1173	37	δ_{oop}CH M1/M2	γ(C2H3O3O4) 47.7% & γ(C1H1O1O2) 40.5%
ν_{11} (Bg)	910	269	641	δ_{oop}COH M1/M2	ϕ'_β(O4H2O1) 46.8% & τ(H2O1C1O2) 23.4%
ν_{12} (Bg)	244	777	533	M1-M2 "oop lib"	τ(H2O4C2O3) 54.0% & γ(C2H3O3O4) 24.9%
**Infrared active**					
ν_{13} (Au)	1213	1173	40	δ_{oop}CH M1/M2	γ(C2H3O3O4) 51.8% & γ(C1H1O1O2) 44.3%
ν_{14} (Au)	958	269	689	δ_{oop}COH	ϕ'_β(O4H2O1) 41.1%, τ(H2O1C1O2) 21.6%
ν_{15} (Au)	167	269	102	M1-M2 "oop bend"	ϕ'_β(O4H2O1) 51.7%
ν_{16} (Au)	75	174	99	M1-M2 "twist"	τ(C1O1O4C2) 47.5%
ν_{17} (Bu)	3864	3522	342	νOH M1/M2	r(O1H2) 51.0% & ϕ'_α(O4H2O1) 47.5%
ν_{18} (Bu)	3263	3529	4	νCH M1/M2	r(C1H1) 45.9% & r(C2H3) 45.9%
ν_{19} (Bu)	1978	1892	86	νC=O M1/M2	r(C1O2) 23.2% & r(C2O4) 24.0%
ν_{20} (Bu)	1553	1319	234	δ_{ip}CH M1/M2	ϕ(O4C2H3) 20.7% & ϕ(O3C2H3) 19.8% & ϕ(O2C1H1) 19.7%
ν_{21} (Bu)	1499	1627	128	δ_{ip}COH M1/M2	ϕ'_α(O4H2O1) 35.6% & ϕ(C1O1H2) 20.6%
ν_{22} (Bu)	1350	1627	277	νC−O M1/M2	r(C1O1) 11.3% & r(C2O3) 11.2%
ν_{23} (Bu)	750	1627	877	δ_{ip}OCO M1/M2	ϕ'_α(O4H2O1) 47.9% & ϕ(H2O4C2) 11.4%
ν_{24} (Bu)	219	1627	1408	M1-M2 "ip lib"	ϕ'_α(O4H2O1) 54.0% & r(O1O4) 45.7%

[1] For each normal mode, given in the spectroscopic notation ν together with the irreducible representation in brackets, the harmonic ω and intrinsic frequency ω^I are given in cm^{-1}, together with the main contributing internal coordinates (ICs).
[2] Harmonic frequencies ω are computed with HF/6-311g(d,p) level of theory using analytical differentiation for the computation of the Hessian.
[3] Assignment is formulated in the so-called chemist notation. Abbreviations: ν=stretch, δ_{ip}=in-plane bend

5.2 Contribution heatmap and vibrational notation for $(DD)_2$

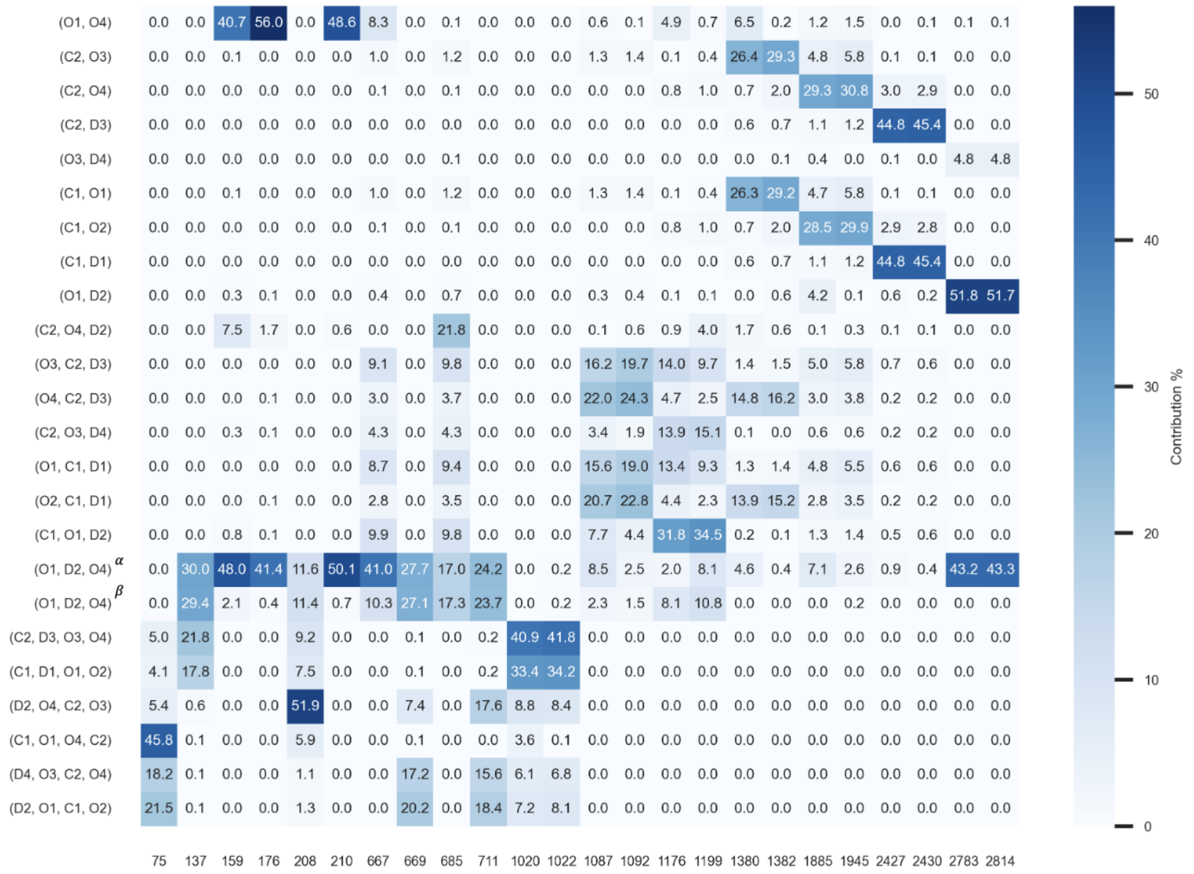

Figure S8: Contribution Table for $(DD)_2$ in C_{2h} symmetry. Rows represent internal coordinates (ICs) and columns represent the harmonic frequencies (ω in cm^{-1}) for each normal mode ν. The linear angle coordinate ϕ'(O1D2O4) occurs twice, one in plane denoted as α and the other out-of-plane denoted as β.

Table S20: Vibrational notation for $(DD)_2$ in C_{2h} symmetry, determined through the analysis of the computed intrinsic frequencies and contribution table.[1]

ν_i (irrep)	ω	ω^I	$\|\omega - \omega^I\|$	Label[2]	main contributing IC
**Raman active**					
ν_1 (Ag)	2783	2696	87	νOD M1/M2	r(O1D2) 51.8% & ϕ'_α(O1D2O4) 43.2%
ν_2 (Ag)	2430	2385	45	νCD M1/M2	r(C1D1) 45.4% & r(C2D3) 45.4&
ν_3 (Ag)	1885	1891	6	νC=O M1/M2	r(C2O4) 29.3% & r(C1O2) 28.5%
ν_4 (Ag)	1380	1375	5	νC−O M1/M2	r(C2O3) 26.4% & r(C1O1) 26.3%
ν_5 (Ag)	1199	1034	165	δ_{ip}COD M1/M2	ϕ(C1O1D2) 34.5% & ϕ(C2O3D4) 15.1%
ν_6 (Ag)	1092	1100	8	δ_{ip}CD M1/M2	ϕ(O4C2D3) 24.3% & ϕ(O2C1D1) 22.8%
ν_7 (Ag)	667	779	112	δ_{ip}OCO M1/M2	ϕ'_α(O1D2O4) 41.0%
ν_8 (Ag)	176	195	19	M1-M2 "stretch"	r(O1O4) 56.0% & ϕ'_α(O1D2O4) 41.4%
ν_9 (Ag)	159	779	620	M1-M2 "ip bend"	ϕ'_α(O1D2O4) 48.0% & r(O1O4) 40.7%
ν_{10} (Bg)	1022	947	75	δ_{oop}CD M1/M2	γ(C2D3O3O4) 41.8% & γ(C1D1O1O2) 34.2%
ν_{11} (Bg)	669	779	110	δ_{oop}COD M1/M2	ϕ'_α(O1D2O4) 27.1% & ϕ'_β(O1D2O4) 27.1%
ν_{12} (Bg)	208	689	481	M1-M2 "oop lib"	τ(D2O4C2O3) 51.9%
**Infrared active**					
ν_{13} (Au)	1020	947	73	δ_{oop}CD M1/M2	γ(C2D3O3O4) 40.9% & γ(C1D1O1O2) 33.4%
ν_{14} (Au)	711	779	68	δ_{oop}COD M1/M2	ϕ'_α(O1D2O4) 24.2% & ϕ'_β(O1D2O4) 23.7%
ν_{15} (Au)	137	779	642	M1-M2 "oop bend"	ϕ'_α(O1D2O4) 30.0% & γ(C2D3O3O4) 21.8%
ν_{16} (Au)	75	172	97	M1-M2 "twist"	τ(C1O1O4C2) 45.8% & τ(D2O1C1O2) 21.5%
ν_{17} (Bu)	2814	2696	118	νOD M1/M2	r(O1D2) 51.7% & ϕ'(O1D2O4) 43.3%
ν_{18} (Bu)	2427	2385	42	νCD M1/M2	r(C1D1) 44.8% & r(C2D3) 44.8%
ν_{19} (Bu)	1945	1891	54	νC=O M1/M2	r(C2O4) 30.8% & r(C1O2) 29.9%
ν_{20} (Bu)	1382	1375	7	νC−O M1/M2	r(C2O3) 29.3% & r(C1O1) 29.2%
ν_{21} (Bu)	1176	1034	142	δ_{ip}COD M1/M2	ϕ(C1O1D2) 31.8% & ϕ(O3C2D3) 14.0%
ν_{22} (Bu)	1087	1100	13	δ_{ip}CD M1/M2	ϕ(O4C2D3) 22.0% & ϕ(O2C1D1) 20.7%
ν_{23} (Bu)	685	231	454	δ_{ip}OCO M1/M2	ϕ(C2O4D2) 21.8% & ϕ'_β(O1D2O4) 17.3%
ν_{24} (Bu)	210	779	569	M1-M2 "ip lib"	ϕ'_α(O1D2O4) 50.1% & r(O1O4) 48.6%

[1] For each normal mode, given in the spectroscopic notation ν together with the irreducible representation in brackets, , the harmonic ω and intrinsic frequency ω^I are given in cm^{-1}, together with the main contributing internal coordinates (ICs).

[2] Assignment is formulated in the so-called chemist notation. Abbreviations: ν=stretch, δ_{ip}=in-plane bend

6 Resonance Analysis of the Monomer

In the following section the resonance analysis for the VCI results of the formic-acid monomers is presented.

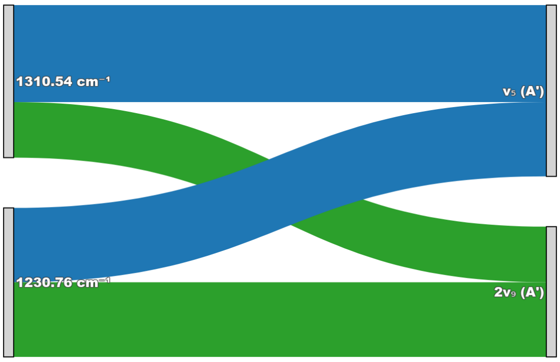

Figure S9: VCI based resonance analysis of ν_5 (A') of **HH**. The Sankey diagram displays the contribution of a fundamental (blue) or a overtone (green) transition. The thickness of the line indicates the contribution of a given state to the VCI solution.

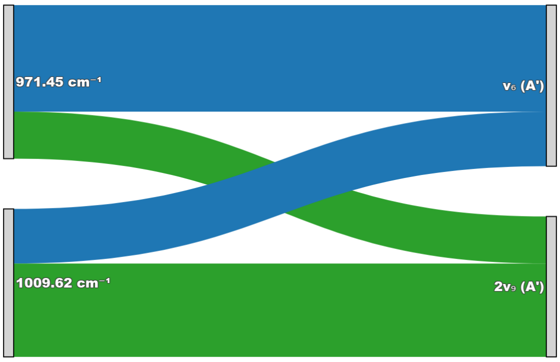

Figure S10: VCI based resonance analysis of ν_6 (A') of **HD**. The Sankey diagram displays the contribution of a fundamental (blue) or a overtone (green) transition. The thickness of the line indicates the contribution of a given state to the VCI solution.

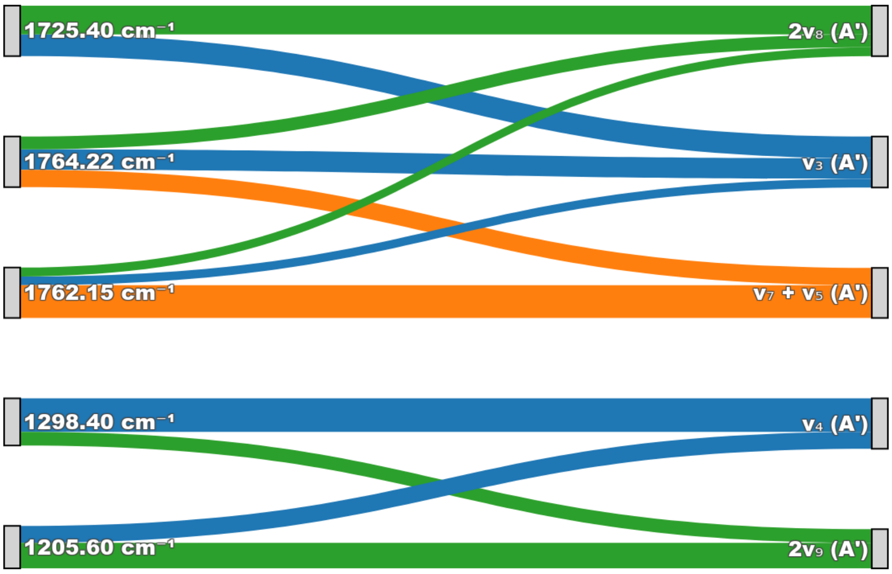

Figure S11: VCI based resonance analysis of ν_3 (A') and ν_4 (A') of **DH**. The Sankey diagram displays the contribution of a fundamental (blue), overtone (green) or combination (orange) transition. The thickness of the line indicates the contribution of a given state to the VCI solution.

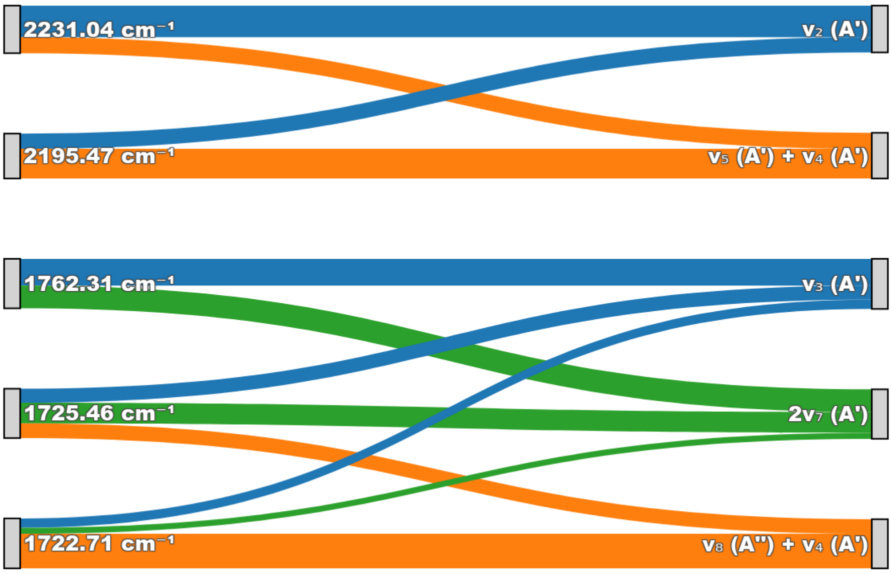

Figure S12: VCI based resonance analysis of ν_6 (A') of **DD**. The Sankey diagram displays the contribution of a fundamental (blue), overtone (green) or combination (orange) transition. The thickness of the line indicates the contribution of a given state to the VCI solution.

7 Supplementary References

Notes and references

[1] S. Lopes, R. Fausto and L. Khriachtchev, *The Journal of Chemical Physics*, 2018, **148**, 034301.

[2] A. Nejad, *PhD thesis*, Georg-August-University Göttingen, 2022.

[3] E. M. Maçôas, J. Lundell, M. Pettersson, L. Khriachtchev, R. Fausto and M. Räsänen, *Journal of Molecular Spectroscopy*, 2003, **219**, 70–80.

[4] K. Marushkevich, L. Khriachtchev, J. Lundell, A. V. Domanskaya and M. Räsänen, *Journal of Molecular Spectroscopy*, 2010, **259**, 105–110.

[5] M. Freytes, D. Hurtmans, S. Kassi, J. Liévin, J. Vander Auwera, A. Campargue and M. Herman, *Chemical Physics*, 2002, **283**, 47–61.

[6] A. Nejad, M. A. Suhm and K. A. E. Meyer, *Physical Chemistry Chemical Physics*, 2020, **22**, 25492–25501.

[7] V. Z. Williams, *The Journal of Chemical Physics*, 1947, **15**, 243–251.

[8] K. Marushkevich, L. Khriachtchev, J. Lundell, A. Domanskaya and M. Räsänen, *The Journal of Physical Chemistry A*, 2010, **114**, 3495–3502.

[9] M. Gantenberg, M. Halupka and W. Sander, *Chemistry – A European Journal*, 2000, **6**, 1865–1869.

[10] R. Georges, M. Freytes, D. Hurtmans, I. Kleiner, J. Vander Auwera and M. Herman, *Chemical Physics*, 2004, **305**, 187–196.

[11] D. F. Dinu, L. Meinschad, J. Schlagin, V. Enders, M. Podewitz, D. Stolzenburg, G. Rauhut, T. Loerting, H. Grothe and K. R. Liedl, *Phys. Chem. Chem. Phys.*, 2026.

[12] D. Henderson, *PhD thesis*, Texas Tech University, 1987.

[13] I. D. Reva, A. M. Plokhotnichenko, E. D. Radchenko, G. G. Sheina and Y. P. Blagoi, *Spectrochimica Acta Part A: Molecular Spectroscopy*, 1994, **50**, 1107–1111.

[14] J. Lundell, M. Räsänen and Z. Latajka, *Chemical Physics*, 1994, **189**, 245–260.

[15] R. L. Redington, *Journal of Molecular Spectroscopy*, 1977, **65**, 171–189.

[16] J. E. Bertie and K. H. Michaelian, *The Journal of Chemical Physics*, 1982, **76**, 886–894.

[17] J. E. Bertie, K. H. Michaelian, H. H. Eysel and D. Hager, *The Journal of Chemical Physics*, 1986, **85**, 4779–4789.

[18] M. Halupka and W. Sander, *Spectrochimica Acta Part A: Molecular and Biomolecular Spectroscopy*, 1998, **54**, 495–500.

[19] F. Ito, *Journal of Chemical Physics*, 2008, **128**, year.

[20] F. Ito, *Journal of Molecular Structure*, 2015, **1091**, 203–209.

[21] Y. Maréchal, *The Journal of Chemical Physics*, 1987, **87**, 6344–6353.

[22] T. Wachs, D. Borchardt and S. Bauer, *Spectrochimica Acta Part A: Molecular Spectroscopy*, 1987, **43**, 965–969.

[23] P. Zielke and M. A. Suhm, *Physical Chemistry Chemical Physics*, 2007, **9**, 4528.

[24] L. George and W. Sander, *Spectrochimica Acta - Part A: Molecular and Biomolecular Spectroscopy*, 2004, **60**, 3225–3232.

[25] A. Gutberlet, G. Schwaab and M. Havenith, *Chemical Physics*, 2008, **343**, 158–167.

[26] S. F. Alavi, Y. Chen, Y.-F. Hou, F. Ge, P. Zheng and P. O. Dral, *The Journal of Physical Chemistry Letters*, 2025, **16**, 483–493.

[27] G. Avila, A. Martín Santa Daría and E. Mátyus, *Physical Chemistry Chemical Physics*, 2023, **25**, 15183–15192.

[28] J. Demaison, M. Herman and J. Liévin, *The Journal of Chemical Physics*, 2007, **126**, 164305.

[29] S. Käser and M. Meuwly, *Physical Chemistry Chemical Physics*, 2022, **24**, 5269–5281.

[30] A. Martín Santa Daría, G. Avila and E. Mátyus, *Journal of Molecular Spectroscopy*, 2022, **385**, 111617.

[31] W. Mizukami and D. P. Tew, *The Journal of Chemical Physics*, 2013, **139**, 194108.

[32] A. Nejad and E. L. Sibert, *Journal of Chemical Physics*, 2021, **154**, year.

[33] G. Pitsevich, A. Malevich, E. Kozlovskaya, I. Y. Doroshenko, V. Sablinskas, V. Pogorelov, D. Dovgal and V. Balevicius, *Vibrational Spectroscopy*, 2015, **79**, 67–75.

[34] C. Qu and J. M. Bowman, *Phys. Chem. Chem. Phys.*, 2016, **18**, 24835.

[35] C. Qu and J. M. Bowman, *Faraday Discussions*, 2018, **212**, 33–49.

[36] F. Richter and P. Carbonnière, *The Journal of Chemical Physics*, 2018, **148**, 064303.

[37] D. Shanavas Rasheeda, A. Martín Santa Daría, B. Schröder, E. Mátyus and J. Behler, *Physical Chemistry Chemical Physics*, 2022, **24**, 29381–29392.

[38] D. P. Tew and W. Mizukami, *The Journal of Physical Chemistry A*, 2016, **120**, 9815–9828.
